# Temporal Patterns of Happiness and Information in a Global Social Network: Hedonometrics and Twitter

**DOI:** 10.1371/journal.pone.0026752

**Published:** 2011-12-07

**Authors:** Peter Sheridan Dodds, Kameron Decker Harris, Isabel M. Kloumann, Catherine A. Bliss, Christopher M. Danforth

**Affiliations:** 1 Department of Mathematics and Statistics, University of Vermont, Burlington, Vermont, United States of America; 2 Center for Complex Systems, University of Vermont, Burlington, Vermont, United States of America; 3 Vermont Advanced Computing Center, University of Vermont, Burlington, Vermont, United States of America; 4 Department of Physics, University of Vermont, Burlington, Vermont, United States of America; Indiana University – Bloomington, United States of America

## Abstract

Individual happiness is a fundamental societal metric. Normally measured through self-report, happiness has often been indirectly characterized and overshadowed by more readily quantifiable economic indicators such as gross domestic product. Here, we examine expressions made on the online, global microblog and social networking service Twitter, uncovering and explaining temporal variations in happiness and information levels over timescales ranging from hours to years. Our data set comprises over 46 billion words contained in nearly 4.6 billion expressions posted over a 33 month span by over 63 million unique users. In measuring happiness, we construct a tunable, real-time, remote-sensing, and non-invasive, text-based hedonometer. In building our metric, made available with this paper, we conducted a survey to obtain happiness evaluations of over 10,000 individual words, representing a tenfold size improvement over similar existing word sets. Rather than being ad hoc, our word list is chosen solely by frequency of usage, and we show how a highly robust and tunable metric can be constructed and defended.

## Introduction

One of the great modern scientific challenges we face lies in understanding macroscale sociotechnical phenomena–i.e., the behavior of decentralized, networked systems inextricably involving people, information, and machine algorithms–such as global economic crashes and the spreading of ideas and beliefs [1]. Accurate description through quantitative measurement is essential to the advancement of any scientific field, and the shift from being data scarce to data rich has revolutionized many areas [2]–[5] ranging from astronomy [6]–[8] to ecology and biology [9] to particle physics [10]. For the social sciences, the now widespread usage of the Internet has led to a collective, open recording of an enormous number of transactions, interactions, and expressions, marking a clear transition in our ability to quantitatively characterize, and thereby potentially understand, previously hidden as well as novel microscale mechanisms underlying sociotechnical systems [11].

While there are undoubtedly limits to that which may eventually be quantified regarding human behavior, recent studies have demonstrated a number of successful and diverse methodologies, all impossible (if imaginable) prior to the Internet age. Three examples relevant to public health, markets, entertainment, history, evolution of language and culture, and prediction are (1) Google's digitization of over 15 million books and an initial analysis of the last two hundred years, showing language usage changes, censorship, dynamics of fame, and time compression of collective memory [12], [13]; (2) Google's Flu Trends [14]–[16] which allows for real-time monitoring of flu outbreaks through the proxy of user search; and (3) the accurate prediction of box office success based on the rate of online mentions of individual movies [17] (see also [18]).

Out of the many possibilities in the ‘Big Data’ age of social sciences, we focus here on measuring, describing, and understanding the well-being of large populations. A measure of ‘societal happiness’ is a crucial adjunct to traditional economic measures such as gross domestic product and is of fundamental scientific interest in its own right [19]–[22].

Our overall objective is to use web-scale text analysis to remotely sense societal-scale levels of happiness using the singular source of the microblog and social networking service Twitter.

Our contributions are both methodological and observational. First, our method for measuring the happiness of a given text, which we introduced in [23] and which we improve upon greatly in the present work, entails word frequency distributions combined with independently assessed numerical estimates of the ‘happiness’ of over 10,000 words obtained using Amazon's Mechanical Turk [24]. We describe our method in full below and demonstrate its robustness. We refer to our data set as ‘language assessment by Mechanical Turk 1.0’, which abbreviates as labMT 1.0, and we provide all data as Data Set S1.

Second, using Twitter as a data source, we are able to explore happiness as a function of time, space, demographics, and network structure, with time being our focus here. Twitter is extremely simple in nature, allowing users to place brief, text-only expressions online–‘status updates’ or ‘tweets’–that are no more than 140 characters in length. As we will show, Twitter's framing tends to yield in-the-moment expressions that reflect users' current experiences, making the service an ideal candidate input signal for a real time societal ‘hedonometer’ [25].

There is an important psychological distinction between an individual's current, experiential happiness [26] and their longer term, reflective evaluation of their life [27], and in using Twitter, our approach is tuned to the former kind. Nevertheless, by following the written expressions of individual users over long time periods, we are potentially able to infer details of happiness dynamics such as individual stability, social correlation and contagion [28], and connections to well-being and health [19], [22], [27].

We further focus our present work on our essential findings regarding temporal variations in happiness including: the overall time series; regular cycles at the scale of days and weeks; time series for subsets of tweets containing specific keywords; and detailed comparisons between texts at the level of individual words. We also compare happiness levels with measures of information content, which we show are, in general, uncorrelated quantities (see 7.2). For information, as we explain below, we employ an estimate of lexical size (or effective vocabulary size) which is related to species diversity for ecological populations and is derived from generalized entropy measures [29].

Our methods and findings complement a number of related efforts undertaken in recent years regarding happiness and well-being including: large-scale surveys carried out by Gallup [30]; population-level happiness measurements carried out by Facebook's internal data team [31] and others [32]; work focusing directly on sentiment detection based on Twitter [33]–[37]; and survey-based, psychological profiles as a function of location, such as for the United States [38]. Our work also naturally builds on and shows consistency with earlier work on blogs [23], [39]–[44], which in recent years have subsided due the ascent of Twitter and other services such as Facebook.

We structure our paper as follows: in Sec. 1, we describe our data set; in Secs. 2 and 3, we detail our methods for measuring happiness and information content, demonstrating in particular the robustness of our hedonometer while uncovering some intriguing aspects of the English language's emotional content; in Sec. 4, we present and discuss the overall time series for happiness and information; in Secs. 5 and 6, we examine the average weekly and daily cycles in detail; in Sec. 7, we explore happiness and information time series for tweets containing keywords and short phrases; and in Sec. 8, we offer some concluding remarks.

### 1 Description of data set

Since its inception, Twitter has provided various kinds of dedicated data feeds for research purposes. For the results we present here, we collected tweets over a three year period running from September 9, 2008 to September 18, 2011. To the nearest million, our data set comprises 46.076 billion words contained in 4.586 billion tweets posted by over 63 million individual users. Up until November 6, 2010, our collection represents approximately 8% of all tweets posted to that point in time [45]. A subsequent change in Twitter's message numbering rendered such estimates more difficult, but we can reasonably claim to have collected over 5% of all tweets.

Our rate of gathering tweets was not constant over time, with regions of stability connected by short periods of considerable fluctuations (shown later in detail). These changes were due to periodic alterations in Twitter's feed mechanism as the company adjusted to increasing demand on their service [46]. Twitter's tremendous growth in usage and importance over this time frame lead to several service outages, and generated considerable technical issues for us in handling and storing tweets. Nevertheless, we were able to amass a very large data set, particularly so for one in the realm of social phenomena. By August 31, 2011, we were receiving roughly 20 million tweets per day (approximately 14,000 per minute), and there were only a few days for which we did not record any data.

Each tweet delivered by Twitter was accompanied by a basic set of informational attributes; we list the salient ones in Table 1, and summarize them briefly here. First, for all tweets, we have a time stamp referring to a single world clock running on US Eastern Standard Time; and from May 21, 2009 onwards, we also have local time. Due to the importance of correcting for local time, we focus much of our analysis on the time period running from May 21, 2009 to December 31, 2010, where we chose the end date as a clean stop point.

**Table 1:** List of key informational attributes accompanying each tweet.

Tweet attributes:
Tweet text
Unique tweet ID
Date and time tweet was posted 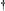
UTC offset (from GMT)
User's location
User ID
Date and time user's account was created
User's current follower count
User's current friends count
User's total number of tweets
In-reply-to tweet ID 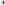
In-reply-to user ID 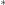
Retweet (Y/N)

Information regarding the time of posting was altered (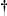) on May 21, 2009 so that local time rather than Greenwich Mean Time (GMT) was reported. If a tweet is a reply to a previous tweet, the attributes also include those indicated by an asterisk: the ID of the specific tweet's and user's ID. Twitter initially issued tweets in XML format before moving to the JSON standard [46].

User location is available for some tweets in the form of either current latitude and longitude, as reported for example by a smartphone, or a static, free text entry of a home city along with state and country. For measures of social interactions, we have a user's current follower and friend counts (but no information on who the followers and friends are), and if a tweet is made in reply to another tweet, we also have the identifying number (ID) of the latter. Finally, a ‘retweet’ flag (‘RT’) indicates if a tweet is a rebroadcasting of another tweet, encoding an important kind of information spreading in the Twitter network.

Against the many benefits of using a data source such as Twitter, there are a number of reasonable concerns to be raised, notably representativeness. First, in terms of basic sampling, tweets allocated to data feeds by Twitter were effectively chosen at random from all tweets. Our observation of this apparent absence of bias in no way dismisses the far stronger issue that the full collection of tweets is a non-uniform subsampling of all utterances made by a non-representative subpopulation of all people [47], [48]. While the demographic profile of individual Twitter users does not match that of, say, the United States, where the majority of users currently reside [49], our interest is in finding suggestions of universal patterns. Moreover, we note that like many other social networking services, Twitter accommodates organizations as users, particularly news services. Twitter's user population is therefore a blend of individuals, groups of individuals, organizations, media outlets, and automated services such as bots [50], representing a kind of disaggregated, crowd-sourced media [51]. Thus, rather than analysing signals from a few news outlets, which in theory represent and reflect the opinions and experiences of many, we now have access to signals coming directly from a vast number of individuals. Moreover, in our treatment, tweets from, say, the New York Times or the White House are given equal weight to those of any person-on-the-street.

In sum, we see two main arguments for pursuing the massive data stream of Twitter: (1) the potential for describing universal human patterns, whether they be emotional, social, or otherwise; and (2) the current and growing importance of Twitter [52] (surprising as that may be to critics of social media).

**Figure 1:**
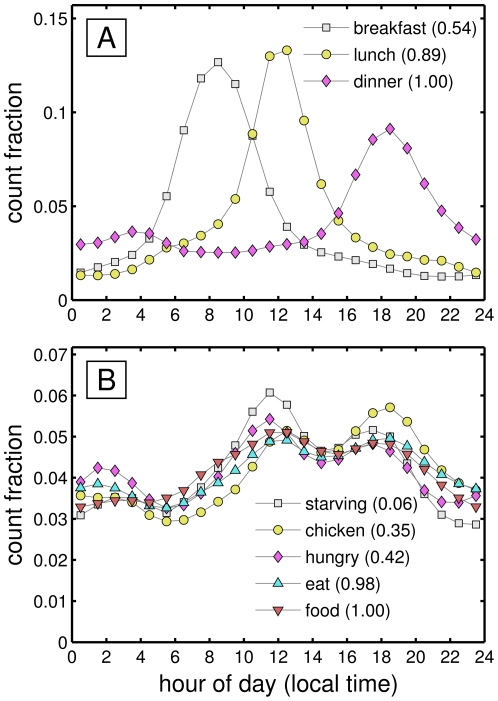
Daily trends for example sets of commonplace words appearing in tweets. For purposes of comparison, each curve is normalized so that the count fraction represents the fraction of times a word is mentioned in a given hour relative to a day. The numbers in parentheses indicate the relative overall abundance normalized for each set of words by the most common word. Data for these plots is drawn from approximately 26.5 billion words collected from May 21, 2009 to December 31, 2010 inclusive, with the time of day adjusted to local time by Twitter from the former date onwards. The words ‘food’ and ‘dinner’ appeared a total of 2,994,745 (0.011%) and 4,486,379 (0.016%) times respectively.

A preliminary glance at the data set shows that the raw word content of tweets does appear to reflect people's current circumstances. For example, Fig. 1 shows normalized daily frequencies for two food-based sets of words, binned by hour of the day. Fig. 1A shows that, as we would expect, the words ‘breakfast’, ‘lunch’, and ‘dinner’ respectively peak during the hours 8–9 am, 12–1 pm, and 6–7 pm. In Fig. 1B, we observe that the words ‘starving’, ‘chicken’ ‘hungry’, ‘eat’, and ‘food’, all follow a similar cycle with three relative peaks, one around midday, a smaller one before dinner, and another in the early morning. These trends suggest more generally that words that are correlated conceptually will be similarly congruent in their temporal patterns in tweets. Other quotidian words follow equally reasonable trends: the word ‘sunrise’ peaks between 6 and 7 am, while ‘sunset’ is most prominent around 6 pm; and the daily high for ‘coffee’ occurs between 8 and 9 am. Regular cultural events also leave their imprint with two examples from television being ‘lost’ (for the show ‘Lost’) and ‘idol’ (for ‘American Idol’) both sharply maximizing around their airing times in the evening. Further evidence that everyday people are behind a large fraction of tweets can be found in the prevalence of colloquial terms (e.g., ‘haha’, ‘hahaha’) and profanities, which we will return to later. Recent surveys also show that approximately half of Twitter users engage with the service via mobile phones [49], suggesting that individuals are often contributing tweets from their current location. Thus, while not statistically exhaustive, we have reassuring, commonsensical support for the in-the-moment nature of tweets, and we move on to our main descriptive focus: temporal patterns of societal happiness.

### 2 A robust method for measuring emotional content

#### 2.1 Algorithm for Hedonometer

We use a simple, fast method for measuring the happiness of texts that hinges on two key components: (1) human evaluations of the happiness of a set of individual words, and (2) a naive algorithm for scaling up from individual words to texts. We substantially improve here on the method introduced by two of the present authors in [23] by incorporating a tenfold larger word set for which we have obtained happiness evaluations using Mechanical Turk [24]. As we demonstrate our, hedonometer exhibits an impressive level of instrument robustness and a surprising property of tunability, similar in nature to a physical instrument such as a microscope. For the algorithm, which is unchanged from [23], we first use a pattern-matching script to extract the frequency of individual words in a given text 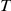. We then compute the weighted average level of happiness for the text as
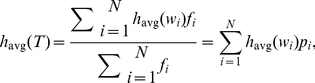
(1)where 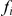 is the frequency of the 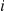
th word 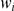 for which we have an estimate of average happiness, 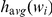, and 
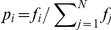
 is the corresponding normalized frequency.

For a single text, we would naturally rank the 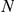 unique words found in 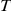 by decreasing frequency. However, in wanting to rapidly compare in detail (e.g., at the level of individual words) many pairs of massive texts assembled on the fly (e.g., by finding all tweets that contain a particular keyword), it is useful to maintain a fixed, ordered list of words. To do so, we took the most frequent 50,000 words from a large part of the overall Twitter corpus (see Methods), as a standardized list, and using this list, we then transformed texts into vectors of word frequencies. The number 50,000 was chosen both for computational ease–a master list of all words appearing in our corpus would be too large–and the fact that various measures of information content (described below) can be reliably computed.

#### 2.2 Word evaluations using Mechanical Turk

For human evaluations of happiness, we used Amazon's Mechanical Turk [24] to obtain ratings for individual words. There are three main aspects to explain here: (1) how we created our initial word list, (2) the ratings procedure, and (3) how a requirement of robustness leads us to using a tunable subset of words. As per our introductory remarks, we will refer to this data set as labMT 1.0 (Data Set S1). We discuss the first two points in this section and the third in the ensuing one.

We drew on four disparate text sources: Twitter, Google Books (English) [12], [13], music lyrics (1960 to 2007) [23], and the New York Times (1987 to 2007) [53]. For each corpus, we compiled word lists ordered by decreasing frequency of occurrence 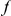, which is well known to follow a power-law decay as a function of word rank 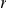 for natural texts [54]. We merged the top 5,000 words from each source, resulting in a composite set of 10,222 unique words.

By simply employing frequency as the measure of a word's importance, we naturally achieve a number of goals: (1) Precision: we have evaluations for as many words in a text as possible, given cost restrictions (the number of unique ‘words’ being tens of millions); (2) Relevance: we tailor our instrument to our focus of study; and (3) Impartiality: we do not a priori decide if a given word has emotional or meaningful content. Our word set consequently involves multiple languages, all parts of speech, plurals, conjugations of verbs, slang, abbreviations, and emotionless, or neutral, words such as ‘the’ and ‘of’.

For the evaluations, we asked users on Mechanical Turk to rate how a given word made them feel on a nine point integer scale, obtaining 50 independent evaluations per word. We broke the overall assignment into 100 smaller tasks of rating approximately 100 randomly assigned words at a time. We emphasized the scores 1, 3, 5, 7, and 9 by stylized faces, representing a sad to happy spectrum. Such five point scales are in widespread use on the web today (e.g., Amazon) and would likely be familiar with users. The four intermediate scores of 2, 4, 6, 8 allowed for fine tuning of assessments. In using this scheme, we remained consistent with the 1999 Affective Norms for English Words (ANEW) study by Bradley and Lang [55], the results of which we used in constructing our initial metric [23].

Some illustrative examples of average happiness we obtained for individual words are:
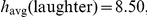


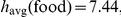


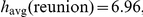


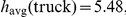


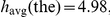


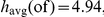


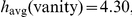


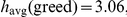


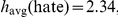


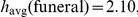


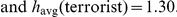
As this small sample indicates, we find the evaluations are sensible with neutral words averaging around 5.

Note that in analysing texts, we avoid stemming words, i.e., conflating inflected words with their root form, such as all conjugations of a specific verb. For verbs in particular, by focusing on the most frequent words, we obtained scores for those conjugations likely to appear in texts, obviating any need for stemming. Moreover, while we observe stemming works well in some cases for happiness measures, e.g., 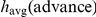 = 6.58, 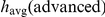 = 6.58, and 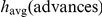 = 6.24, it fails badly in others, e.g., 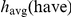 = 5.82 and 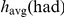 = 4.74; 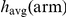 = 5.50 and 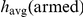 = 3.84; and 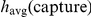 = 4.18 and 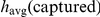 = 3.22.

In the Supplementary Information, we provide happiness averages and standard deviations for all 10,222 words, along with other information.

An immediate and reassuring sign of the robustness of the word happiness scores we obtained via Mechanical Turk is that our results agree very well with that of the earlier ANEW study which consisted of 1034 words [55] (Spearman's correlation coefficient 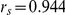 and 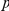-value 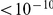). This adds to earlier suggestions of universality in the form of a high correlation between the ANEW study happiness scores and those made by participants in Madrid for a direct Spanish translation of the ANEW study words [56]. Furthermore, the ANEW study involved students at the University of Florida, a group evidently distinct from users on Mechanical Turk.

The ANEW study words were also broadly chosen for their emotional and meaningful import rather than usage frequency, and we show below that our larger frequency-based word set affords a much greater coverage of texts. (By coverage, we mean the percentage of words in a text for which we have individual happiness estimates.) Note that in the ANEW study and our earlier work [23], happiness was referred to as psychological valence, or simply valence, a standard terminology [57].

#### 2.3 Robustness and Refinement of Hedonometer

We now show that our hedonometer can be improved by considering the effects of taking subsets of the overall list of 10,222 words. Clearly, truly neutral words such as ‘the’ and ‘of’ should be omitted, especially because of their high relative abundance, thereby forming a list of excluded words commonly referred to as stop words [58].

Because we have filtered by frequency in selecting our word list, we are able to determine stop word lists in a principled way, leading to a feature of tunability. Here, we exclude words whose average happiness 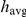 lies within 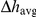 of the neutral score of 5, i.e., 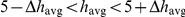. In other words, we remove all words lying in a centered band of width 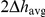 on our happiness spectrum.

**Figure 2:**
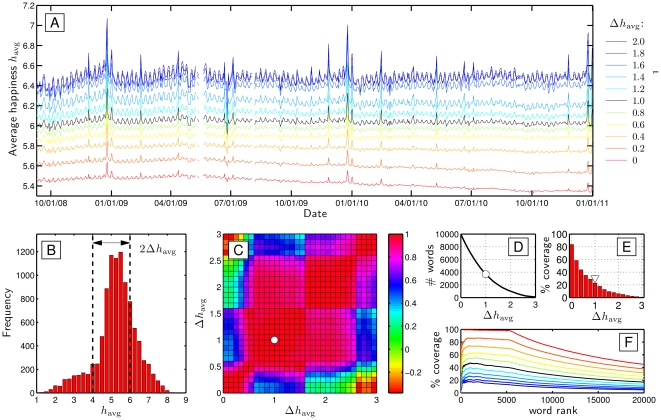
Demonstration of robustness and tunability of our text-based hedonometer, and reasoning for choice of a specific metric. To measure the happiness of a given text, we first compute frequencies of all words; we then create an overall happiness score, Eq. (1) , as a weighted average of subsets of 10,222 individual word happiness assessments on a 1 to 9 scale, obtained through Mechanical Turk (see main text and Methods). In varying word sets by excluding stop words [58], we can systematically explore families of happiness metrics. In plot **A**, we show time series of average happiness for Twitter, binned by day, produced by different metrics. Each time series is generated by omitting words with 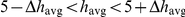 as indicated in plot **B**, which shows the overall distribution of average happiness of individual words. For 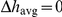 we use all words; as 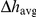 increases, we progressively remove words centered around the neutral evaluation of 5. Plot **C** provides a test for robustness through a pairwise comparison of all time series using Pearson's correlation coefficient. For 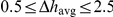, the time series show very strong mutual agreement. We choose 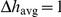 (black curve in **A** and **F**, shown in **B**, white symbols in **C**, **D**, and **E**) for the present paper because of its excellent correlation in output with that of a wide range of 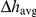, and for reasons concerning the following trade-offs. In **A**, we see that as the number of stop words increases, so does the variability of the time series, suggesting an improvement in instrument sensitivity. However, at the same time, we lose coverage of texts. Plot **D** first shows how the number of individual words for which we have evaluations decreases as 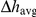 increases. For 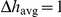, we have 3,686 individual words down from 10,222. Plot **E** next shows the percentage of the Twitter data set covered by each word list, accounting for word frequency; for 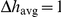, our metric uses 22.7% of all words. Lastly, in plot **F** (which uses plot **A**'s legend), we show how coverage of words decreases with word rank. When 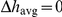, we incorporate all low rank words, with a decline beginning at rank 5,000. For 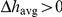, we see similar patterns with the maximum coverage declining; for 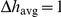, we see a maximum coverage of approximately 50%.

We explore and demonstrate our hedonometer's behavior [Eq. (1)] with respect to different stop word lists by varying 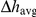, with our main results and evidence displayed in the six panels of Fig. 2. We will argue in particular that 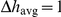 yields a robust, sensitive, and informative hedonometer, and this will be our choice for the remainder of the paper. However, a range of values of 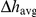 will also prove to be valid, meaning that 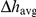 is a tunable parameter.

As a test case and as shown in Fig. 2A, we focus on measuring the happiness time series for Twitter running from September 9, 2008 to December 31, 2010, resolved at the level of days, and for 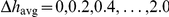. (Once we explain our selection of 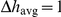, black curve in 2A, we will return in the next section to study the overall time series in detail.) In Fig. 2B, we show a histogram of average happiness levels for all 10,222 words, indicating the stop word selection for 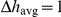. Several features are apparent: (1) the time series are broadly similar to the eye; (2) as we expand the stop word list, the base line level of happiness and size of fluctuations both increase; (3) an overall downward trend apparent for small 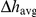 becomes less pronounced as 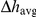 increases; and (4) English words, as they appear in natural language, are biased toward positivity, a phenomenon we explore elsewhere [59]. Note that point (4) explains point (2): the increasing relative abundance of positive words leads to an inflation of overall happiness as 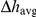 increases.

We quantify the similarity between time series by computing Pearson's correlation coefficient for each pair of time series with 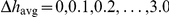. In Fig. 2C, we observe an impressively high correlation for all pairs of time series with 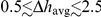, forming the central large square (the white circle corresponds to 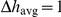). For the range 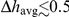, the resultant time series are internally consistent but a clear break occurs with time series for 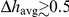. This transition appears to be due in part to the relative increase of languages other than English on Twitter since mid 2009, which we discuss later in Sec. 4.3.

The striking congruence for all time series generated with 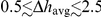 suggests that we may use 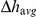 as a tuning parameter, a remarkable consequence of the emotional structure of the English language. Larger values of 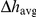 (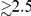) give us a higher resolution or sensitivity (the time series fluctuate more) but at a loss of overall word coverage leading to a more brittle instrument. This effect is reminiscent of increasing the contrast in an image, or edge detection. More generally, we could choose any range of word happiness as a ‘lens’ into a text's emotional content. For example, we could take words with 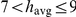 to highlight the positive elements of a text. Thus, as a practical instrument implemented online, we would recommend the inclusion of 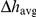 as a natural tuning parameter.

For the purposes of this paper, it is most useful if we choose a specific value of 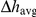 in this range. As we have indicated, we find 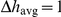 to be a suitable compromise in balancing sensitivity versus robustness, i.e., the ability to pick up variations across texts (requiring higher 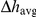) versus text coverage (requiring lower 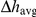). In choosing 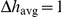, we are also safely above the transitional value of 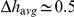.

We support the robustness of our choice with evidence provided in Figs. 2D, 2E, and 2F, which together show how word coverage declines with increasing 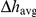. In Fig. 2D, we plot the number of unique words left in our labMT 1.0 word list (Data Set S1) as a function of 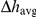. For 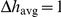, 3,686 unique words of the original 10,222 remain. The fraction of the Twitter corpus covered by these 3,686 word is approximately 23% (Fig. 2E). By comparison, the ANEW study's 1,034 words collectively cover only 3.7% of the corpus, typical of other texts we have analysed such as blogs, books, and State of the Union Addresses [23]. This discrepancy in total coverage is again due to the ANEW word list's origin being more to do with meaning than frequency.

Fig. 2F shows how our coverage of words in the Twitter corpus decays as a function of frequency rank 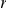. For 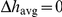, our coverage is complete out to 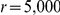 where we begin to miss words. The same basic curve is apparent for 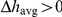, with a clear initial dip due to the exclusion of common neutral words. For 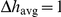, we cover between 40 to 50% for 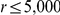.

As a final testament to the quality of our hedonometer, we note that in an earlier version of the present paper [60], and prior to completing our word evaluation survey using Mechanical Turk, we used the ANEW study word list in all our analyses; the interested reader will be able to make many direct comparisons of figures and tables. Broadly speaking, we find the same trends with our improved word set, again speaking to the robustness of our instrument and indeed the English language. In the manner of a true measuring instrument, we obtain much greater resolution and fidelity with the labMT 1.0 word list (Data Set S1), sharpening observations we made using the ANEW study, and bringing new ones to light that were previously hidden.

#### 2.4 Limitations

We address several key aspects and limitations of our measurement. First, as with any sentiment analysis technique, our instrument is fallible for smaller texts, especially at the scale of a typical sentence, where ambiguity may render even human readers unable to judge meaning or tone [61]. Nevertheless, problems with small texts are not our concern, as our interest here is in dealing with and benefiting from very large data sets.

Second, we are also effectively extracting a happiness level as perceived by a generic reader who sees only word frequency. Indeed, our method is purposefully more simplistic than traditional natural language processing (NLP) algorithms which attempt to infer meaning (e.g., OpinionFinder [62], [63]) but suffer from a degree of inscrutability. By ignoring the structure of a text, we are of course omitting a great deal of content; nevertheless, we have shown using bootstrap-like approaches that our method is sufficiently robust as to be meaningful for large enough texts [23].

Third, we quantify only how people appear to others; as should be obvious, our method cannot divine the internal emotional states of specific individuals or populations. In attempting to truly understand a social system's potential dynamical evolution, we would have to account for publicly hidden but accessible internal ranges and states of emotions, beliefs, etc. However, a person's exhibited emotional tone, now increasingly filtered through the signal-limiting medium of written interactions (e.g., status updates, emails, and text messages), is that which other people evidently observe and react to.

Last, by using a simple kind of text analysis, we are able to non-invasively, remotely sense the exhibited happiness of very large numbers of people via their written, open, web-scale output. Crucially, we do not ask people how happy they are, we merely observe how they behave online. As such, we avoid the many difficulties associated with self-report [64]–[66]. We refer the reader to our initial work for more discussion of our measurement technique [23].

### 3 Measuring word diversity

In quantifying a text's information content, we use concepts traditionally employed for estimating species diversity in ecological studies [29] which build on information theoretic approaches. As we outline below, direct measures of information can be transformed into estimates of lexical size (or word diversity), with the benefit that comparisons of the latter are more readily interpretable.

A first observation is that the sheer number of distinct words in a text is not a good representation of lexical size. Because natural texts generally exhibit highly skewed distributions of word frequencies, such a measure discards much salient information, and moreover is difficult to estimate if a text is subsampled.

To arrive at a more useful and meaningful quantity, we consider generalized entropy: 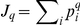 where, for a given text, 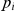 is the 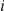
th distinct word's normalized frequency of occurrence and which we interpret as a probability. In varying the parameter 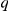, we tune the relative importance of common versus rare words, with large 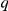 favoring common ones.

These generalized entropies can be seen as direct measures of information but their values can be hard to immediately interpret. To make comparisons between the information content of texts more understandable, if by adding an extra step, we use these information measures to compute an equivalent lexical size, 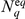, which is the number of words that would yield the same information measure if all words appeared with equal frequency [29].

We observe that the lexical sizes 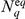 for 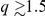 closely follow the same trends for the data we analyse here. In therefore needing to show only one representative measure among the 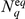, we choose 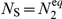 based on Simpson's concentration 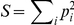, corresponding to generalized entropy with 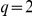 [67]. A simple calculation gives 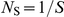. Simpson's concentration can be seen as the probability that any two words chosen at random will be the same. Simpson's concentration is also related to the Gini coefficient 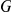, which is often used to characterize income inequality, as 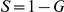. For text analysis, 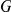 represents the probability that two randomly chosen words are different.

Using 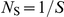 for lexical size holds several theoretical and practical benefits: (1) 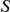 has the natural probablistic interpretation given above; (2) The quantity 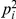 decays sufficiently rapidly that we need not be concerned about subsampling heavy tailed distributions (see Methods); and (3) In comparing two texts, the contributions to 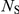 due to changes in individual word frequencies combine linearly and thus can be easily ranked. From here on, we will focus on 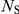 which we will refer to as a text's ‘Simpson lexical size.’

## Results and Discussion

### 4 Overall time dynamics of happiness and information

**Figure 3:**
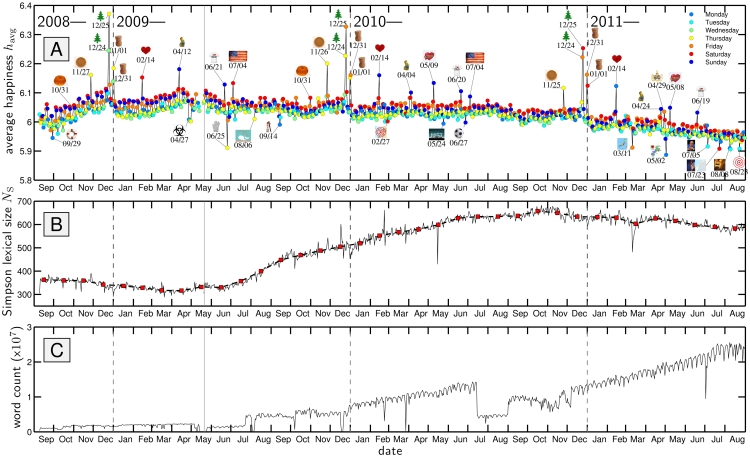
Overall happiness, information, and count time series for all tweets averaged by individual day. **A.** Average happiness measured over a three year period running from September 9, 2008 to August 31, 2011 (see Sec. 3 for measurement explanation). A regular weekly cycle is clear with the red and blue of Saturday and Sunday typically the high points (examined further in Fig. 5). Post May 21, 2009 (indicated by a solid vertical line), we use reported local time to assign tweets to particular dates. See also Figs. S1 and S2. **B.** Simpson lexical size 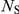 as a function of date using Simpson's concentration as the base entropy measure (solid gray line; see Sec. 3). The red squares with the dashed line show 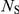 as a function of calendar month. **C.** The number of words extracted from all tweets as a function of date for which we used evaluations from Mechanical Turk. For both the happiness and Simpson lexical size plots, we omit dates for which we have less than 1000 words with evaluations.

We observe a variety of temporal trends in happiness and information content across timescales of hours, days, months, and years. In Fig. 3A we present the average happiness time series with tweets binned by day. The accompanying plots, Figs. 3B and 3C, show the Simpson lexical size 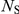, discussed in Sec. 4.3, and the number of words for which we have evaluations from Mechanical Turk (using 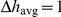). We expect such a coarse-grained averaging to leave only truly system wide signals, and as we show later in Section 7, subsets of tweets exhibit markedly different temporal trends. In the Supplementary Information, we provide a zoomable, high resolution version, Fig. S1, as well as simpler plots of the time series only, Fig. S2.

Looking at the complete time series, we see that after a gradual upward trend that ran from January to April, 2009, the overall time series has shown a gradual downward trend, accelerating somewhat over the first half of 2011. We also see that average happiness gradually increased over the last months of 2008, 2009, and 2010, and dropped in January of the ensuing years. Moving down to timescales less than a month, we see a clear weekly signal with the peak generally occurring over the weekend, and the nadir on Monday and Tuesday (c.f., [23], [32], [40], [42], [68]). We return to and examine the weekly cycle in detail in Sec. 5.

#### 4.1 Outlier Dates

At the scale of a day, we find a number of dates which strongly deviate in their happiness levels from nearby dates, and we indicate these in Fig. 3A. We discuss positive and negative dates separately, noting that anomalously positive days occur mainly on annual religious, cultural, and national events, whereas negative days typically arise from unexpected societal trauma due for example to a natural disaster or death of a celebrity. (See [39] for similar, earlier work on blogs.)

In the following section, we look more closely at several dates, showing how individual words contribute to their anomalous measurements.

For the outlying happy dates, in 2008, 2009, and 2010, Christmas Day returned the highest levels of happiness, followed by Christmas Eve. Other relatively positive dates include New Year's Eve and Day, Valentine's Day, Thanksgiving, Fourth of July, Easter Sunday, Mother's Day, and Father's Day. All of these observations are sensible, and reflect a strong (though not universal) degree of social synchrony. The spikes for Thanksgiving and Fourth of July reflects the fact that while Twitter is a global service, the majority of users still come from the United States [48]. The only singular, non-annual event to stand out as a positive day was that of the Royal Wedding of Prince William and Catherine Middleton, April 29, 2011.

Over the entire time span, we see substantial, system-wide drops in happiness in response to a range of disparate events, both exogenous and endogenous in nature. Working from the start of our time series, we first see the Bailout of the U.S. financial system, which induced a multi-week depression in our time series. The lowest point corresponds to Monday, September 29, 2008, when the U.S. government agreed to an unprecedented purchase of toxic assets in the form of mortgage backed securities.

Following the 2008 Bailout, we see the overall time series rebound well through the end of 2008, suffer the usual post New Year's dip, and begin to rise again until an extraordinary week long drop due to the onset of the 2009 swine flu or H1N1 pandemic.

The next decline occurred with Michael Jackson's death, the largest single day drop we observed. His memorial on July 7, 2009 induced another clear negative signal. The death of actor Patrick Swayze on September 14, 2009 also left a discernible negative impact on the time series. In between, Twitter itself was the victim of a large-scale distributed denial of service attack, leading to an outage of the service; upon resumption, tweets were noticeably focused on this internal story.

Several natural disasters registered as days with relatively low happiness: the February, 2010 Chilean earthquake, the October, 2010 record size storm complex across the U.S., and the March, 2011 earthquake and tsunami which devastated Japan.

Reports of the killing of Osama Bin Laden on May 2, 2011 resulted in the day of the lowest happiness across the entire time frame. And global sport left one identifiable drop: the 4–1 victory of Germany over England in the 2010 Football World Cup. Spain's ultimate victory in the tournament was detectable in terms of word usage but did not lead to a significant change in overall happiness.

One arguably false finding of a cultural event being negative was the finale of the last season of the highly rated television show ‘Lost’, marked by a drop in our time series on May 24, 2010, and in part due to the word ‘lost’ having a low happiness score of 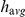 = 2.76, but also to an overall increase in negative words on that date.

A number of these departures for specific dates qualitatively match observations we made in our earlier work on blogs [23], though we make any comparison tentatively as for blogs we focused on sentences written in the first person containing a conjugation of the verb ‘to feel’ [69]. For example, Christmas Eve and Day, New Year's Eve and Day, and Valentine's Day all exhibit jumps in happiness in both tweets and ‘I feel…’ blog sentences. Both time series also show a pronounced drop for Michael Jackson's death. However, tweets did not register a similar lift as blogs for the US Presidential Election in 2008 and Inauguration Day, 2009, while positive sentiment for both Mother's and Father's Day, the Fourth of July, are much more evident in tweets. Lastly, blogs typically showed drops for September 10 and/or 11 that are largely absent in tweets, although relevant negative words appear more frequently on those dates (e.g., ‘lost’, ‘victims’, and ‘tragedy’).

#### 4.2 Word Shift Analysis

When comparing two or more texts using a single summary statistic, as we have here with average happiness, we naturally need to look further into why a given measure shows variation. In Fig. 4 we provide ‘word shift graphs’ for three outlier days relative to the seven preceding and seven ensuing days combined: the 2008 Bailout of the U.S. financial system, the 2011 Royal Wedding, and Osama Bin Laden's death (we include corresponding graphs for all identified outlier days in Figs. S7, S8, S9, S10, S11, S12, S13, S14, S15, S16, S17, S18, S19, S20, S21, S22, S23, S24, S25, S26, S27, S28, S29, S30, S31, S32, S33, S34, S35, S36, S37, S38, S39, S40, S41, S42, S43, S44, S45, S46, S47, S48, S49, S50, S51, S52). We will use these word shift graphs, which we introduced in [23] and improve upon here, throughout the remainder of the paper to illuminate how the difference between two texts' happiness levels arises from changes in underlying word frequency. In view of the utility of these graphs, we take time now to describe and explain them in detail.

**Figure 4:**
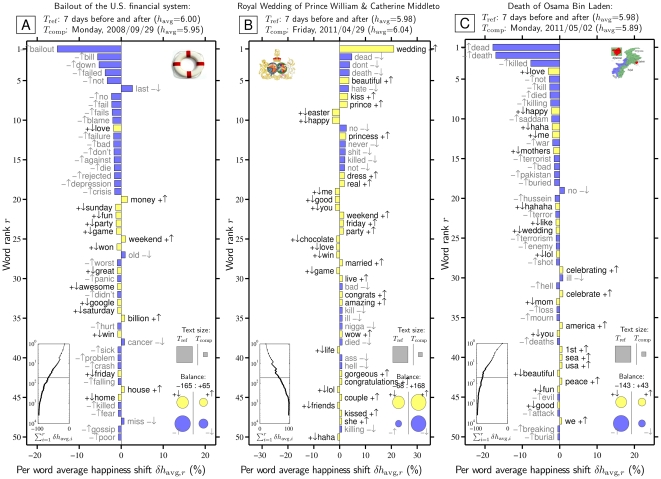
Word shift graph showing how changes in word frequencies produce spikes or dips in happiness for three example dates, relative to the 7 days before and 7 days after each date. Words are ranked by their percentage contribution to the change in average happiness, 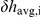. The background 14 days are set as the reference text (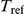) and the individual dates as the comparison text (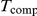). How individual words contribute to the shift is indicated by a pairing of two symbols: 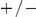 shows the word is more/less happy than 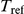 as a whole, and 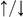 shows that the word is more/less relatively prevalent in 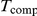 than in 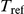. Black and gray font additionally encode the 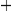 and 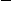 distinction respectively. The left inset panel shows how the ranked 3,686 labMT 1.0 words (Data Set S1) combine in sum (word rank 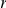 is shown on a log scale). The four circles in the bottom right show the total contribution of the four kinds of words (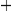 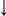, 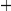 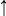, 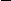 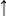, 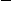 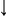). Relative text size is indicated by the areas of the gray squares. See Eqs. 2 and 3 and Sec. 4.2 for complete details.

Consider two texts 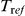 (for reference) and 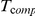 (for comparison) with happiness scores 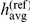 and 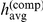. If we wish to compare 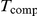 relative to 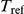 then, using Eq. (1) , we can write
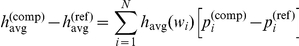


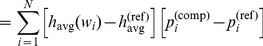
(2)where we have employed the fact that
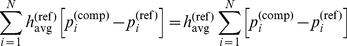


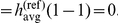
In introducing the term 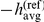, we are now able to make clear the contribution of the 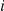
th word to the difference 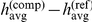. From the form of Eq. (2) , we see that we need to consider two aspects in determining the sign of the 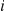
th word's contribution:

1. Whether or not the 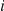
th word is on average happier than text 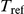
's average, 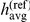; and
2. Whether or not the 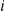
th word is relatively more abundant in text 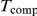 than in text 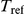.

We will signify a word's happiness relative to text 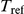 by 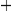 (more happy) and 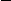 (less happy), and its relative abundance in text 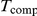 versus text 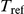 with 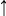 (more prevalent) and 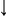 (less prevalent). Combining these two binary possibilities leads to four cases:

+↑: Increased usage of relatively positive words–If a word is happier than text 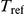 (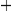) and appears relatively more often in text 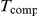 (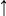), then the contribution to the difference 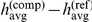 is positive;

−↓: Decreased usage of relatively negative words–If a word is less happy than text 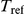 (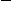) and appears relatively less often in text 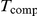 (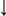), then the contribution to the difference 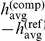 is also positive;

+↑: Decreased usage of relatively positive words–If a word is happier than text 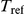 (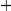) and appears relatively less often in text 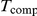 (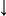), then the contribution to the difference 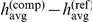 is negative; and

+↓: Increased usage of relatively negative words–If a word is less happy than text 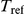 (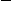) and appears relatively more often in text 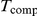 (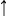), then the contribution to the difference 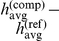 is also negative.

For the convenience of visualization, we normalize the summands in Eq. (2) and convert to percentages to obtain:
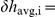


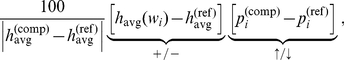
(3)where 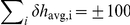, depending on the sign of the difference in happiness between the two texts, 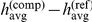, and where we have indicated the terms to which the symbols 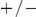 and 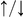 apply. We call 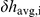 the per word happiness shift of the 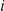
th word.

Finally, in comparing two texts, we rank words by their absolute contribution to the change in average happiness, 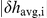, from largest to smallest. In doing so, we are able to make clear the most important words driving the separation of two texts' emotional content.

With these definitions in hand, we return to Fig. 4 to complete our explanation of word shift graphs. For brevity we will refer to these graphs with the terms Bailout, Royal Wedding, and Bin Laden.

The primary element of our word shift graphs is a central bar graph showing a desired number of highest ranked labMT 1.0 words (Data Set S1) as ordered by their absolute contribution to the change in average happiness, 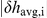. In Fig. 4, the word shift graphs show the first 50 words for each date. Bars corresponding to words that are more happy than the reference text 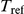 are colored yellow, and less happy ones are colored blue.

In each graph in Fig. 4, we see examples of each of the four ways words can contribute to 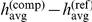. For the Bailout, both kinds of negative changes dominate with 42 of the top 50 shifts, including more of the relatively negative words ‘bailout’, ‘bill’, ‘down’, ‘no’, ‘not’, ‘fail’, ‘blame’, and ‘panic’ (all 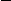 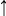), and less of the relatively positive words ‘fun’, ‘party’, ‘game’, ‘awesome’, and ‘home’ (all 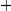 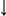). For the Bin Laden graph, 40 out of the first 50 ranked words contribute to the overall drop (bars on left). The strongest decreases come from ‘dead’ and ‘death’ and these combine with more negativity found in ‘killed’, ‘kill’, ‘died’, ‘killing’, ‘terrorist’, ‘buried’, and ‘Pakistan’ (all 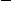 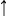).

By contrast, we see the happiness spike of the Royal Wedding is due to higher prevalence of positive words such as ‘wedding’, ‘beautiful’, ‘kiss’, ‘prince’, ‘princess’, ‘dress’, and ‘gorgeous’ (all 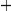 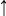), and a relative dearth of negative words such as ‘dead’, ‘death’, ‘hate’, ‘no’, ‘never’, and several profanities (all 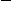 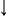).

Beyond these dominant stories, our word shifts allow us to make a number of supporting and clarifying observations. First, since we have chosen to compare specific dates to the surrounding 14 days, nearby anomalous events appear in each word shift. For example, the Royal Wedding (2011/4/29) has less ‘Easter’ and ‘chocolate’ because Easter occurred five days earlier and less ‘dead’ and ‘killed’ because of Bin Laden's death three days later (2011/5/2). The Bin Laden graph in turn shows less ‘wedding’, ‘happy’, and ‘Mother's’ (due to the Royal Wedding and Mother's Day, 2011/5/8). Other reference texts can be readily constructed for comparisons (e.g., tweets on all days or matching weekdays). However, we find that the main words contributing to word shifts reliably appear as we consider alternative, reasonable reference texts.

Second, in all text comparisons, we find some words go against the main trend. For example, we see more ‘money’, ‘weekend’, and ‘billion’ (all 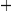 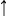), and less ‘last’ and ‘old’ (all 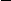 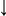) for the Bailout word shift; less ‘me’, ‘good’, and ‘haha’ for the Royal Wedding (all 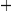 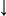); and more ‘celebrating’, ‘America’, and ‘USA’ for Bin Laden's death (all 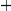 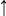). Some shifts are genuinely at odds with the overall shift (e.g., ‘celebrating’ for Bin Laden) while others appear due to our omission of context (e.g., the generally positive word ‘money’ was not being talked about in a positive way during the Bailout). In the case of the Bailout, our instrument overcomes its inherent coarseness to yield intuitive overall measurements. For Bin Laden's death, which would arguably be a positive moment for many users of Twitter, the death of a profoundly negative character results in word usage that appears, not unreasonably, as a surge of negative emotion. Every reading on our hedonometer, anomalous or not, and indeed that of any sentiment measurement, must be validated by plain demonstration of which words are most salient.

The three insets in the word shift graphs of Fig. 4 expand the story provided by the main bar charts in the following ways. First and simplest is the pair of gray squares on the right which show, by their area, the relative sizes of the two texts, as measured by the total number of labMT 1.0 words (Data Set S1) (the absolute number of words is not indicated). For these comparisons, the ratio is therefore approximately 14∶1.

Second, on the bottom left of each word shift graph, the inset line graph shows the cumulative sum of the individual word contributions, 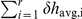 as a function of 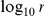 where 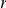 is word rank. The graph shows how rapidly the word contributions converge to 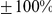 as we include all 3,686 words. The solid line marks 50 words, the number of words in the main panel. We typically see that the first 1000 words account for more than 99% of the entire shift.

The third and final inset on the bottom right is a key one. An increase in happiness may be due to the use of more positive words, an avoidance of negative words, or a combination of both, and we need to quantify this in a simple way. The inset's four circles show the relative total contributions of the four classes of words to the overall shift in average happiness. For example, the area of the top right (yellow) circle represents the sum of all contributions due to relatively positive words that increase in frequency in 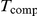 with respect to 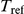 (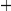 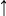). We find that the sizes of these circles are not always transparently connected to the top 50 words, with smaller contributions combining over the full set of 3,686 words.

The two numbers above the circles give the total percentage change toward and away from the reference text's average happiness. For the Bailout example, there is a drop in happiness of −165% of 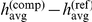 due to less use of positive words, 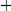 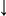, and more use of negative words, 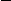 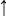. On the other side, more frequent positive words, 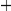 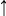, and less frequent negative words, 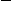 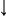, contribute to a rise in happiness equal to +65% of 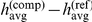. The two changes combine to give −100% of 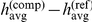.

For the Bailout and Bin Laden graphs, we see similar overall patterns: the more frequent use of negative words (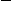 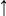) dominates while the less frequent use of positive words (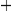 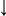) is also substantive; and we see the smaller countering effects of the other two classes of words are about equal (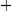 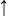 and 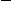 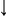). For the Royal Wedding, the relative increase in happiness of the day is equally due to more frequent use of positive words and less frequent use of negative words (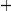 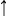 and 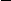 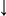), while very few negative words are more prevalent (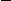 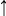).

#### 4.3 Information Content

To complete our analysis of the overall time series, we turn to information content (Fig. 3B). We see a strong increase in Simpson lexical size 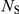 climbing from approximately 300 to 700 words beginning around July, 2009. (For 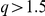, generalized word diversities all follow the same trajectory with 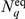 increasing as 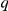 decreases.) We also indicate in the same plot 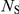 measured at the scale of months (red squares). The smoothness of the resulting curve shows that 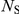 is unaffected by the two issues of missing data and non-uniform sampling rates. (Note that the month estimates of 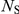 are computed from the word distribution for the month and are not simply averages of daily values of 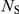.)

By examining shifts in word usage, we are able to attribute the more than doubling of 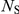 to a strong relative increase in non-English languages, notwithstanding the dramatic growth in English language tweets. Recalling that the most common words such as articles and prepositions figure most strongly in the computation of the Simpson word diversity, we see the dominant growth in Spanish (‘que’, ‘la’, ‘y’,’en’, ‘el’). A few other example languages making headway are Portuguese (‘pra’), which also shares some common words with Spanish, and Indonesian (‘yg’). By contrast, English words appear relatively less (including the word ‘Twitter’) while a minority of words move against the general diversification by appearing more frequently, with prominent examples being the abbreviations ‘RT’ (for retweet) and ‘lol’ (for laugh out loud).

### 5 Weekly cycle

#### 5.1 Average Happiness of Weekdays

**Figure 5:**
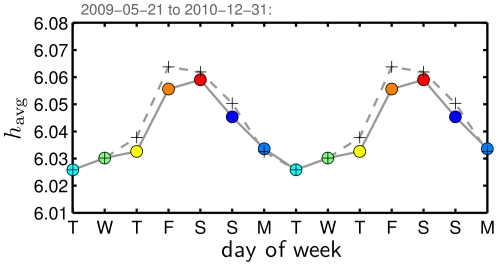
Average happiness as a function of day of the week for our complete data set. To make the average weekly cycle more clear, we repeat the pattern for a second week. The crosses indicate happiness scores based on all data, while the filled circles show the results of removing the outlier days indicated in Fig. 3A. The colors for the days of the week match those used in Fig. 3A. To circumvent the non-uniform sampling of tweets throughout time, we compute an average of averages: for example, we find the average happiness for each Monday separately, and then average over these values, thereby giving equal weight to each Monday's score. We use data from May 21, 2009 to December 31, 2010, for which we have a local timestamp.

As we saw in Fig. 3A, a pronounced weekly cycle is present in the overall time series. To reveal this feature more clearly, we compute average happiness 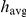 as a function of day of the week, Fig. 5. Taking tweets for which we have local time information (May 21, 2009 onward), we show two curves, one for which we include all data (crosses, dashed line), and one for which we exclude the outlier days we identified in Fig. 3A (labeled dates accompanied by icons). Including outlier days yields a higher average happiness, and the difference between the two curves is most pronounced on Thursday, Friday, Saturday, and Sunday. These discrepancies are explained by Thanksgiving (Thursday), and Christmas Eve and Day and New Year's Eve and Day falling on Thursday and Friday in 2009 and Friday and Saturday in 2010, as well as annual events such as Mother's Day occurring on Sundays.

We take the reasonable step of focusing on the data with outlier days removed. We see Saturday has the highest average happiness (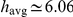), closely followed by Friday and then Sunday. From Saturday, we see a steady decline until the weekly low occurs on Tuesday, which is then followed by small increases on both Wednesday and Thursday (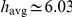). We see a jump on Friday, leading back to the peak of Saturday. Roughly similar patterns have been found in Gallup polls [30], in Facebook by the company's internal research team [31], in binary sentiment analysis of tweets [37], and in analyses of smaller collections of tweets [70]. (In the last work and in contrast to our findings here for a data set tenfold larger in size, Thursday evening was identified as the low point of the week.)

**Figure 6:**
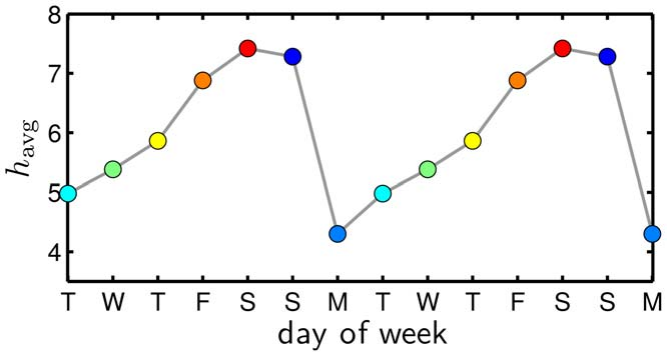
Evaluations of the individual days of the week as isolated words using Mechanical Turk.

While the weekend peak in the cycle conforms with everyday intuition, the minimum on Tuesday goes against standard notions of the Monday blues with its back-to-work nature, and Wednesday's middle-of-the-week labeling as the work week's hump day [42]. To provide a quantitative comparison, in Fig. 6, we show how people's perception of days of the week varies based on our Mechanical Turk study, i.e., how people rate the words ‘Monday’, ‘Tuesday’, etc., when presented with them in a survey. The overall pattern is similar in terms of ordering with the exception of ‘Monday’ being rated the lowest rather than ‘Tuesday’, and ‘Sunday’ is rated above ‘Friday’. The range of happiness is also much greater, 4.30 for ‘Monday’ to 7.42 for ‘Saturday’, sensibly so since we are now considering evaluations of individual words with no averaging over texts. While people collectively have strong opinions about the word ‘Monday’, the reality, at least in terms of tweets, is that Tuesday is the week's low point.

In our earlier work on blogs using the ANEW study word list [23], we saw a statistically significant but much weaker cycle for the days of the week; the high and low days were Sunday and Wednesday (see also [42]). The discrepancy appears to be due to the in-the-moment character of Twitter versus the reflective one of blogs.

**Figure 7:**
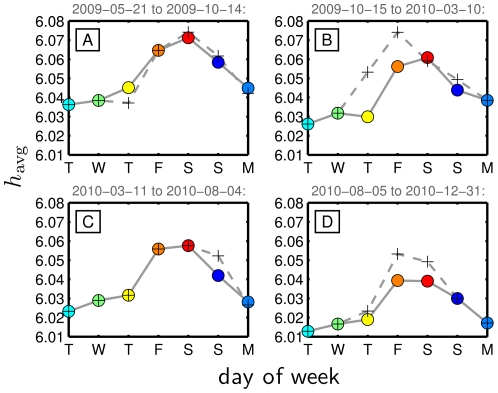
Average of daily average happiness for days of the week over four consecutive time periods of approximately five months duration each. As per Fig. 5, crosses are based on all days, circles for days excluding outlier days marked in Fig. 3. The vertical scale is the same in each plot and matches that used in Fig. 5.

With any observed pattern, a fundamental issue is universality. Is the three day midweek low followed by a peak around Saturday a pattern we always see, given enough data? Further inspection of our Twitter data set shows a constancy in the weekly cycle occurring over time. In Fig. 7, we aggregate tweets for days of the week for four time ranges, approximately equal in duration. As before, we show the weekly pattern for all days (crosses, dashed curve) and with outlier days marked in Fig. 3A removed (disks, solid curve). The major differences we observe between these two curves in the four panels are predominantly explained as before by Christmas, New Year's, and Thanksgiving. In terms of universality, we again see that Friday-Saturday-Sunday represents the peak while Tuesday's level is the minimum in each period. Only for Thursday in Fig. 7B do we see a change in the overall ordering of days. Thus, we have some confidence that the overall weekly cycle of happiness shown in Fig. 5 is a fair description of what appears to be a robust pattern of users' expressed happiness.

#### 5.2 Word Shift Analysis

**Figure 8:**
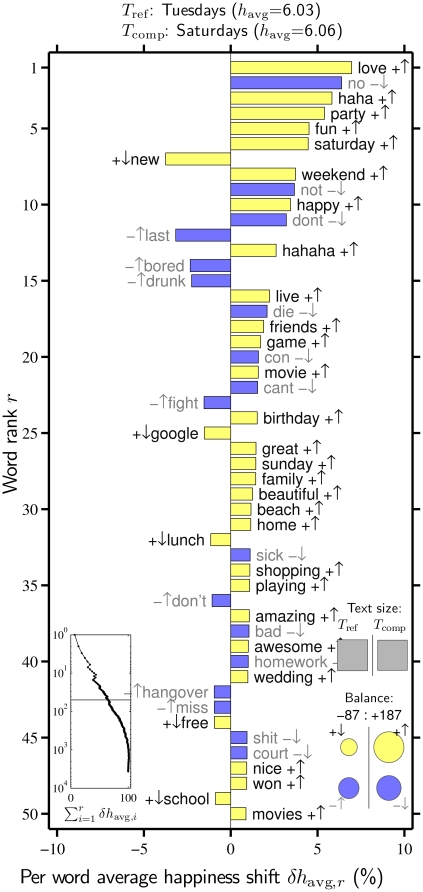
Word shift graph comparing Saturdays relative to Tuesdays. Each day of the week's word frequency distribution was generated by averaging normalized distributions for each instance of that week day in May 21, 2009 to December 31, 2010, with outlier dates removed. See Fig. S5 for word shifts based on alternate distributions.

In Fig. 8, we present a word shift graph comparing tweets made on Saturdays relative to those made on Tuesdays. We created word frequency distributions for each day by averaging normalized distributions from May 21, 2009 to December 31, 2010, removing the outlier dates marked in Fig. 5A. Alternate ways of creating the weekday distributions do not change the word shifts appreciably (See Fig. S22). The two kinds of positive changes dominate with 38 of the top 50 changes, including more of ‘love’, ‘haha’, ‘party’, ‘fun’, ‘Saturday’, ‘happy’, and ‘hahaha’ (all 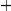 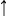), and less of ‘no’, ‘not’, ‘don't’, ‘can't’, ‘bad’, and ‘homework’ (all 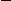 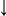). These changes are readily interpretable, with the weekend involving more leisure and family time, and a relative absence of work, school, and related concerns. Words in the top 50 which move against the general trend are the more prevalent, relatively negative words ‘last’, ‘bored’, ‘drunk’, ‘fight’, and ‘hangover’ (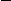 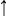), and the less frequent positive words ‘new’, ‘google’, and ‘lunch’ (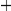 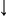). Thus while Saturdays may be on average happier than Tuesdays, we also see evidence of boredom, fighting, and suffering due to excessive drinking.

The insets of Fig. 8 provide further insight and information. The gray squares indicate the word base for Tuesdays and Saturdays are of comparable size. From the bottom left line graph, we see again that around 1000 words account for the shift in average happiness between Tuesday and Saturday, and that the first 50 words make up approximately 60% of the shift.

The bottom right inset shows that the overall positive shift from Tuesdays to Saturdays is due to the more frequent use of positive words (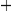 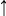), and to a lesser extent, the less frequent use of negative words (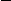 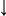). On the other side of the ledger, we see a smaller total contribution of words going against the trend of happier Saturdays, noting that the increased use of certain negative words (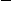 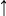) is slightly more appreciable in impact than the less frequent use of positive words (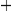 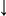).

#### 5.3 Information Content

**Figure 9:**
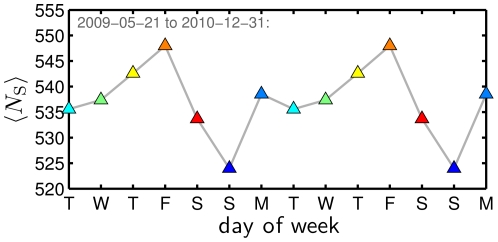
Simpson lexical size as a function of day of the week. We compute 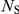 for individual dates Fig. 3B, again excluding dates shown in Fig. 3A, and then average these values. (See also Fig. S20 for the effects of alternate approaches.)

The average Simpson lexical size 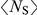 (Fig. 9) shows a pattern different to that of average happiness: we observe that a strong maximum appears on Friday with a drop through the weekend to a distinct low on Sunday. During the work week, Tuesday presents a minor low, with a climb up to Friday's high. This pattern remains the same if we choose different averaging schemes in generating a composite Simpson lexical size (see also Fig. S3).

To see further into these changes between days, we can generate word shift graphs for Simpson lexical size 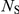. These word shift graphs (not shown) are simpler than those for average happiness as they depend only on changes in word frequency. Using the definition 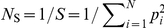, we obtain
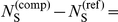


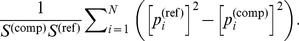
(4)We next define the individual percentage contribution in the shift in Simpson lexical size as
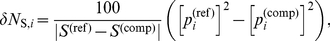
(5)where 
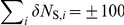
 depending on the sign of 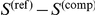. Note that the reversal of the reference and comparison elements in Eq. (5) reflects the fact that any one word increasing in frequency decreases overall diversity. Further, no other diversity measure (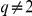) allows for a linear superposition of contributions such as we find in Eq. (5) , one of the reasons we provided earlier for choosing a lexical size based on Simpson's concentration.

Using Eq. (5) , we find Friday's larger value of 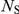 relative to Sunday's can be attributed primarily to changes in the frequency of around 100 words. Most of these words are those typically found at the start of a Zipf ranking of a text, though their ordering is of interest. A few words contributing the most to the shift are ‘I’, ‘RT’, ‘you’, ‘me’, and ‘my’. Decreases in the relative usage frequencies of personal pronouns may suggest a shift in focus away from the self and toward the less predictable, richer fare of Friday activities. Words specific to Friday naturally appear more frequently than on Sunday serving to reduce Friday's Simpson lexical size. Some examples include ‘#ff’, ‘follow’, ‘Friday’, ‘weekend’, and ‘tonight’ (#ff is an example of a hash tag, in this case representing a popular Friday custom of Twitter users recommending other users worth following).

### 6 Daily cycle

#### 6.1 Average Happiness of Hours of the Day

**Figure 10:**
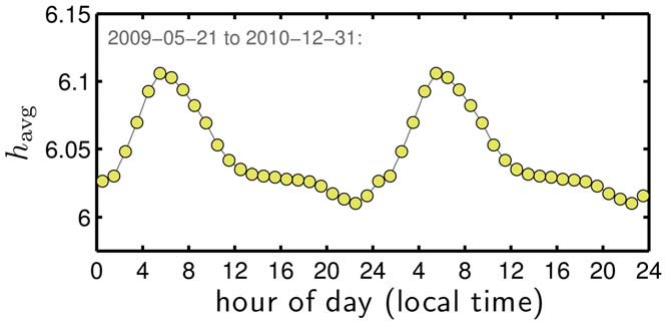
Average happiness level according to hour of the day, adjusted for local time. As for days of the week in Fig. 5, each data point represents an average of averages across days. The plot remains essentially unchanged if outlier dates marked in Fig. 3A are excluded. The maximum relative difference between the two plots is 0.08%. The daily pattern of happiness in tweets shows more variation than we observed for the weekly cycle (Fig. 5), here ranging from a low of 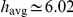 between 10 and 11 pm to a high of 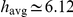 between 5 and 6 am.

We next examine how average happiness levels change throughout the day at the resolution of an hour. As shown in Fig. 10, the happiest hour of the day is 5 to 6 am, after which we see a steep decline until midday followed by a more gradual descent to the on-average low of 10 to 11 pm, and then a return to the daily peak through the night. An afternoon low is consistent with self-reported moods; Stone et al., in particular, observe a happiness dip in the afternoon [71], though here we see negativity decreasing well into the night. Our results are in contrast to some previous observations regarding blogs and Facebook [32], [42]; for example, Mihalcea and Liu [42] found a low occurring in the middle of the day (part of their analysis involved the ANEW study word list). The period 5–6 am marks ‘biological midnight’ when, for example, body temperature is typically lowest (see also [37]). People after this point in time are more likely to be rising for the day rather than extending the previous one, leading to a change in the kinds of mental states represented by active users.

**Figure 11:**
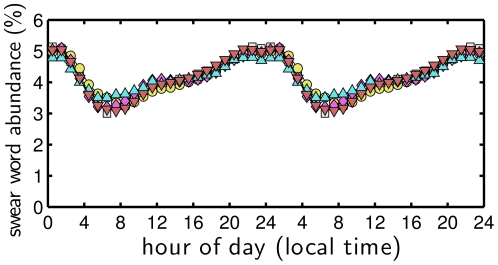
Normalized distributions of five example common expletives as a function of hour of the day.

We also find that usage rates of the most common profanities are remarkably similar and are roughly anticorrelated with the observed happiness cycle. Fig. 11 shows the normalized frequencies for five example profanities. Cursing follows a sawtooth pattern with a maximum occurring around 1 am, and the lowest relative usage of profanities matching up with the daily early morning happiness peak between 5 and 6 am. These patterns suggest a gradual, on-average, daily unraveling of the human mind.

#### 6.2 Word Shift Analysis

**Figure 12:**
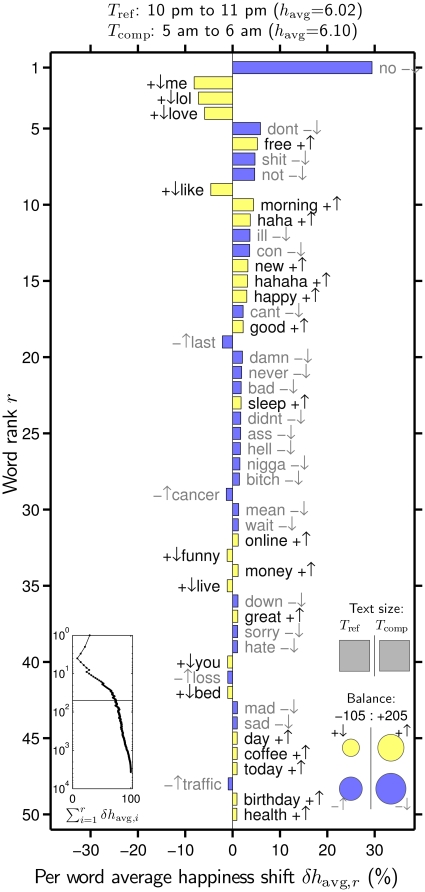
Word shift graph comparing the happiest hour (5 am to 6 am) relative to the least happy hour (10 pm to 11 pm). Days given equal weighting with outlier dates removed. (See Fig. S6 for word shifts based on alternate distributions.)

To give a deeper sense of the underlying moods reflected in the low and high of the day, we explore the word shift graph in Fig. 12, comparing tweets made in the hours of 5 to 6 am and 10 to 11 pm. For comparison, Fig. S6 shows word shift graphs under three averaging schemes.

The balance plot (bottom right inset) shows that 5 to 6 am is happier because of an overall preponderance of less abundant negative words and more abundant positive words, the former's contribution marginally larger than the latter. As the lower left inset cumulative plot shows, the first 50 words account for approximately 70% of the total shift. Thereafter, word shifts gradually bring the overall difference up to 100%, requiring all words to do so.

The first few salient, relatively positive words more abundant between 5 and 6 am (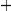 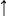) are ‘free’, ‘morning’ (likely appearing in good morning), ‘haha’, ‘new’, ‘hahaha’, ‘happy’, and ‘good’. These are joined with decreases in negative word prevalences (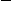 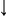) including most strongly ‘no’, as well as ‘don't’, ‘shit’, and ‘not’. Going against the overall trend are positive words used less often and pointing to a drop in social interactions, such as ‘me’, ‘lol’, ‘love’, ‘like’, ‘funny’, and ‘you’ (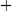 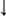). We also see more of the early morning negative ‘traffic’ (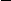 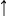). The word shift graph also holds suggestions of automated tweets; e.g., the word ‘cancer’ may refer to the Zodiac sign.

#### 6.3 Information Content

**Figure 13:**
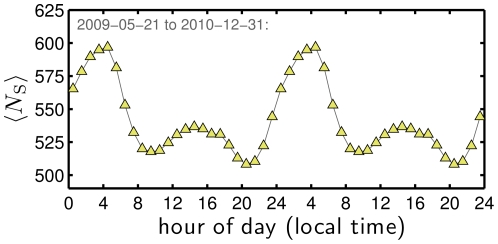
Average Simpson lexical size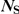 for time of day, corrected according to local time, and computed for each day with outlier days removed, and then averaged across days. See also Fig. S4 for a demonstration of the robustness of the form of 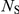 throughout the day under alternate averaging schemes.

In Fig. 13, we show that average Simpson lexical size 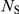 follows a daily cycle roughly similar in shape to average happiness. The peak through the night is more pronounced than for happiness, taking off around 9 pm, climbing until 5 to 6 am (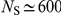); from there, 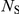 drops rapidly to a local minimum in the morning (9 to 10 am), and then rises slightly to reach a minor crest in the early afternoon before slowly declining to the day's minimum between 10 and 11 pm (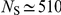). In examining the change in 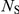 between the high at 5 to 6 am and the low in 10 to 11 pm, we see the first few contributions by rank are ‘I’, ‘a’, ‘the’, ‘de’ ‘me’, and ‘que’ which appear less frequently between 5 and 6 am. Most all other words making substantive contributions are prepositions and pronouns. The only word in the top 20 that becomes more frequent and thus effects a decrease in 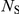, is the second ranked ‘RT’. Tweets thus appear to be more rich and less predictable during the night, with an apex near biological midnight. Another potential explanation may involve automated tweets, an analysis of which is beyond the scope of the present work.

Finally, we find that using alternate averaging schemes to create word frequency distributions for hour of the day yields remarkably little variation in 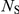 (see Fig. S4).

### 7 Happiness averages and dynamics for tweets containing keywords and phrases

We turn to our last area of focus: temporal happiness patterns for tweets containing specific text elements. We need not restrict ourselves to words, considering also, for example, short phrases (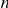-grams), dates, punctuation, emoticons, and phonemes. We examine various collections of text elements, ranging from long term importance (‘economy’), to contemporary topics (‘Obama’), to the everyday (‘today’ and ‘!’). In doing so, we are effectively generating opinion polls regarding certain topics. Recent related work has explored correlations between public opinion polls and Twitter sentiment levels [33], as well as the use of emotional levels gleaned from Twitter to predict stock market behavior [36]. Here, we add to these findings by showing how certain happiness trends based on keywords are clearly correlated with external events. At the same time, we find many keyword-based trends are relatively stable, and our interest turns to the average happiness level which we do find to be highly variable across keywords.

#### 7.1 Definition of Ambient Happiness

To facilitate comparisons, we now measure what we call ‘normalized happiness’ 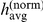, and ‘ambient happiness’ , rather than absolute happiness 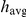, and which we define as follows. For a given text element, and a given pool of tweets (e.g., those falling in a specific month), we first find all tweets containing the text element. We measure the average happiness of the subset of tweets in two ways: including the text element's own happiness score for normalized happiness and excluding it for ambient happiness. To create 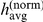 and 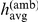, we subtract the average happiness of all tweets in the pool. In this way, we are able to separate out the effect of the text element, and can construct time series as the difference in happiness between the text element time series and the overall time series (Fig. 3).

**Figure 14:**
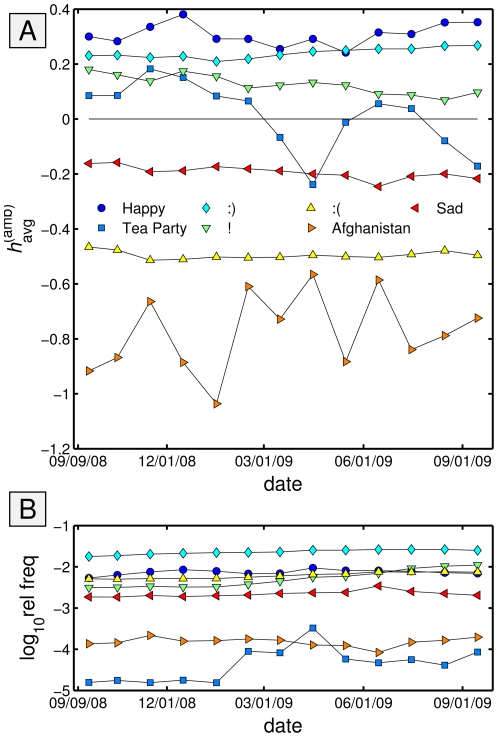
Ambient happiness 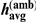 and occurrence frequency time series for some illustrative text elements. **A.** Ambient happiness is the average happiness of all words found co-occurring in tweets containing a given text element, with the background average happiness of all tweets removed (n.b., the text element's contribution is excluded). Binning is by calendar month and symbols are located at the center of each month. **B.** Fraction of tweets containing text elements.

In Fig. 14A, we show ambient happiness time series for seven example text elements, chosen so as to exhibit both a range of happiness scores and represent diverse topics and elements. The lower plot in Fig. 14B shows the relative normalized frequency of tweets containing each text element. The trend for tweets containing the word ‘happy’ is to maintain a positive differential of approximately +0.3 to +0.4 above the overall average happiness time series. By contrast, the counter of ‘sad’ hovers around 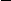
0.2. Words co-occurring with the emoticons ‘:)’ and ‘:(’ are strongly distinct in terms of happiness with means near +0.25 and 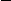
0.5. The exclamation point's ambient happiness time series is a positive one though clearly below that of ‘happy’ and ‘:)’, and we see a slight downward trend toward a neutral score of 0. Lastly, we show trends for two contemporary issues in the United States, ‘Tea Party’ and ‘Afghanistan’. Both phrases exhibit uneven signals, with ‘Tea Party’ reaching its lowest 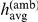 score when its usage is most frequent. ‘Afghanistan’ is not surprisingly strongly negative with ambient happiness scores consistently between 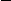
1.1 and 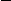
0.6.

#### 7.2 Overall Ambient Happiness for Specific Tweets

We next examine a selection of 100 handpicked keywords and text elements. As mentioned above, the ambient average happiness for tweets containing many of these terms are mostly stable over time, and in Table 2 we show overall ambient average happiness 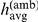 for the list, sorted in descending order. Our list is in no way exhaustive; rather it contains political keywords (‘Democrat’ and ‘Republican’), semantic differentials (‘right’ and ‘left’), terms relating to the economy (‘money’ and ‘Goldman Sachs’), families of related keywords (‘Jon Stewart’ and ‘Glenn Beck’), personal pronouns, emoticons, and so on. As such, the extremes (most and least happy words for example) are not to be presumed to remain so for larger sets of key words, and our main interest is in making comparisons of related terms. In Table 3, we present the same terms ordered according to Simpson lexical size 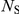. In computing each term's 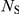, we exclude the term itself. For ease of comparison, we include Table 2 reordered by normalized happiness as Table S1.

**Table 2:** Selection of 100 text elements ordered by average ambient happiness 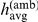.

Word	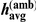	Total Tweets	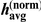	Word	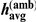	Total Tweets	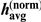
1. happy	+0.430	1.65e+07 (13)	+1.104 (1)	51. snow	−0.051	2.60e+06 (49)	+0.083 (39)
2. Christmas	+0.404	4.89e+06 (35)	+0.953 (3)	52. Jon Stewart	−0.052	5.21e+04 (97)	−0.024 (48)
3. vegan	+0.315	1.84e+05 (90)	−0.015 (46)	53. school	−0.056	9.26e+06 (24)	+0.050 (42)
4. :)	+0.274	1.04e+07 (20)	+0.630 (12)	54. Lehman Brothers	−0.078	8.50e+03 (100)	−0.721 (79)
5. family	+0.251	5.01e+06 (32)	+0.716 (7)	55. them	−0.090	1.54e+07 (15)	−0.280 (60)
6. :-)	+0.228	1.67e+06 (60)	+0.560 (16)	56. right	−0.090	1.92e+07 (10)	+0.126 (35)
7. our	+0.207	1.41e+07 (16)	+0.159 (33)	57. woman	−0.115	2.54e+06 (51)	+0.202 (30)
8. win	+0.204	7.98e+06 (26)	+0.924 (4)	58. left	−0.118	4.89e+06 (34)	−0.383 (63)
9. vacation	+0.200	9.35e+05 (67)	+0.817 (5)	59. me	−0.119	1.44e+08 (4)	+0.160 (32)
10. party	+0.170	6.44e+06 (29)	+0.679 (9)	60. election	−0.127	5.60e+05 (75)	−0.306 (61)
11. love	+0.164	4.67e+07 (6)	+0.977 (2)	61. Sarah Palin	−0.128	2.26e+05 (87)	−0.681 (76)
12. friends	+0.155	7.67e+06 (27)	+0.685 (8)	62. no	−0.132	9.51e+07 (5)	−1.415 (90)
13. hope	+0.149	1.18e+07 (18)	+0.515 (19)	63. rain	−0.134	3.23e+06 (41)	+0.050 (44)
14. coffee	+0.147	2.80e+06 (46)	+0.518 (18)	64. climate	−0.135	3.64e+05 (80)	−0.160 (51)
15. cash	+0.146	1.28e+06 (63)	+0.601 (14)	65. gay	−0.152	2.73e+06 (47)	−0.552 (72)
16. sun	+0.144	2.39e+06 (52)	+0.737 (6)	66. lose	−0.157	2.06e+06 (55)	−1.181 (86)
17. income	+0.137	5.10e+05 (76)	+0.621 (13)	67. they	−0.159	2.74e+07 (8)	−0.208 (58)
18. summer	+0.135	3.00e+06 (43)	+0.221 (29)	68. oil	−0.162	1.38e+06 (62)	−0.411 (65)
19. church	+0.131	1.81e+06 (58)	−0.016 (47)	69. cold	−0.162	3.67e+06 (36)	−0.546 (71)
20. Valentine	+0.127	2.47e+05 (84)	+0.593 (15)	70. I feel	−0.173	5.17e+06 (31)	−0.129 (50)
21. Stephen Colbert	+0.126	2.38e+04 (99)	+0.001 (45)	71. man	−0.175	1.59e+07 (14)	−0.163 (52)
22. USA	+0.113	2.16e+06 (54)	+0.325 (26)	72. Republican	−0.181	2.30e+05 (86)	−0.539 (70)
23. !	+0.106	3.44e+06 (40)	+0.195 (31)	73. sad	−0.187	3.56e+06 (38)	−1.366 (89)
24. winter	+0.101	1.26e+06 (64)	+0.050 (43)	74. gas	−0.193	1.02e+06 (65)	−0.471 (67)
25. God	+0.099	8.58e+06 (25)	+0.468 (20)	75. economy	−0.203	6.09e+05 (73)	−0.525 (69)
26. hot	+0.095	7.12e+06 (28)	−0.172 (54)	76. Obama	−0.205	2.98e+06 (44)	−0.173 (55)
27. ;)	+0.094	2.61e+06 (48)	+0.326 (25)	77. Democrat	−0.226	9.32e+04 (93)	−0.384 (64)
28. Jesus	+0.094	2.03e+06 (56)	+0.247 (28)	78. Congress	−0.231	3.92e+05 (79)	−0.580 (74)
29. today	+0.092	2.56e+07 (9)	+0.126 (36)	79. hell	−0.250	6.27e+06 (30)	−1.551 (96)
30. kiss	+0.072	1.70e+06 (59)	+0.632 (11)	80. sick	−0.262	3.58e+06 (37)	−1.630 (97)
31. yes	+0.056	1.16e+07 (19)	+0.321 (27)	81. Muslim	−0.262	2.15e+05 (88)	−0.569 (73)
32. tomorrow	+0.054	1.04e+07 (21)	+0.086 (38)	82. war	−0.270	1.96e+06 (57)	−2.040 (100)
33. you	+0.052	1.73e+08 (3)	+0.111 (37)	83. Pope	−0.277	1.52e+05 (91)	−0.316 (62)
34. heaven	+0.041	7.42e+05 (71)	+0.674 (10)	84. hate	−0.282	9.65e+06 (23)	−1.520 (94)
35. ;-)	+0.041	9.39e+05 (66)	+0.395 (23)	85. Glenn Beck	−0.282	1.14e+05 (92)	−0.776 (82)
36. we	+0.035	3.91e+07 (7)	+0.146 (34)	86. Islam	−0.299	1.87e+05 (89)	−0.710 (78)
37. yesterday	+0.033	3.08e+06 (42)	−0.168 (53)	87. George Bush	−0.333	3.23e+04 (98)	−0.747 (80)
38. dark	+0.031	1.58e+06 (61)	−0.766 (81)	88. Goldman Sachs	−0.337	5.27e+04 (96)	−0.984 (84)
39. ?	+0.030	2.32e+06 (53)	−0.503 (68)	89. depressed	−0.339	2.81e+05 (82)	−1.541 (95)
40. RT	+0.028	3.39e+08 (1)	−0.443 (66)	90. Senate	−0.340	4.48e+05 (78)	−0.601 (75)
41. Michael Jackson	+0.018	8.26e+05 (70)	−0.213 (59)	91. BP	−0.355	5.82e+05 (74)	−0.902 (83)
42. night	+0.014	1.71e+07 (12)	+0.074 (40)	92. gun	−0.367	6.81e+05 (72)	−1.476 (93)
43. life	+0.012	1.40e+07 (17)	+0.422 (22)	93. drugs	−0.382	5.10e+05 (77)	−1.452 (91)
44. health	−0.000	2.58e+06 (50)	+0.447 (21)	94. headache	−0.437	8.57e+05 (69)	−1.881 (98)
45. sex	−0.008	3.55e+06 (39)	+0.542 (17)	95. :-(	−0.455	3.40e+05 (81)	−1.174 (85)
46. work	−0.010	1.84e+07 (11)	−0.174 (56)	96. :(	−0.472	2.89e+06 (45)	−1.288 (88)
47. girl	−0.010	1.01e+07 (22)	+0.331 (24)	97. Afghanistan	−0.703	2.74e+05 (83)	−1.458 (92)
48. boy	−0.026	4.93e+06 (33)	+0.062 (41)	98. mosque	−0.709	6.98e+04 (95)	−0.694 (77)
49. I	−0.048	3.08e+08 (2)	−0.062 (49)	99. flu	−0.735	9.01e+05 (68)	−1.912 (99)
50. commute	−0.048	9.01e+04 (94)	−0.206 (57)	100. Iraq	−0.773	2.39e+05 (85)	−1.282 (87)

The number of tweets and the value of normalized happiness 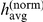 (where the happiness value of the text element itself is included) are listed in the third and fourth columns, with the ranking of the text element according to these quantities shown in brackets. For this list of text elements, we obtained additional happiness scores for phrases, punctuation, emoticons, etc., using Mechanical Turk. All pattern matches with tweets were case-insensitive. Table S1 shows the same table sorted by normalized happiness 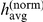.

**Table 3:** The same keywords and text elements as listed in Table 2 sorted according to the Simpson lexical size 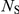 for all tweets containing them.

Word	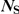	Total Words	Frac top 50K	Word	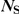	Total Words	Frac top 50K
1. RT	1019.5	4.751e+09 (1)	0.653 (100)	51. Iraq	235.5	3.722e+06 (84)	0.832 (68)
2. ?	662.1	2.608e+07 (58)	0.731 (98)	52. Jon Stewart	234.9	7.053e+05 (97)	0.836 (62)
3. !	621.1	3.682e+07 (50)	0.742 (97)	53. Senate	233.7	6.791e+06 (78)	0.826 (71)
4. USA	501.5	3.150e+07 (54)	0.751 (94)	54. happy	232.8	2.041e+08 (17)	0.834 (65)
5. no	487.3	1.431e+09 (5)	0.763 (93)	55. climate	231.7	5.245e+06 (80)	0.813 (81)
6. ;-)	476.9	1.323e+07 (67)	0.75 (95)	56. yes	230.0	1.484e+08 (21)	0.846 (50)
7. ;)	389.2	3.379e+07 (52)	0.791 (86)	57. today	225.3	3.802e+08 (9)	0.883 (20)
8. war	386.2	2.901e+07 (56)	0.785 (88)	58. election	220.7	8.632e+06 (75)	0.847 (47)
9. Goldman Sachs	379.5	7.183e+05 (96)	0.766 (92)	59. summer	219.1	4.471e+07 (42)	0.864 (39)
10. gay	377.6	3.823e+07 (46)	0.823 (77)	60. Christmas	215.7	6.330e+07 (35)	0.862 (41)
11. me	368.4	2.136e+09 (4)	0.829 (70)	61. rain	215.1	4.620e+07 (41)	0.836 (61)
12. :-)	362.3	2.280e+07 (61)	0.773 (91)	62. girl	214.0	1.513e+08 (20)	0.873 (32)
13. Islam	355.2	2.776e+06 (89)	0.678 (99)	63. I feel	214.0	7.141e+07 (34)	0.901 (4)
14. :)	347.1	1.313e+08 (24)	0.775 (90)	64. kiss	212.7	2.463e+07 (59)	0.845 (51)
15. Muslim	343.9	3.327e+06 (86)	0.779 (89)	65. God	211.6	1.298e+08 (25)	0.884 (18)
16. Michael Jackson	335.0	1.029e+07 (71)	0.803 (83)	66. school	211.2	1.328e+08 (23)	0.88 (25)
17. Obama	325.8	4.412e+07 (43)	0.825 (74)	67. coffee	209.1	3.926e+07 (45)	0.878 (27)
18. Lehman Brothers	324.5	1.161e+05 (100)	0.743 (96)	68. Afghanistan	208.8	3.898e+06 (83)	0.793 (85)
19. :-(	312.5	4.798e+06 (81)	0.804 (82)	69. heaven	208.3	1.075e+07 (69)	0.864 (38)
20. health	312.4	3.817e+07 (47)	0.826 (72)	70. left	207.8	8.017e+07 (31)	0.873 (31)
21. gas	311.8	1.580e+07 (65)	0.822 (78)	71. family	207.8	7.700e+07 (32)	0.873 (30)
22. Jesus	311.4	3.011e+07 (55)	0.831 (69)	72. them	205.1	2.672e+08 (12)	0.893 (9)
23. :(	304.5	3.802e+07 (48)	0.798 (84)	73. sad	203.6	5.482e+07 (36)	0.886 (17)
24. hot	298.3	9.826e+07 (28)	0.847 (46)	74. night	203.1	2.429e+08 (13)	0.883 (21)
25. cash	298.0	1.909e+07 (63)	0.832 (66)	75. hell	202.7	9.000e+07 (30)	0.883 (19)
26. vegan	290.9	2.696e+06 (90)	0.845 (54)	76. mosque	198.3	1.081e+06 (95)	0.82 (80)
27. George Bush	288.0	4.546e+05 (98)	0.847 (48)	77. tomorrow	198.1	1.516e+08 (19)	0.892 (11)
28. BP	285.2	8.957e+06 (74)	0.791 (87)	78. friends	197.5	1.242e+08 (27)	0.886 (16)
29. man	283.3	2.333e+08 (15)	0.845 (52)	79. vacation	197.1	1.341e+07 (66)	0.876 (28)
30. sex	276.2	5.186e+07 (37)	0.844 (57)	80. snow	195.6	3.698e+07 (49)	0.881 (22)
31. Sarah Palin	275.4	3.194e+06 (87)	0.842 (58)	81. yesterday	192.7	5.003e+07 (39)	0.887 (14)
32. we	272.4	6.434e+08 (6)	0.869 (34)	82. right	190.5	2.854e+08 (10)	0.887 (15)
33. flu	270.8	1.279e+07 (68)	0.826 (73)	83. church	189.1	2.668e+07 (57)	0.879 (26)
34. income	270.7	7.681e+06 (76)	0.835 (63)	84. cold	188.4	5.116e+07 (38)	0.9 (5)
35. I	269.8	4.590e+09 (2)	0.881 (23)	85. lose	187.2	3.335e+07 (53)	0.881 (24)
36. oil	267.1	2.147e+07 (62)	0.825 (75)	86. sick	186.6	4.985e+07 (40)	0.899 (6)
37. Democrat	262.4	1.469e+06 (94)	0.832 (67)	87. economy	186.5	9.512e+06 (73)	0.847 (49)
38. drugs	261.7	7.633e+06 (77)	0.862 (40)	88. dark	186.1	2.403e+07 (60)	0.868 (36)
39. our	257.6	2.394e+08 (14)	0.869 (35)	89. Pope	185.3	2.268e+06 (91)	0.84 (59)
40. boy	256.7	7.174e+07 (33)	0.857 (42)	90. win	185.1	1.261e+08 (26)	0.825 (76)
41. Glenn Beck	252.3	1.740e+06 (92)	0.851 (44)	91. life	180.4	2.210e+08 (16)	0.892 (10)
42. Stephen Colbert	251.0	2.972e+05 (99)	0.844 (55)	92. woman	178.8	4.151e+07 (44)	0.874 (29)
43. Valentine	248.4	3.169e+06 (88)	0.822 (79)	93. work	178.3	2.791e+08 (11)	0.898 (7)
44. party	242.9	9.466e+07 (29)	0.844 (56)	94. depressed	175.2	4.108e+06 (82)	0.906 (2)
45. gun	241.9	1.030e+07 (70)	0.836 (60)	95. sun	166.9	3.622e+07 (51)	0.849 (45)
46. winter	240.2	1.871e+07 (64)	0.854 (43)	96. commute	165.0	1.470e+06 (93)	0.887 (13)
47. Republican	239.8	3.607e+06 (85)	0.845 (53)	97. hope	157.2	1.853e+08 (18)	0.89 (12)
48. they	239.8	4.749e+08 (8)	0.896 (8)	98. love	149.9	6.409e+08 (7)	0.865 (37)
49. you	239.2	2.484e+09 (3)	0.871 (33)	99. headache	126.7	1.005e+07 (72)	0.907 (1)
50. Congress	236.8	6.221e+06 (79)	0.834 (64)	100. hate	106.5	1.382e+08 (22)	0.902 (3)

Keywords themselves are not included in the calculation of 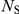. The third and fourth columns show the total number of words (other than the keyword) used to measure 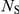 and the fraction of these words that are in our fixed list of 50,000 words (the higher the better). The numbers in brackets give rankings.

We observe many interesting patterns and we invite the reader to explore the tables beyond the observations we record here. We begin with the highest and lowest rankings of ambient happiness 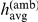, for our list, finding them to be sensible. The two top ranked words are ‘happy’ (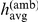 = 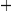
0.430) and ‘Christmas’ (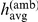 = 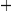
0.404), and the last two are ‘flu’ (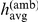 = 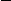
0.735), and ‘Iraq’ (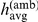 = 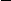
0.773). When we include the text element's score itself (see Table S1), the order shifts somewhat with ‘happy’ and ‘love’ at the top and ‘flu’ and ‘war at the bottom.

An important finding is that the average happiness of text elements as assessed through Mechanical Turk and their ambient happiness correlate very strongly (Spearman's correlation coefficient 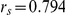, 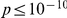), as do ambient and normalized happiness (Spearman's correlation coefficient 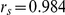, 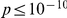). In terms of emotional content, individual text elements therefore appear to be well connected to their contexts. We caution again that this does not imply individual sentences will rigidly exhibit such structure, but rather do so on average.

We nevertheless find some scores move substantially when the text element's score is included; For example, ‘vegan’ ranks 3rd with 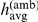 = 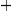
0.315, and 46th with 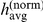 = 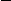
0.015; ‘church’ ranks 19th with 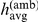 = 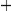
0.131, and 47th with 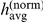 = 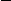
0.016; and ‘sex’ ranks 45th with 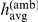 = 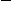
0.008, but rises to 17th with 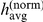 = 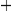
0.542.

For financial terms, we see tweets mentioning the dissolved firm of ‘Lehmann Brothers’ and ‘Goldman Sachs’ are both negative (more so in the latter's case) while relatively high in lexical size (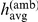 = 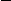
0.078, 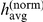 = 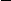
0.721, 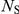 = 324 and 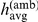 = 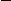
0.337, 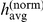 = 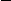
0.984, 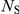 = 379). We see ‘economy’ is pegged at the same somewhat negative level as political terms (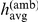 = 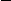
0.203) but conversely returns a low information level (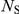 = 186). By contrast, the more personal term ‘cash’ appears in highly positive tweets with 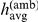 = 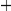
0.146.

Tweets referring to United States politics are below average in happiness with ‘Obama’, ‘Sarah Palin’, and ‘George Bush’ registering 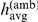 = 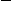
0.205, 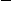
0.128, and 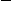
0.333 (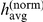 = 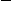
0.173, 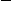
0.681, and 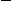
0.747). At the same time, these political figures all correspond to large lexical sizes (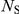 = 326, 275, and 288 respectively). A number of other political words also fair poorly such as ‘election’ (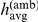 = 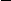
0.127, 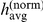 = 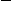
0.306), ‘Senate’ (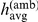 = 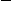
0.340, 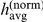 = 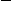
1.541), and ‘Congress’ (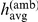 = 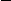
0.231, 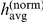 = 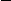
0.580). The ambient happiness for ‘Senate’ is one rank lower than ‘depressed’ and one higher than ‘BP’. “Republican’ exceeds ‘Democrat’ in ambient happiness (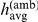 = 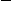
0.181 versus 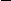
0.226) but trails in information content (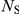 = 240 versus 262).

Tweets involving the word ‘war’ rank high in information ( = 386) and are unsurprisingly low in terms of happiness (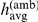 = 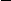
0.270,  = 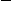
2.040). The keywords ‘Muslim’, ‘Islam’, and ‘mosque’ also register some of the lower ambient happiness scores: 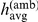 = 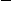 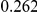, 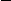 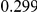, and 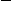 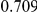. (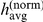 = 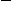 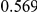, 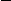 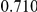, and 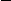 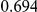).

Generally, personal pronouns tell a positive prosocial story with ‘our’ and ‘you’ outranking ‘I’ and ‘me’ in happiness (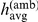 = 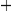
0.207 and +0.052 versus −0.048 and 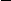
0.119). The least happy pronoun on our list is the easily demonized ‘they’ at 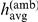 = 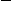
0.159. However, tweets involving pronouns indicating self appear to be more information rich in comparison with those pointing to others: ‘me’ and ‘we’ rank 11th and 32nd (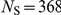 and 272), while ‘they’ and ‘them’ rank 48th and 72nd overall ( and 205).

The ambient words in tweets containing ‘summer’ are slightly happier than those containing ‘winter’ but are less diverse: 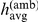 = 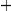
0.135 and 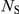 = 219 versus 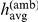 = 
0.101 and 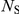 = 240. Other semantic differentials show reasonable differences. Tweets with ‘hot’ are happier than those with ‘cold’ (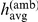 = 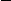
0.095 versus 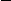
0.162). The sequence ‘yesterday’, ‘today’, and ‘tomorrow’ suggests a preferential ordering of present, future, and past with corresponding ambient happiness scores of 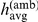 = 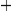
0.033, +0.092, and +0.054.

Emoticons in increasing order of happiness are ‘:(’, ‘:-(’, ‘;-)’, ‘;)’, ‘:-)’, and ‘:)’ with 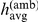 spanning 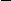
0.472 to +0.274 (normalized happiness preserves the ordering with the range increasing to −1.288 to +0.630). In terms of increasing information level, the order is ‘:(’, ‘:-(’, ‘:)’, ‘:-)’, ‘;)’, and ‘;-)’ with 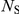 ranging from 305 to 477. We see that happy emoticons correspond to higher levels of both ambient happiness and information but the ordering changes in a way that appears to reflect a richness associated with cheekiness and mischief: the two emoticons involving semi-colon winks are third and fourth in terms of happiness but first and second for information.

Tweets involving the ‘fake news’ comedian Stephen Colbert are both happier and of a higher information level than those concerning his senior colleague Jon Stewart (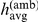 = 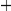
0.126 and 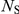 = 251 versus 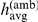 = 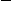
0.052 and 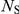 = 235). By contrast, tweets mentioning Glenn Beck are lower in happiness than both Colbert and Stewart but comparable to Colbert in information content (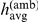 = 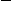
0.282 and 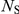 = 252).

As noted above, the exclamation point garners a positive ambient happiness (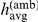 = 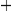
0.106), and this is clearly above the question mark's score of 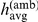 = 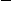
0.030. They have essentially equal values for information content, ranking second (‘?’, 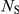 = 662) and third (‘!’, 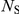 = 621) overall. These high values of 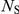 are sensible due to the versatility of punctuation, and RT's top ranking reflects the diverse nature of status updates shared by users.

**Figure 15:**
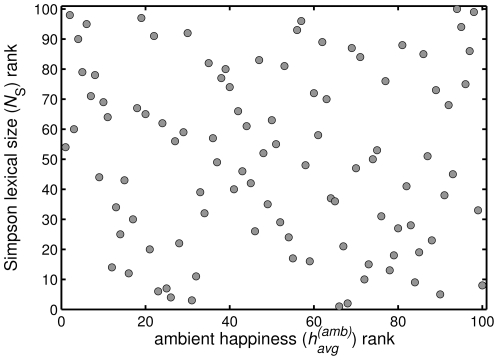
For the 100 keywords and text elements listed in Table 2, a rank-rank plot of Simpson lexical size 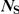 versus ambient happiness 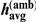. The two quantities show no correlation with Spearman's correlation coefficient measuring 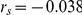 (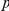-value 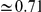).

A reflection on the preceding survey suggests that groups of related terms may possess positive, negative, or neutral correlation between happiness and information content. Overall, for our set of 100 keywords and text elements, we measure Spearman's correlation coefficient as 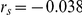 (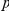-value 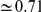), indicating no correlation, a finding supported visually in Fig. 15. We thus have strong evidence that the two main quantities of interest that we have studied in this paper are, generally speaking, independent. Several observations follow. First of all, this independence warrants further study for other texts and, if possible, explanation. Second, both quantities (or analogs) should be reported in any characterization of large-scale texts. Third, for specific subfamilies of texts, any finding of a statistically and quantitatively significant correlation between happiness and lexical size is of interest and deserving of further investigation.

#### 7.3 Analysis of Four Example Ambient Happiness Time Series

**Figure 16:**
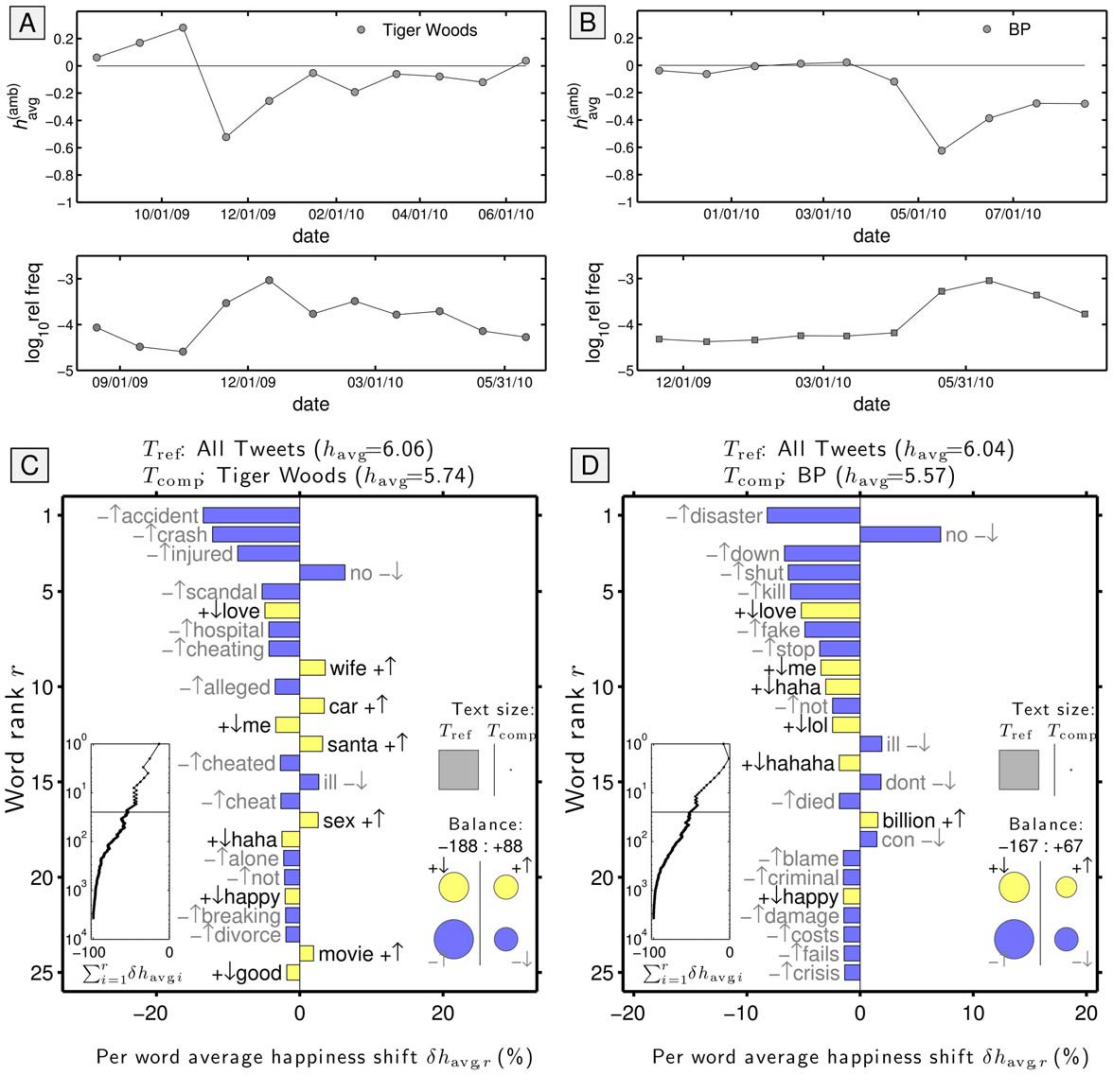
Ambient happiness time series and word shift graphs for tweets containing the keywords ‘Tiger Woods’ and ‘BP’. Ambient happiness of a keyword is 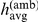 for all words co-occurring in tweets containing that keyword, with the overall trend for all tweets subtracted. The word shift graphs are for tweets made during the worst month and the ensuing one–November and December, 2009 for ‘Tiger Woods’ and May and June, 2010 for ‘BP’.

**Figure 17:**
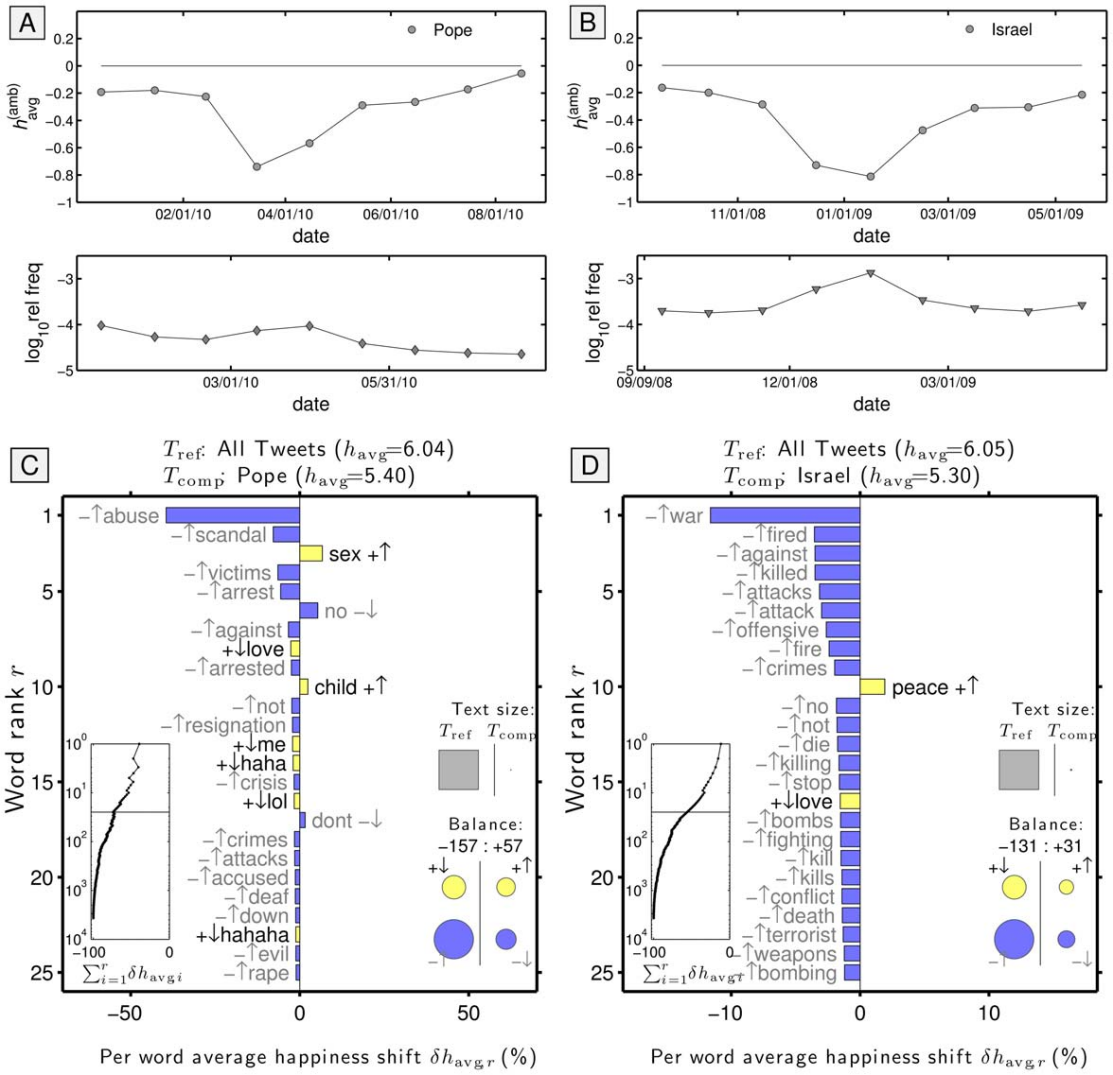
Time series and word shift graphs for tweets containing the keywords ‘Pope’ and ‘Israel’. The word shift graphs are for the time periods March and April, 2010 for ‘Pope’ and January and February, 2010 for ‘Israel.’ See Fig. 16's caption for more details.

In Figs. 16 and 17, we present four ambient happiness time series for tweets containing the terms ‘Tiger Woods’, ‘BP’, ‘Pope’, and ‘Israel’. For each example, we include word shift graphs that illuminate the difference in word composition and tone for the most extreme month and the following month in comparison to that of all tweets during the same period. All of these topics involve a negative event or events leading to global media coverage.

In Fig. 16A, we show that the ambient happiness time series for Tiger Woods drops abruptly in November, 2009 when his extramarital affairs famously became public after Woods crashed his car into a fire hydrant around Thanksgiving. The National Enquirer had published a claim of infidelity a few days before, and knowledge of Woods's manifold extra-marital relationships were soon widely being reported in the general media. Tweets concerning Woods, the world's longstanding number one golfer at the time, dropped sharply in happiness level and then rebounded over the next few months to a slightly below average steady state. The jump in media coverage is reflected in the number of tweets (middle plot). As the word shift graph shows for November and December, 2009, negative words such as ‘accident’, ‘crash’, ‘scandal’, ‘hospital’, and ‘divorce’ pull the average happiness down below the baseline. The words ‘car’ and ‘sex’, in isolation considered to be relatively happy words, here improved 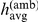 for Woods, showing one of the potential failings of our word-centric approach. Nevertheless, the net effect is clear and such microscopic errors are overcome for large enough texts. Overall, of the four word types, the largest contribution to the drop comes from an increase in the use of negative words (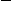 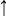).

In Fig. 16B, we see the decline of British Petroleum's ambient happiness following the April 20, 2010 explosion and collapse of the deep sea drilling platform Deepwater Horizon in the Gulf of Mexico. The well proved to be extremely difficult to cap and oil spewed into the Gulf for nearly three months. In comparing tweets containing ‘BP’ to all tweets in May and June, 2010, we find a drop in 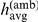 of 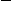
0.47 due to relative increases in words such as ‘disaster’, ‘down’, ‘shut’, ‘kill’, ‘damage’, as well as ‘blame’, ‘criminal’, and ‘costs’, and decreases in the appearance of ‘love’, ‘me’, ‘haha’, and ‘lol’. Similar to the Tiger Woods word shift, we see the more frequent use of relatively negative words (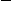 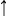) and the less frequent use of more positive words (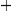 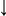); both contribute substantially to the sharp decrease in average happiness.

In Fig. 17A, we track the ambient happiness of the keyword ‘Pope’ over a nine month period starting with December, 2009. While the relative frequency of tweets containing ‘Pope’ changes little, a clear minimum in 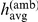 occurs in March, 2010. The Catholic Church's long running child molestation scandal was brought into even sharper focus during this month, notably via a Papal apology to the Irish church, and the New York Times publishing documents concerning Pope Benedict's past decisions on child molestation cases, opening up a highly charged dialogue between the media and the Vatican. In the word shift graph, we see the nadir of March and April arising from the more frequent use of negative words such as ‘abuse’, ‘scandal’, ‘victims’, ‘arrest’, and ‘resignation’, and the drop in positive words such as ‘love’, ‘me’, and ‘haha’. The increased use of the words ‘sex’ (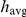 = 8.05) and ‘child’ (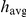 = 7.08) in tweets containing ‘Pope’ goes against the trend (see remarks above for Tiger Woods). The overall picture is similar to that for Tiger Woods and BP: the increase in negative words (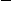 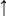) is the main reason ‘Pope’ tweets are far below the average happiness level for March and April, 2010.

Our last example, Fig. 17B, shows ambient happiness for tweets involving ‘Israel’ from September, 2008 through to May, 2009. The drop in November and December reaching a minimum in January matches with the Gaza War, fought between Israel and Hamas. The increase in ‘Israel’ tweets also captures the increase in media reporting during this conflict. In the top ranked 25 words contributing to the strong decrease for January and February relative to the overall time series, we see the major changes primarily coming from the more frequent use of negative words (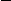 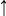) such as ‘war’, ‘fire’, ‘kill’, ‘attack’, ‘bombs’, and ‘conflict’. Against this rather bleak sequence of negative word shifts, we may take some solace in seeing the word ‘peace’ appear more often (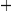 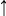). Once again, we see that the overall drop is due largely to an increase in negative words (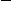 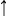) and to a lesser extent a decrease in positive words (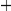 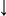).

### 8 Concluding remarks

In analysing temporal patterns of happiness and information content for the very large data set generated by Twitter thus far, we have been able to uncover results ranging across many timescales and topics. The weekly and daily cycles in particular appear to be robust and suggestive of universal forms, accepting that the seven day week cycle is an historical and cultural artifact. With our greatly expanded word list as analysed using Mechanical Turk, labMT 1.0 (Data Set S1), we believe we have provided a substantial methodological advance in the measurement of sentiment in large-scale texts. We hope that our tunable hedonometer and the associated words provided in the Supplementary Information will be of use to other researchers.

An essential part of our comparative analyses is the word shift graph, which we have primarily used here for happiness. These provide us with a detailed view of why two texts differ based on changes in word frequency. These graphs, and their future iterations, should be of use in a range of fields where size distributions are compared through summary statistics (e.g., understanding how species diversity in ecological populations may differ as a result of changes in individual species abundances).

As we have described, the metadata accompanying Twitter messages contains more information than time stamps. Future research will naturally address (and go beyond) geographic variations, particularly for the United States; the change in expressions over time for individuals and the possibility of correlation or contagion of sentiment; effects of popularity as measured by follower count on users' expressions; and the possibility of fine-scale emotional synchronization between individuals based on directed messages [72]. In terms of methodology, our hedonometer could be improved by incorporating happiness estimates for common 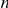-grams, e.g., 2-grams such as ‘child abuse’ and ‘sex scandal’ as well as negated sentiments such as ‘not happy’. This would improve the reliability of our happiness (and information) measures without losing the transparency of our current approach, and begin to address issues of words being used in specific contexts and words having multiple meanings. Language detection for tweets, recently added by Twitter to their metadata, allows for language specific analyses. The robustness of the measure we have used in our present work further suggests that we should be able to determine conversion factors between the scores of different text-based hedonometers. For measures of information content, an improved handling of very long word lists, and potential incorporation of 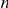-grams, will allow us to use Shannon's entropy in future work.

As we have seen in both the work of others and ours, Twitter and similar large-scale, online social networks have thus far provided good evidence that scientifically interesting and meaningful patterns can be extracted from these massive data sources of human behavior. The extent to which small-scale patterns can be elicited, e.g., for rare topics, also remains an open question, as does the true generalizability to the broader population. Whatever the case, Twitter is currently a substantial, growing element of the global media and is worth studying in its own right, just as a study of newspapers would seem entirely valid. And while current evidence suggests ‘instant polls’ created by remote-sensing text analysis methods are valid, and that these instruments complement and may in some cases improve upon traditional surveys, analysts will have to remain cognizant of the ever present problem of users gaming online expression systems to misinform.

Finally, the era of big data social sciences has undoubtedly begun. Rather than being transformed or revolutionized we feel the correct view is that the social sciences are expanding beyond a stable core to become data-abundant fields. In a data-abundant science, the challenge moves first to description and pattern finding, with explanation and experiments following. Instead of first forming hypotheses, we are forced to spend considerable time and effort simply describing. The approaches applicable for a data-scarce science still remain of the same value but new, vast windows into social and psychological behaviour are now open, and new tools are available and being developed to enable us to take in the view.

## Methods

We defined a word as any contiguous set of characters bounded by white space and/or a small set of punctuation characters. We therefore included all misspellings, words from any language used on Twitter, hyperlinks, etc. All pattern matches we made were case-insensitive, and we did not perform stemming (e.g., ‘love’ and ‘loved’ were counted separately).

The data feed from Twitter was provided in XML and JSON formats [46]. Early on, the data feed contained many repeated tweets, and while the fraction of duplicates dropped substantially over time, we nevertheless were obliged to check for and remove all such tweets. (Due to these various changes, all measures involving emoticons are derived from the time series up until only November 9, 2009.)

In measuring and comparing information content, a computational difficulty with the Twitter data set lies in accommodating the sheer number of distinct words. We found approximately 230 million unique words (including URLs) from a random sample of 25% of the tweets in our database. We determined that restricting our attention to a more manageable set of the first 50,000 most frequent words would be sufficient for highly accurate estimates of generalized entropy 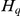 with 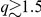, and therefore Simpson's concentration 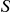 when 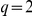. We did not use Shannon's entropy [73] since it converges too slowly (akin to 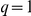) for the skew we observed in the Twitter word frequency distribution. Importantly, in fixing a list of words, we were able to account for information content differences between texts at the level of words.

Consequently, we recorded the frequencies for this specific set of 50,000 words at the level of hours and days. Note that we also always recorded the total number of words for any particular subset of tweets, so that our word probabilities were correctly normalized.

## Supporting Information

Data Set S1 **Data from Mechanical Turk study.** labMT 1.0 = language assessment by Mechanical Turk 1.0. In the supplementary tab-delimited file named Data Set S1, we provide our set of 10,222 words, their average happiness evaluations according to users on Mechanical Turk, and other information as described below. Please cite the present paper when using this word set. Within papers, we suggest using the abbreviation labMT 1.0 when referencing this data set. The words are ordered according to average happiness (descending), and the file contains eight columns: (1) word, (2) rank, (3) average happiness (50 user evalutions), (4) standard deviation of happiness, (5) Twitter rank, (6) Google Books rank, (7) New York Times rank, (8) Music Lyrics rank. The last four columns correspond to the ranking of a word by frequency of occurrence in the top 5000 words for the specified corpus. A double dash ‘–’ indicates a word was not found in the most frequent 5000 words for a corpus. Please see the main paper for more information regarding this data set.
(TXT)

Figure S1 **High resolution, zoomable version of** **Fig. 3** **in the main text.**
(TIFF)

Figure S2 **Simple average happiness time series plots.** The time series is extended to include part of September, 2011, and shows a drop corresponding to the tenth anniversary of the 9/11 terror attacks in the United States.
(TIFF)

Figure S3 **Simpson lexical size as a function of day of the week using three different ways of creating distributions.** Compare with Fig. 9.
(TIFF)

Figure S4
**Average Simpson lexical size** 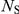 **for time of day, corrected according to local time, using three different ways of creating distributions.** Compare with Fig. 13.(TIFF)

Figure S5 **Word shift graph comparing the Saturdays to Tuesdays using three approaches to generating the day word distributions.** **A.** Days combined without regard to sampling frequency, **B.** Days given equal weighting, **C.** days given equal weighting with outlier dates removed. While words do move around the overall pattern remains similar. Compare with Fig. 8.
(TIFF)

Figure S6 **Word shift graph comparing the happiest hour (5 am to 6 am) relative to the least happy hour (10 pm to 11 pm) using three approaches to generating the day word distributions.** **A.** Days combined without regard to sampling frequency, **B.** days given equal weighting, **C.** days given equal weighting with outlier dates removed. Compare with Fig. 12 and see Fig. 8 and related text for further explanation.
(TIFF)

Figure S7 **Word shift graph for Bailout of the U.S. financial system, 2008/09/29, relative to 7 days before and 7 days after combined.**
(TIFF)

Figure S8 **Word shift graph for Halloween, 2008/10/31, relative to 7 days before and 7 days after combined.**
(TIFF)

Figure S9 **Word shift graph for Thanksgiving, 2008/11/27, relative to 7 days before and 7 days after combined.**
(TIFF)

Figure S10 **Word shift graph for Christmas Eve, 2008/12/24, relative to 7 days before and 7 days after combined.**
(TIFF)

Figure S11 **Word shift graph for Christmas Day, 2008/12/25, relative to 7 days before and 7 days after combined.**
(TIFF)

Figure S12 **Word shift graph for New Years's Eve, 2008/12/31, relative to 7 days before and 7 days after combined.**
(TIFF)

Figure S13 **Word shift graph for New Year's Day, 2009/01/01, relative to 7 days before and 7 days after combined.**
(TIFF)

Figure S14 **Word shift graph for Valentine's Day, 2009/02/14, relative to 7 days before and 7 days after combined.**
(TIFF)

Figure S15 **Word shift graph for Easter, 2009/04/12, relative to 7 days before and 7 days after combined.**
(TIFF)

Figure S16 **Word shift graph for Swine Flu pandemic, 2009/04/27, relative to 7 days before and 7 days after combined.**
(TIFF)

Figure S17 **Word shift graph for Father's Day (U.S.), 2009/06/21, relative to 7 days before and 7 days after combined.**
(TIFF)

Figure S18 **Word shift graph for Michael Jackson's death, 2009/06/25, relative to 7 days before and 7 days after combined.**
(TIFF)

Figure S19 **Word shift graph for Fourth of July, Independence Day, 2009/07/04, relative to 7 days before and 7 days after combined.**
(TIFF)

Figure S20 **Word shift graph for Distributed Denial of Service Attack on Twitter, 2009/08/06, relative to 7 days before and 7 days after combined.**
(TIFF)

Figure S21 **Word shift graph for Patrick Swayze's death, 2009/09/14, relative to 7 days before and 7 days after combined.**
(TIFF)

Figure S22 **Word shift graph for Halloween, 2009/10/31, relative to 7 days before and 7 days after combined.**
(TIFF)

Figure S23 **Word shift graph for Thanksgiving, 2009/11/26, relative to 7 days before and 7 days after combined.**
(TIFF)

Figure S24 **Word shift graph for Christmas Eve, 2009/12/24, relative to 7 days before and 7 days after combined.**
(TIFF)

Figure S25 **Word shift graph for Christmas Day, 2009/12/25, relative to 7 days before and 7 days after combined.**
(TIFF)

Figure S26 **Word shift graph for New Year's Eve, 2009/12/31, relative to 7 days before and 7 days after combined.**
(TIFF)

Figure S27 **Word shift graph for New Year's Day, 2010/01/01, relative to 7 days before and 7 days after combined.**
(TIFF)

Figure S28 **Word shift graph for Valentine's Day, 2010/02/14, relative to 7 days before and 7 days after combined.**
(TIFF)

Figure S29 **Word shift graph for 2010 Chile earthquake, 2010/02/27, relative to 7 days before and 7 days after combined.**
(TIFF)

Figure S30 **Word shift graph for Easter, 2010/04/04, relative to 7 days before and 7 days after combined.**
(TIFF)

Figure S31 **Word shift graph for Mother's Day, 2010/05/09, relative to 7 days before and 7 days after combined.**
(TIFF)

Figure S32 **Word shift graph for Finale of Television Series Lost, 2010/05/24, relative to 7 days before and 7 days after combined.**
(TIFF)

Figure S33 **Word shift graph for Father's Day, 2010/06/20, relative to 7 days before and 7 days after combined.**
(TIFF)

Figure S34 **Word shift graph for Germany defeats England 4-1 in 2010 World Cup, 2010/06/27, relative to 7 days before and 7 days after combined.**
(TIFF)

Figure S35 **Word shift graph for Fourth of July, Independence Day, 2010/07/04, relative to 7 days before and 7 days after combined.**
(TIFF)

Figure S36 **Word shift graph for Thanksgiving, 2010/11/25, relative to 7 days before and 7 days after combined.**
(TIFF)

Figure S37 **Word shift graph for Christmas Eve, 2010/12/24, relative to 7 days before and 7 days after combined.**
(TIFF)

Figure S38 **Word shift graph for Christmas Day, 2010/12/25, relative to 7 days before and 7 days after combined.**
(TIFF)

Figure S39 **Word shift graph for New Year's Eve, 2010/12/31, relative to 7 days before and 7 days after combined.**
(TIFF)

Figure S40 **Word shift graph for New Year's Day, 2011/01/01, relative to 7 days before and 7 days after combined.**
(TIFF)

Figure S41 **Word shift graph for Valentine's Day, 2011/02/14, relative to 7 days before and 7 days after combined.**
(TIFF)

Figure S42
**Word shift graph for T** 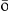 **hoku earthquake and tsunami, Japan, 2011/03/11, relative to 7 days before and 7 days after combined.**
(TIFF)

Figure S43 **Word shift graph for Easter, 2011/04/24, relative to 7 days before and 7 days after combined.**
(TIFF)

Figure S44 **Word shift graph for Royal Wedding of Prince William & Catherine Middleton, 2011/04/29, relative to 7 days before and 7 days after combined.**
(TIFF)

Figure S45 **Word shift graph for Death of Osama Bin Laden, 2011/05/02, relative to 7 days before and 7 days after combined.**
(TIFF)

Figure S46 **Word shift graph for Mother's Day, 2011/05/08, relative to 7 days before and 7 days after combined.**
(TIFF)

Figure S47 **Word shift graph for Father's Day, 2011/06/19, relative to 7 days before and 7 days after combined.**
(TIFF)

Figure S48 **Word shift graph for News of the Week Hacking Scandal, 2011/07/05, relative to 7 days before and 7 days after combined.**
(TIFF)

Figure S49 **Word shift graph for Norway attacks and Amy Winehouse's death, 2011/07/23, relative to 7 days before and 7 days after combined.**
(TIFF)

Figure S50 **Word shift graph for London riots, 2011/08/08, relative to 7 days before and 7 days after combined.**
(TIFF)

Figure S51 **Word shift graph for Virginia earthquake, 2011/08/23, relative to 7 days before and 7 days after combined.**
(TIFF)

Figure S52 **Word shift graph for 10th anniversay of 9/11, 2011/09/11, relative to 7 days before and 7 days after combined.**
(TIFF)

Table S1
**The same selection of 100 keywords and text elements listed in the main text's** **Table 2** **, reordered by normalized happiness** 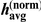 **.**
(PDF)
