# Supplementary figures and images for: Temporal Patterns of Happiness and Information in a Global Social Network: Hedonometrics and Twitter
Source: PLoS One. 2011 Dec 7;6(12):e26752. doi: 10.1371/journal.pone.0026752 (PMC3233600; doi:10.1371/journal.pone.0026752)

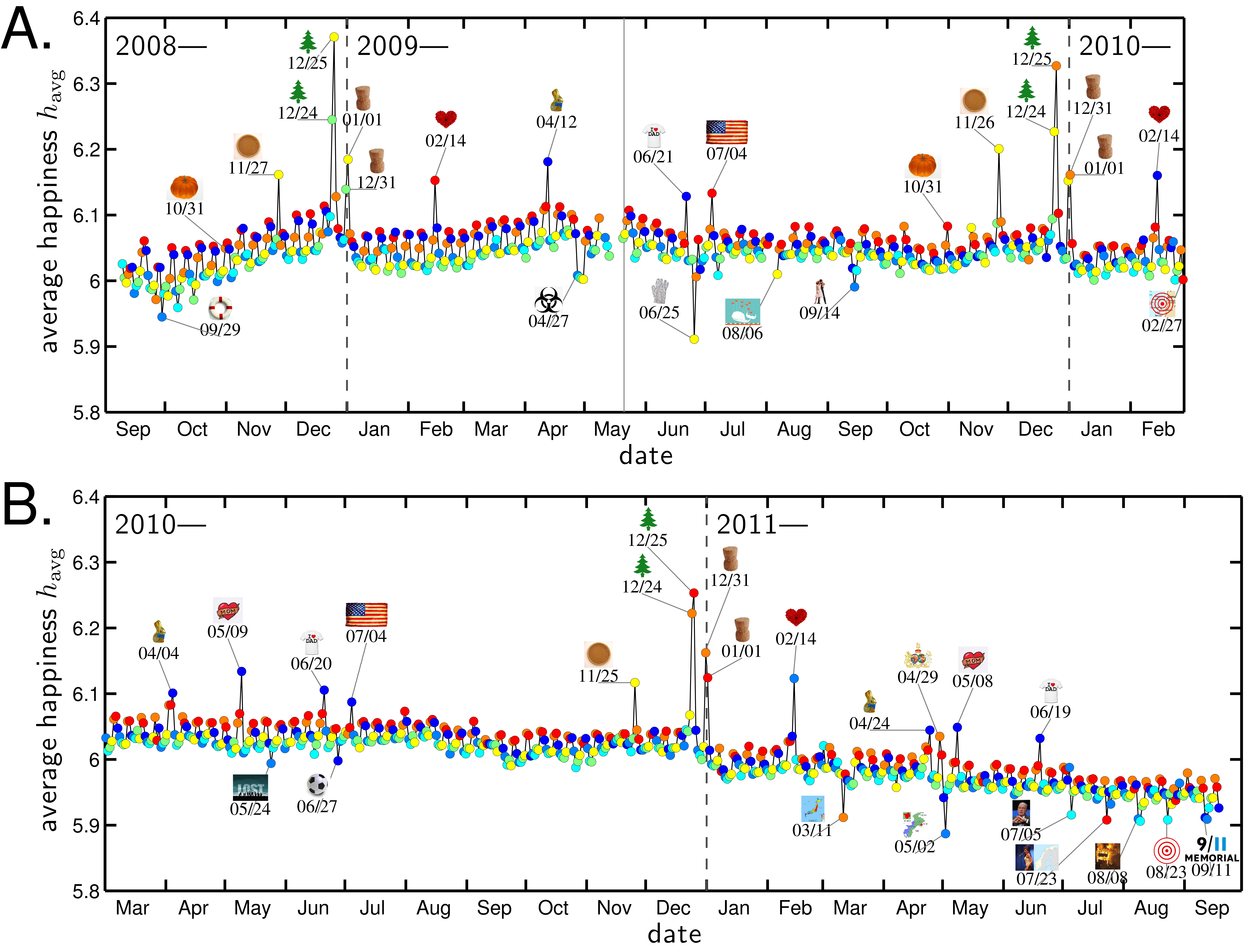

Supplement: Figure S2 — Simple average happiness time series plots. The time series is extended to include part of September, 2011, and shows a drop corresponding to the tenth anniversary of the 9/11 terror attacks in the United States. (TIFF) [file pone.0026752.s003.tif]

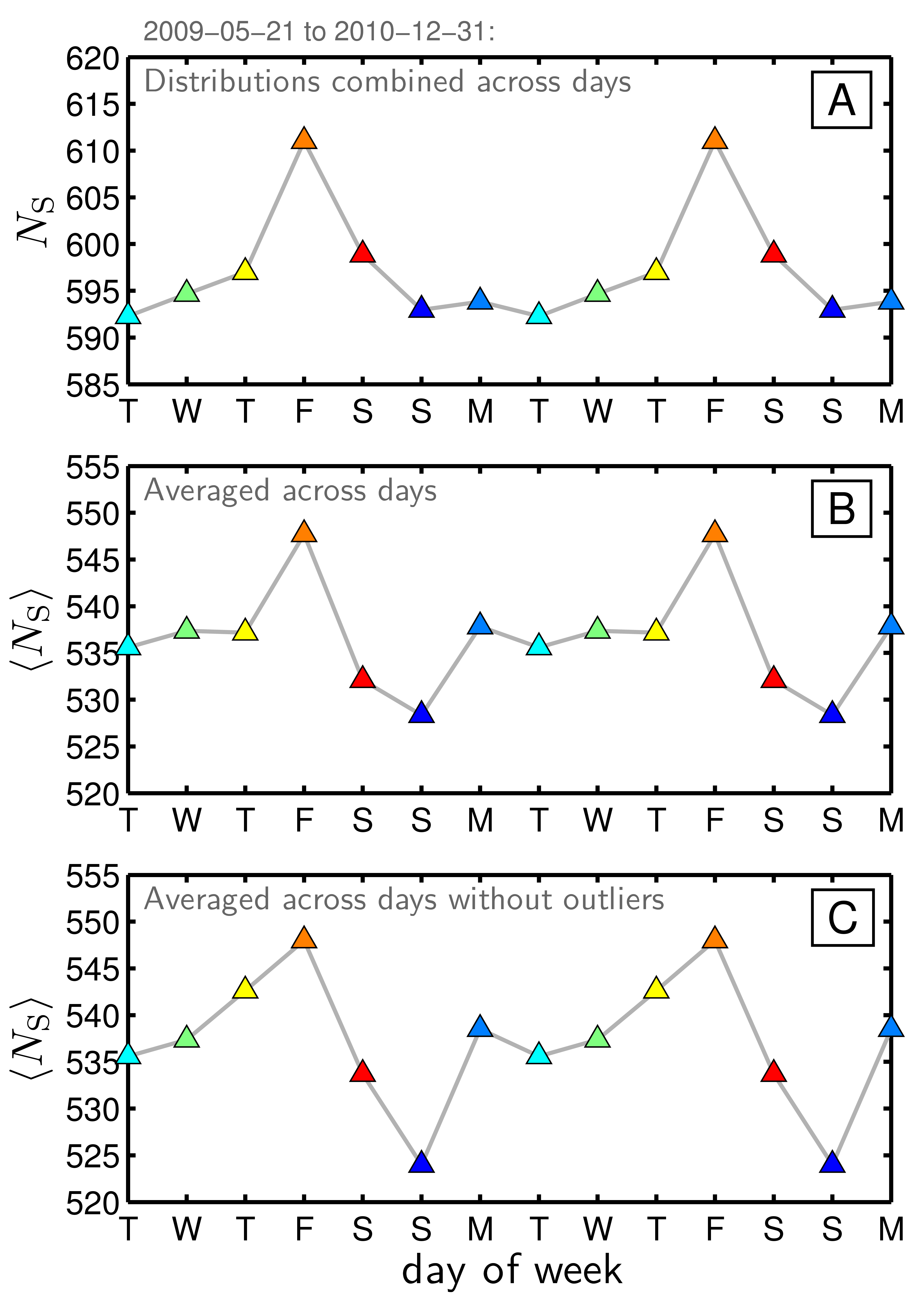

Supplement: Figure S3 — Simpson lexical size as a function of day of the week using three different ways of creating distributions. Compare with Fig. 9. (TIFF) [file pone.0026752.s004.tif]

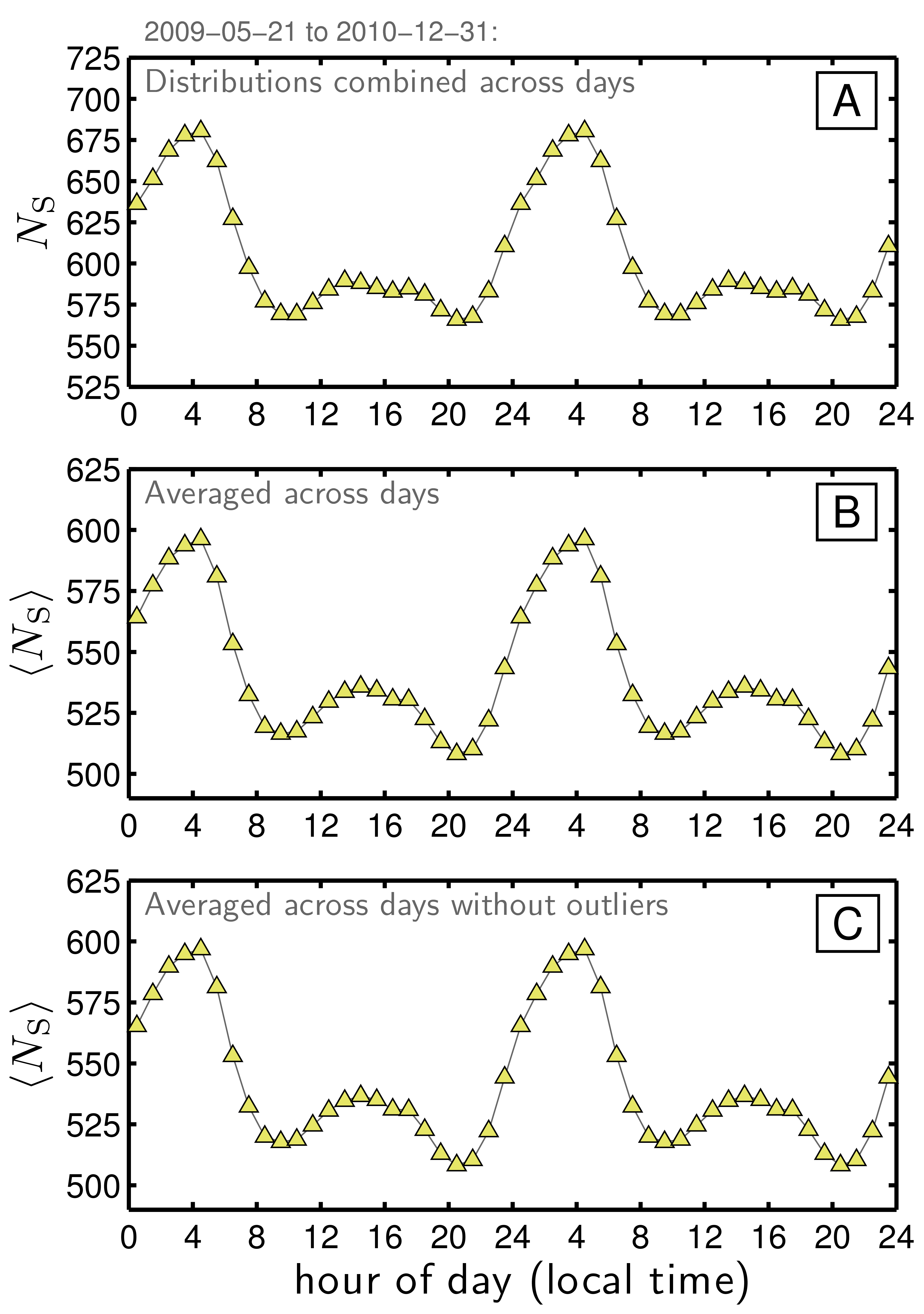

Supplement: Figure S4 — Average Simpson lexical size for time of day, corrected according to local time, using three different ways of creating distributions. Compare with Fig. 13. (TIFF) [file pone.0026752.s005.tif]

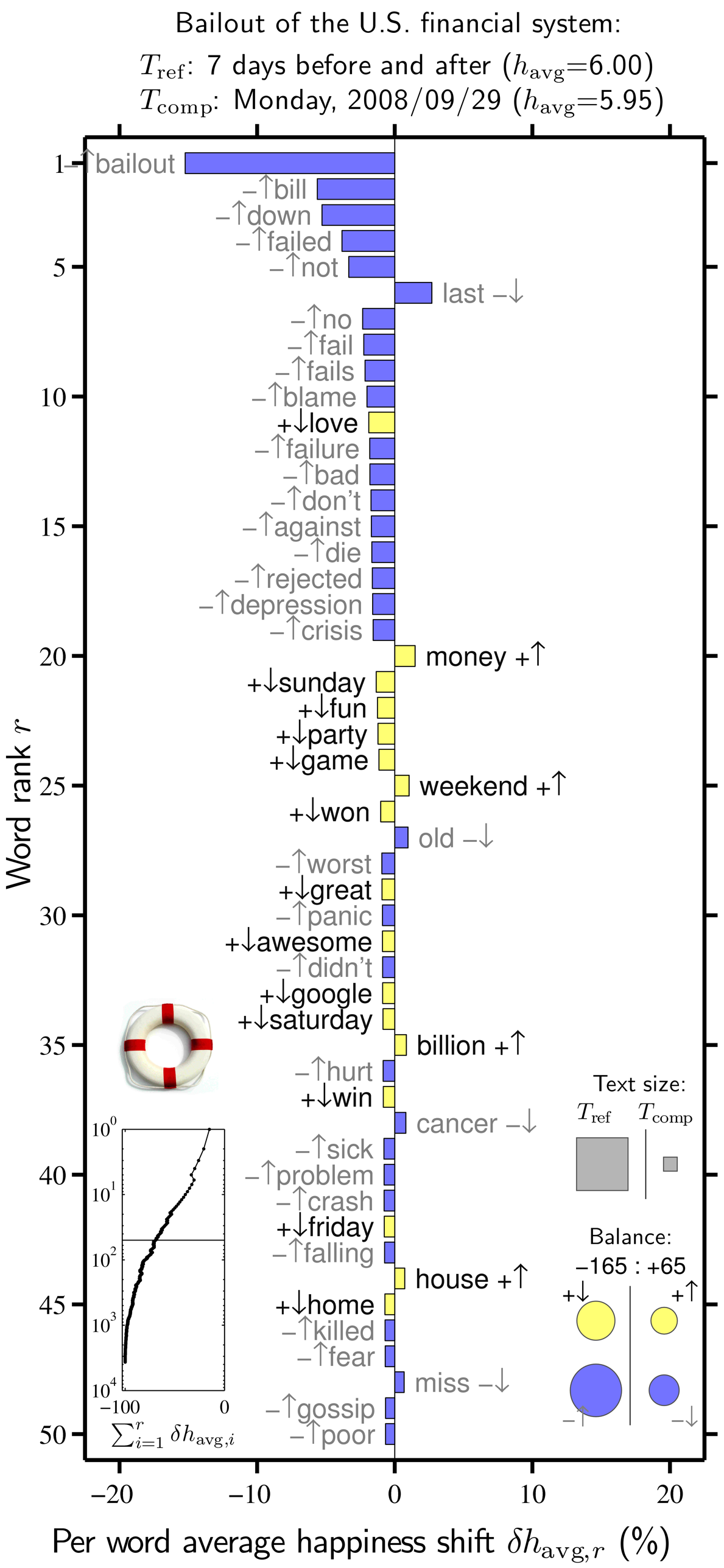

Supplement: Figure S7 — Word shift graph for Bailout of the U.S. financial system, 2008/09/29, relative to 7 days before and 7 days after combined. (TIFF) [file pone.0026752.s008.tif]

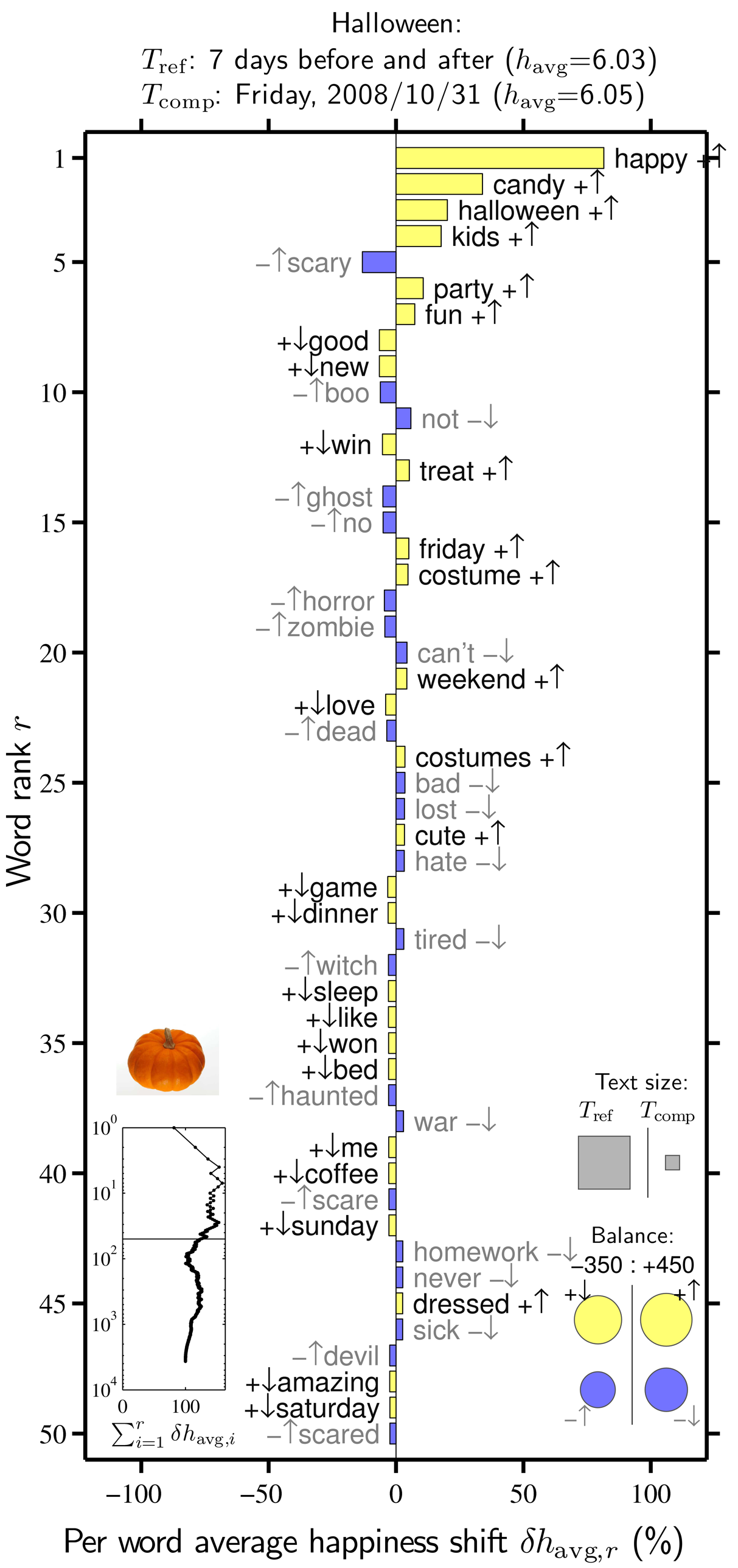

Supplement: Figure S8 — Word shift graph for Halloween, 2008/10/31, relative to 7 days before and 7 days after combined. (TIFF) [file pone.0026752.s009.tif]

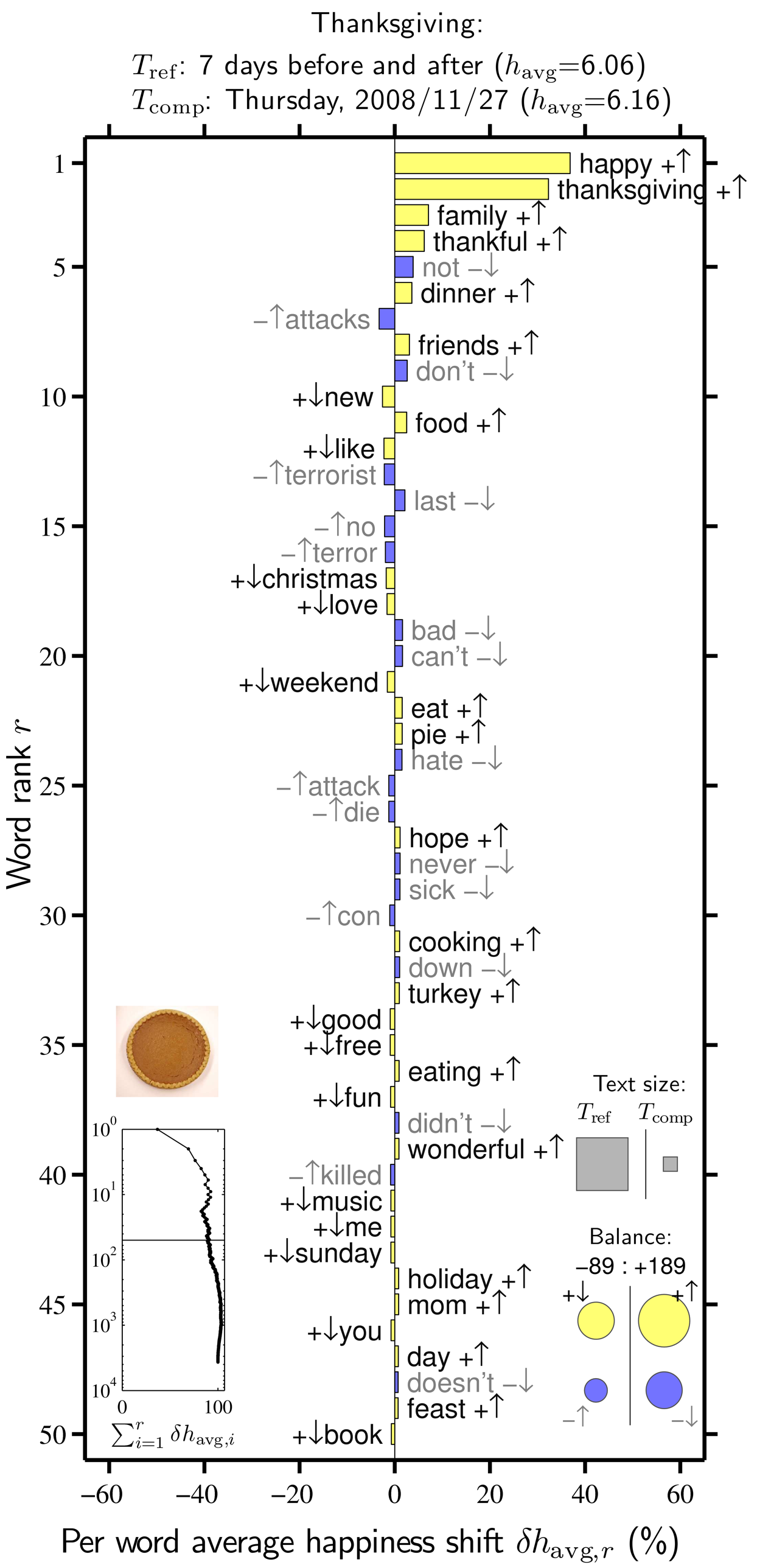

Supplement: Figure S9 — Word shift graph for Thanksgiving, 2008/11/27, relative to 7 days before and 7 days after combined. (TIFF) [file pone.0026752.s010.tif]

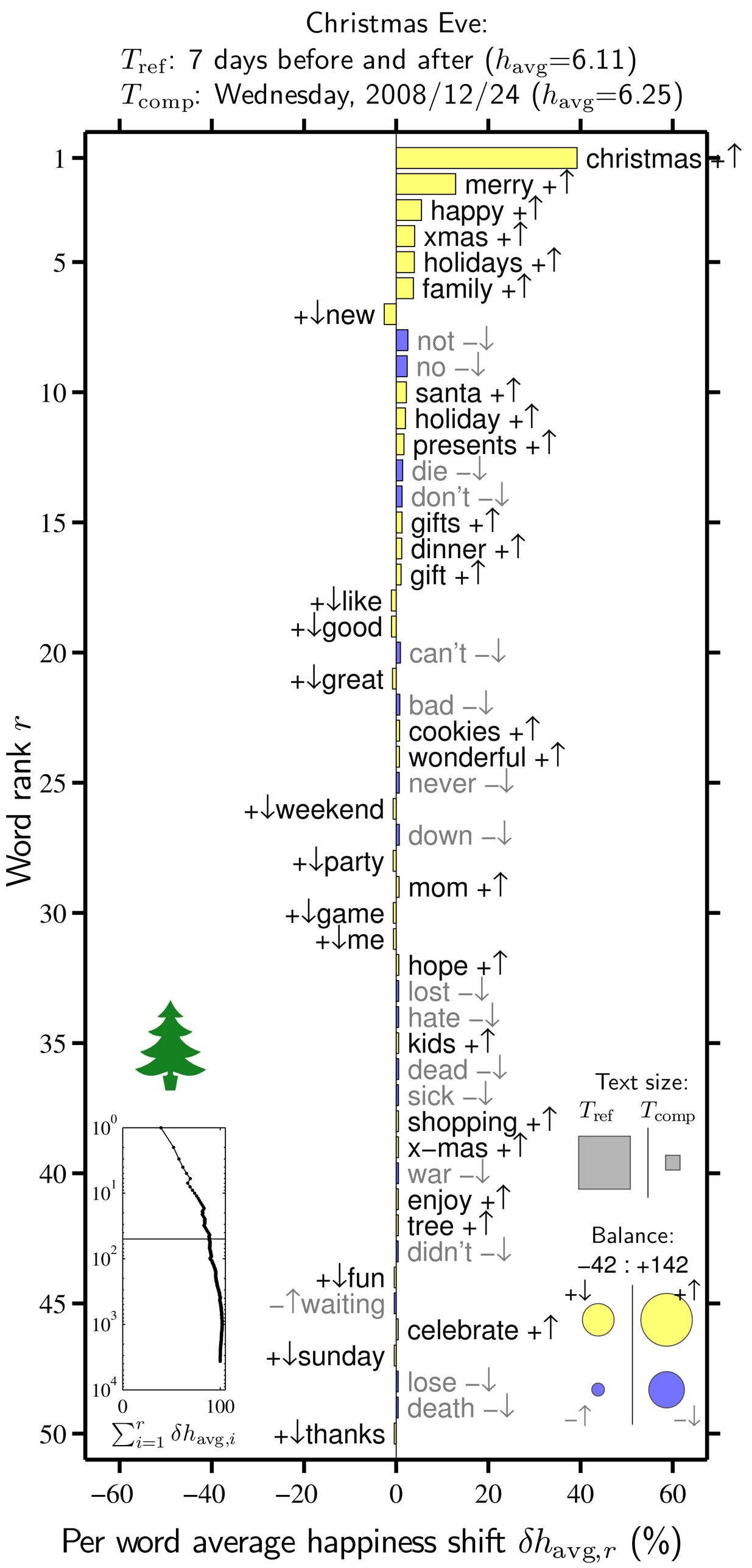

Supplement: Figure S10 — Word shift graph for Christmas Eve, 2008/12/24, relative to 7 days before and 7 days after combined. (TIFF) [file pone.0026752.s011.tif]

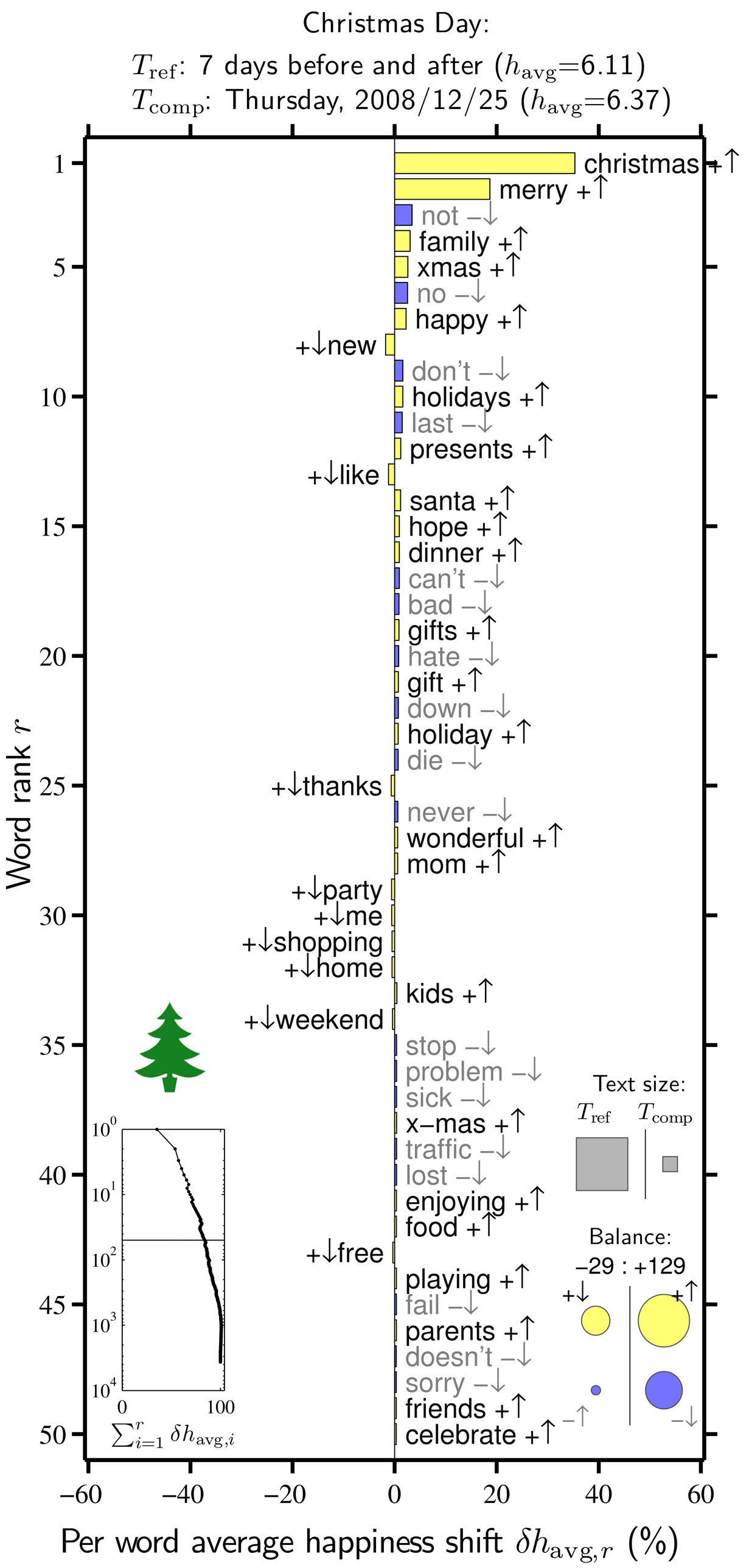

Supplement: Figure S11 — Word shift graph for Christmas Day, 2008/12/25, relative to 7 days before and 7 days after combined. (TIFF) [file pone.0026752.s012.tif]

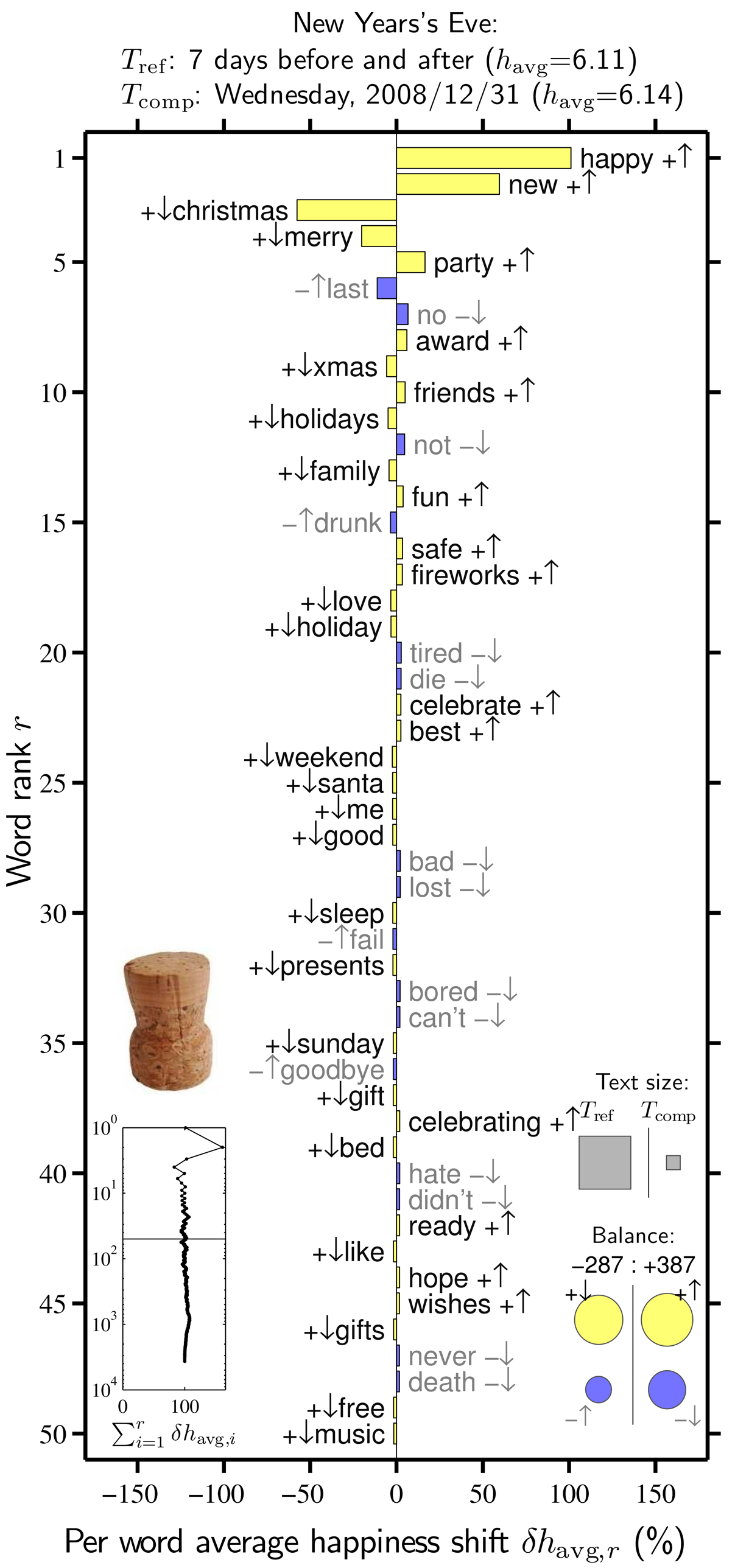

Supplement: Figure S12 — Word shift graph for New Years's Eve, 2008/12/31, relative to 7 days before and 7 days after combined. (TIFF) [file pone.0026752.s013.tif]

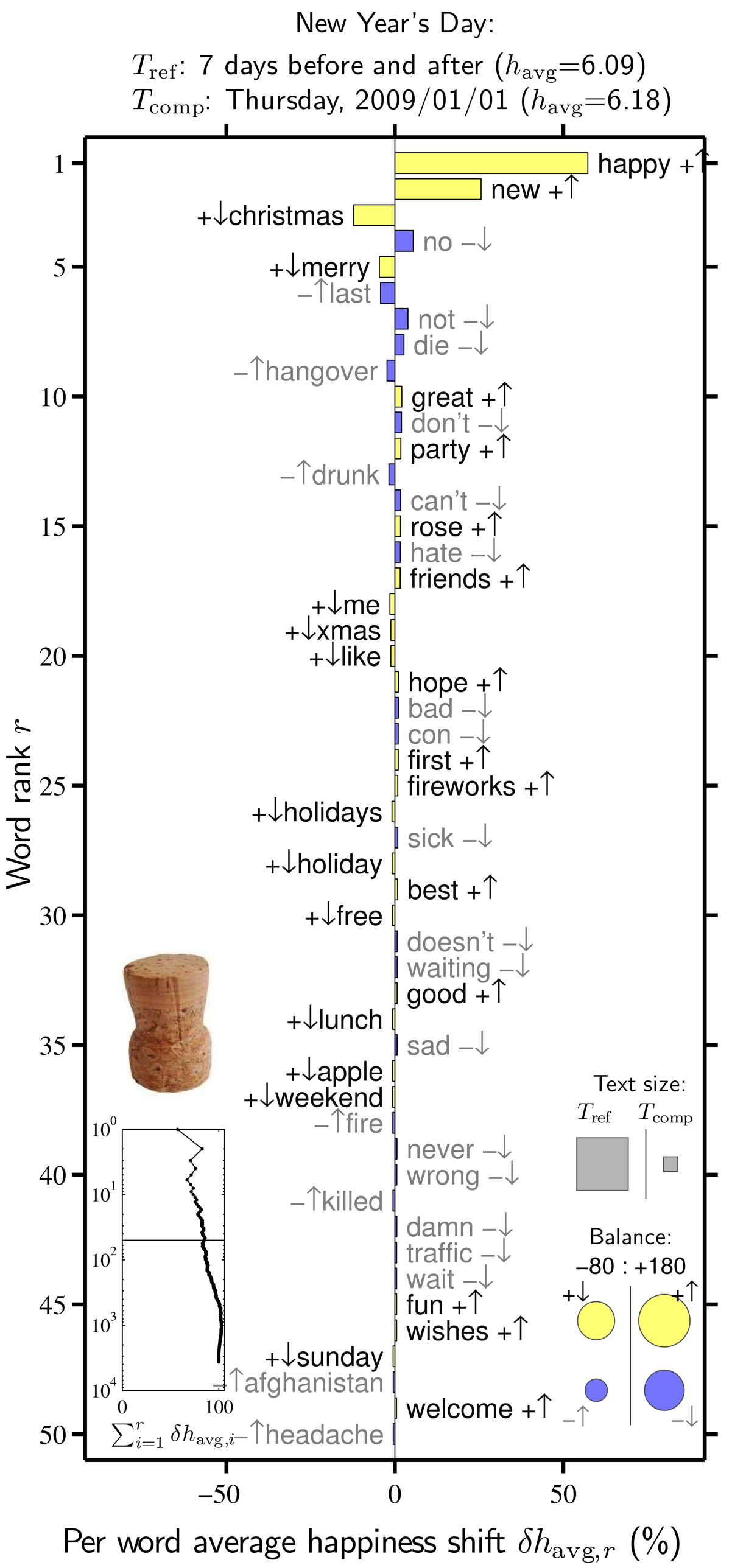

Supplement: Figure S13 — Word shift graph for New Year's Day, 2009/01/01, relative to 7 days before and 7 days after combined. (TIFF) [file pone.0026752.s014.tif]

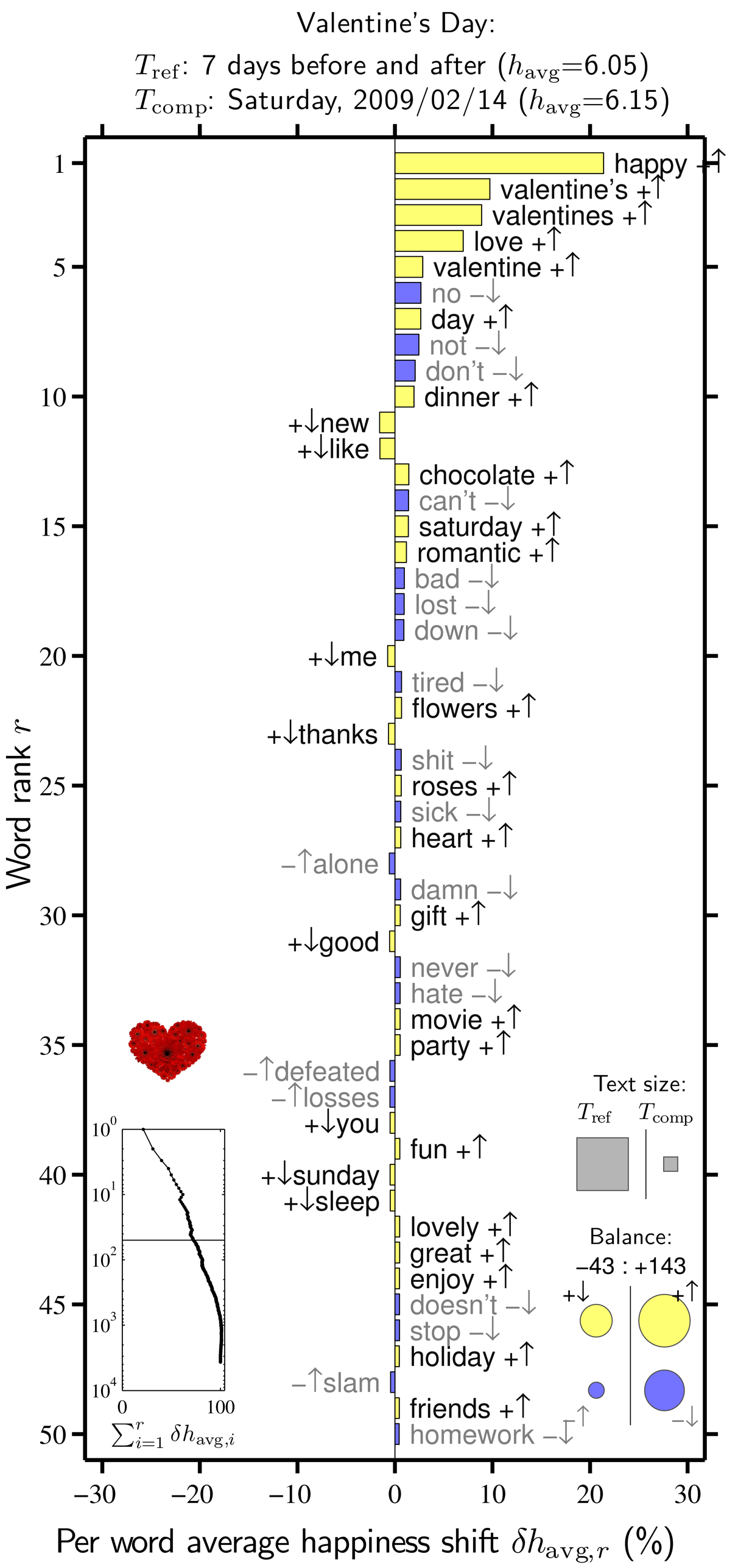

Supplement: Figure S14 — Word shift graph for Valentine's Day, 2009/02/14, relative to 7 days before and 7 days after combined. (TIFF) [file pone.0026752.s015.tif]

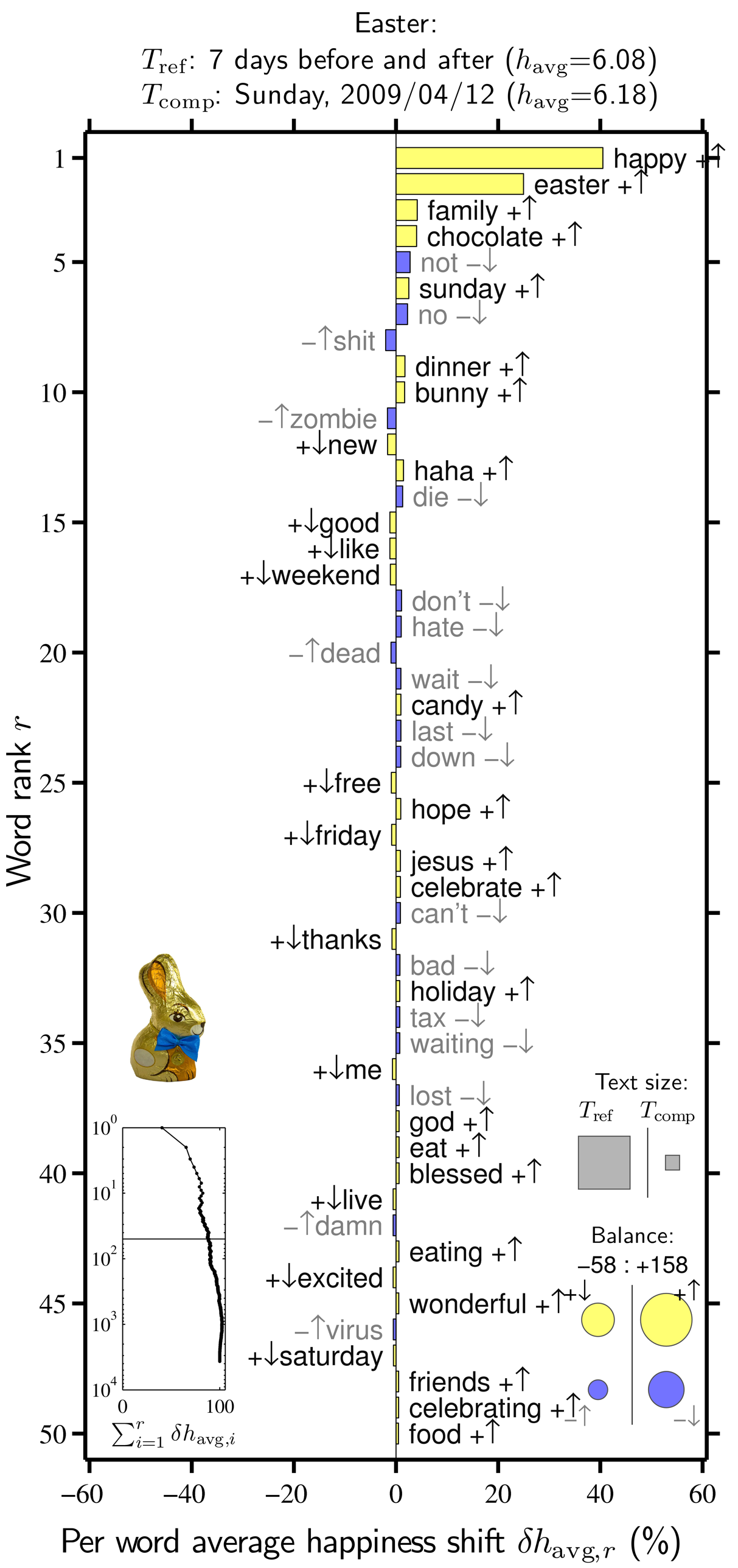

Supplement: Figure S15 — Word shift graph for Easter, 2009/04/12, relative to 7 days before and 7 days after combined. (TIFF) [file pone.0026752.s016.tif]

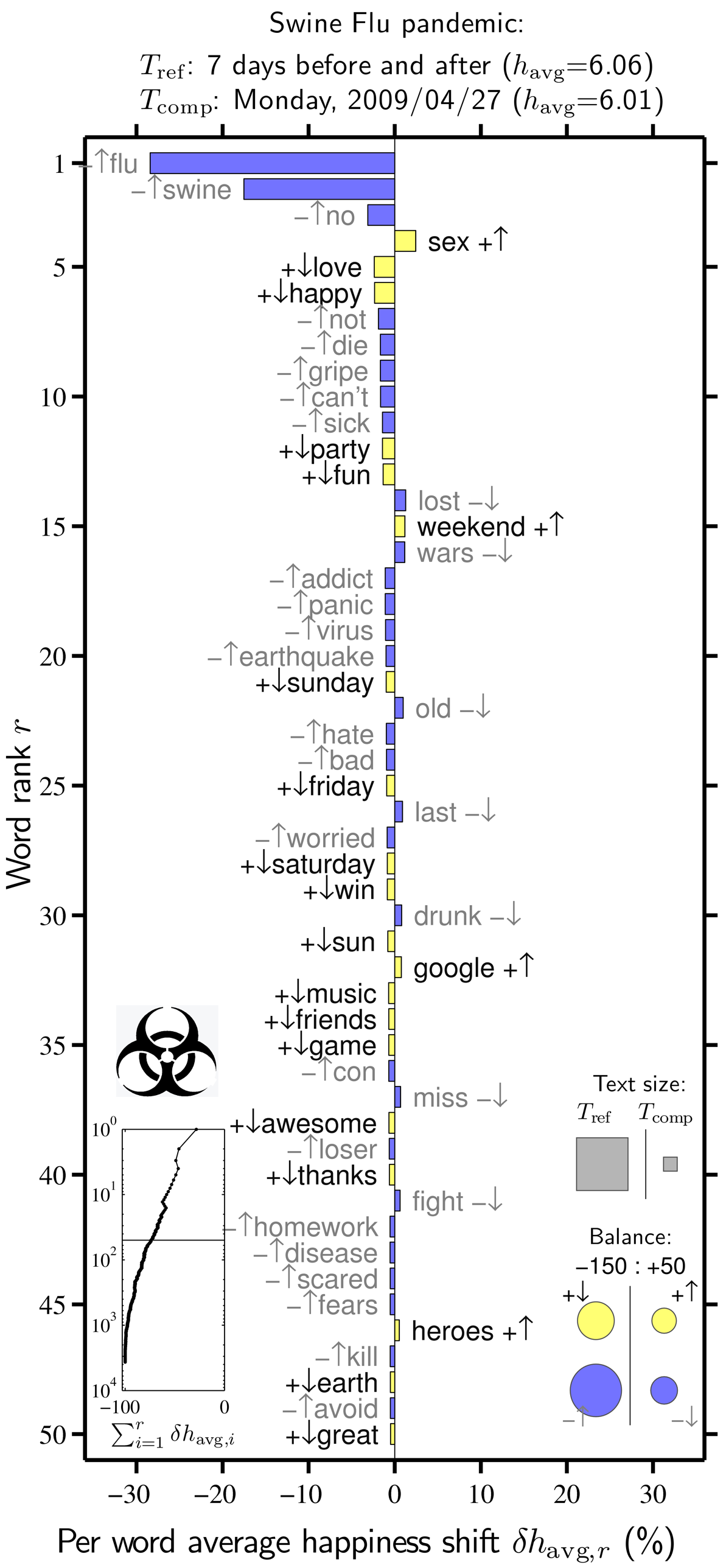

Supplement: Figure S16 — Word shift graph for Swine Flu pandemic, 2009/04/27, relative to 7 days before and 7 days after combined. (TIFF) [file pone.0026752.s017.tif]

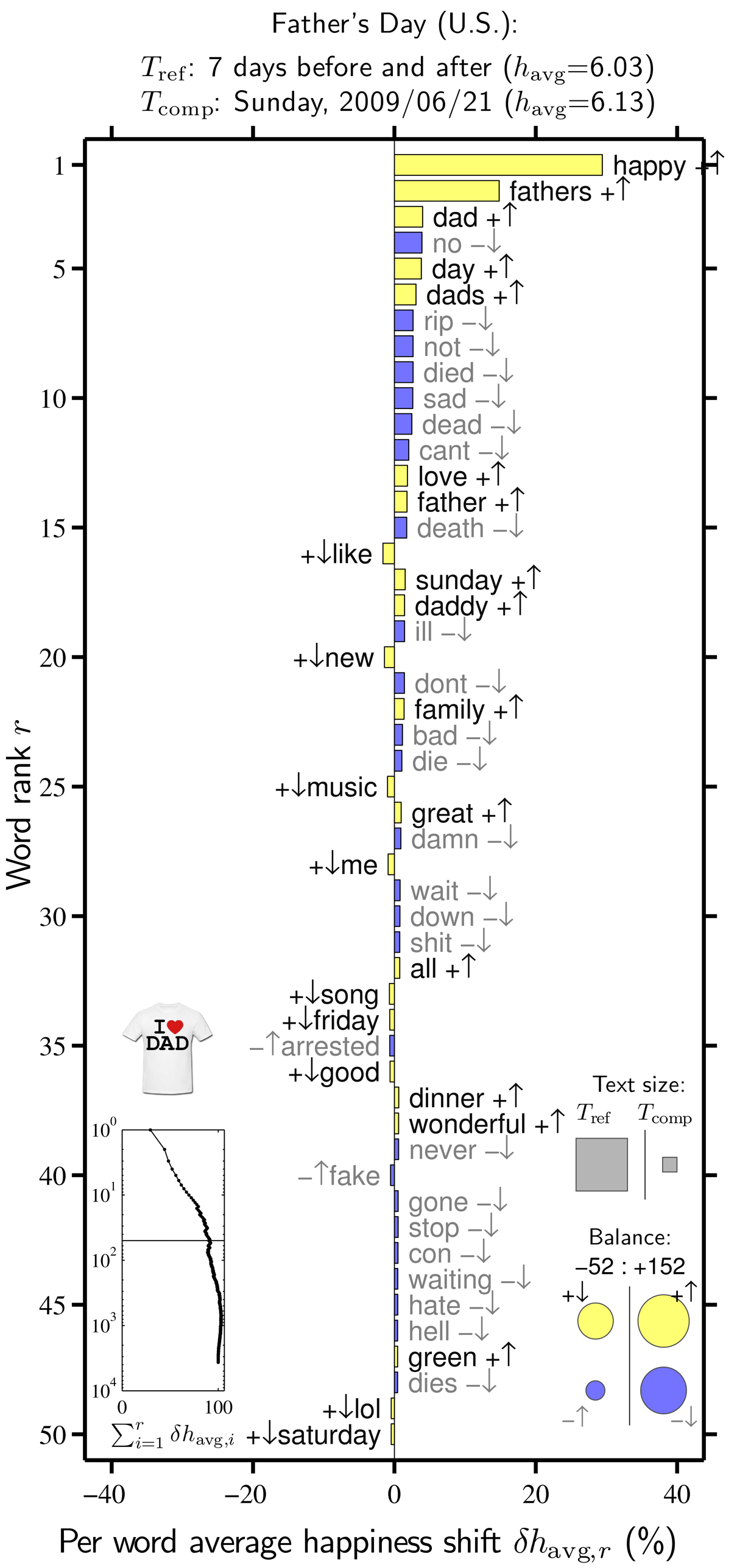

Supplement: Figure S17 — Word shift graph for Father's Day (U.S.), 2009/06/21, relative to 7 days before and 7 days after combined. (TIFF) [file pone.0026752.s018.tif]

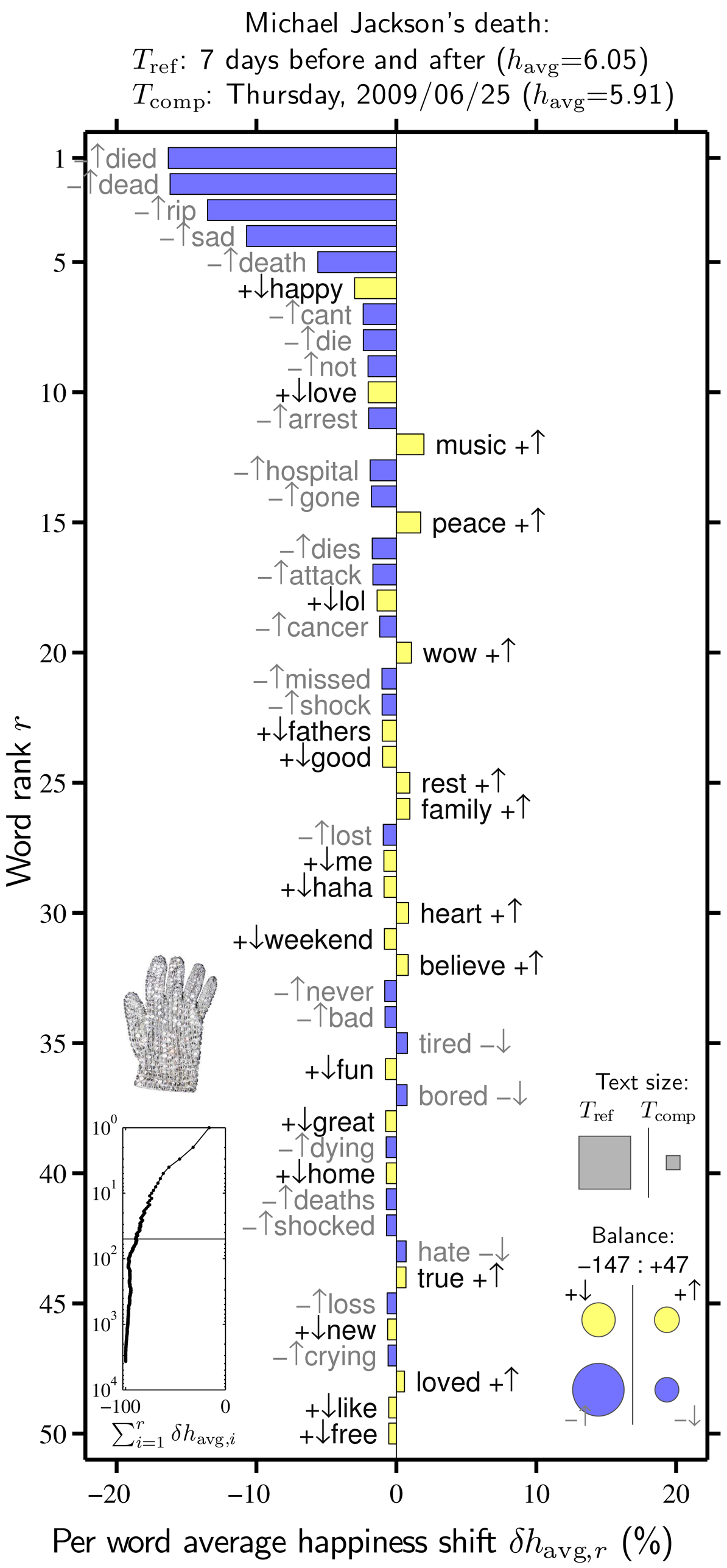

Supplement: Figure S18 — Word shift graph for Michael Jackson's death, 2009/06/25, relative to 7 days before and 7 days after combined. (TIFF) [file pone.0026752.s019.tif]

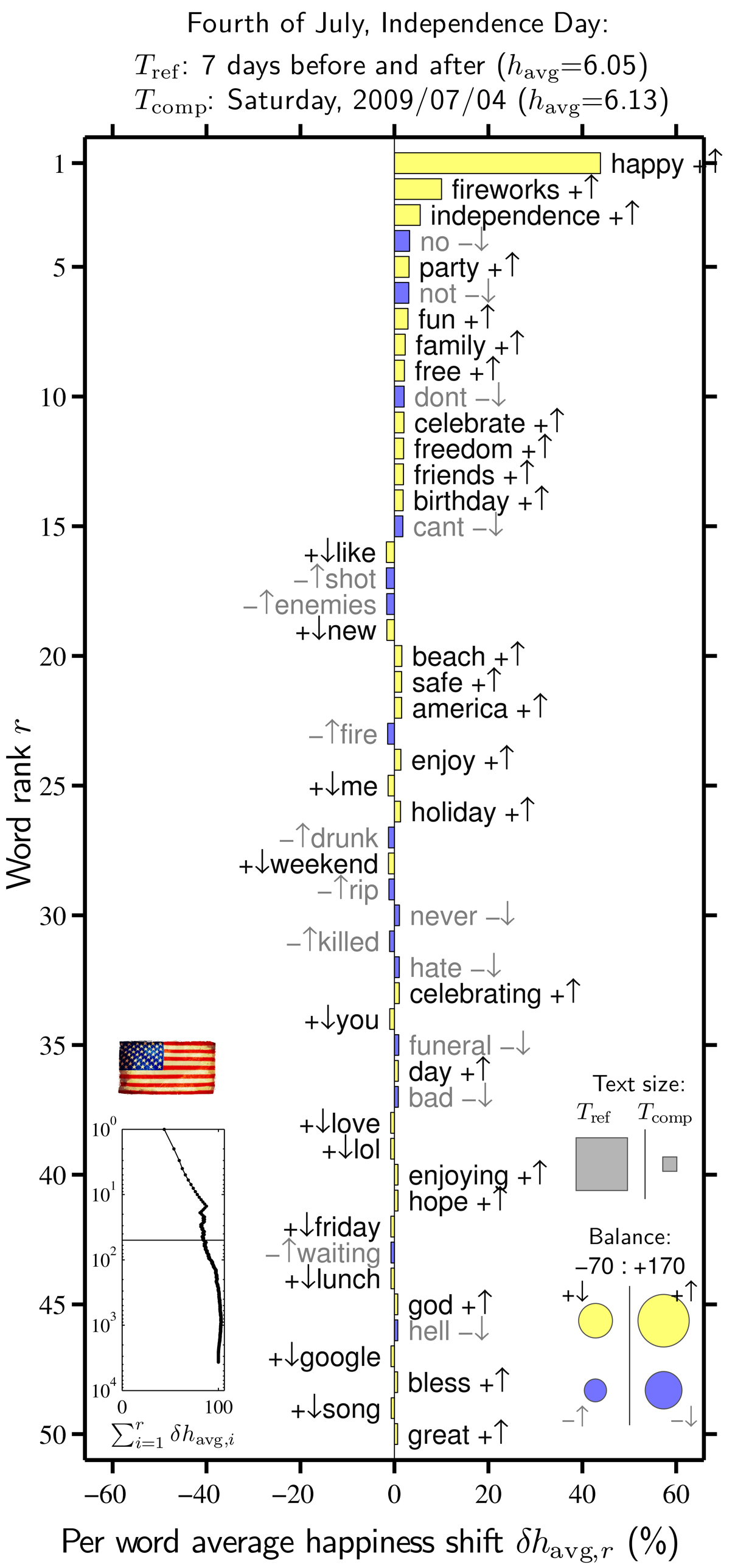

Supplement: Figure S19 — Word shift graph for Fourth of July, Independence Day, 2009/07/04, relative to 7 days before and 7 days after combined. (TIFF) [file pone.0026752.s020.tif]

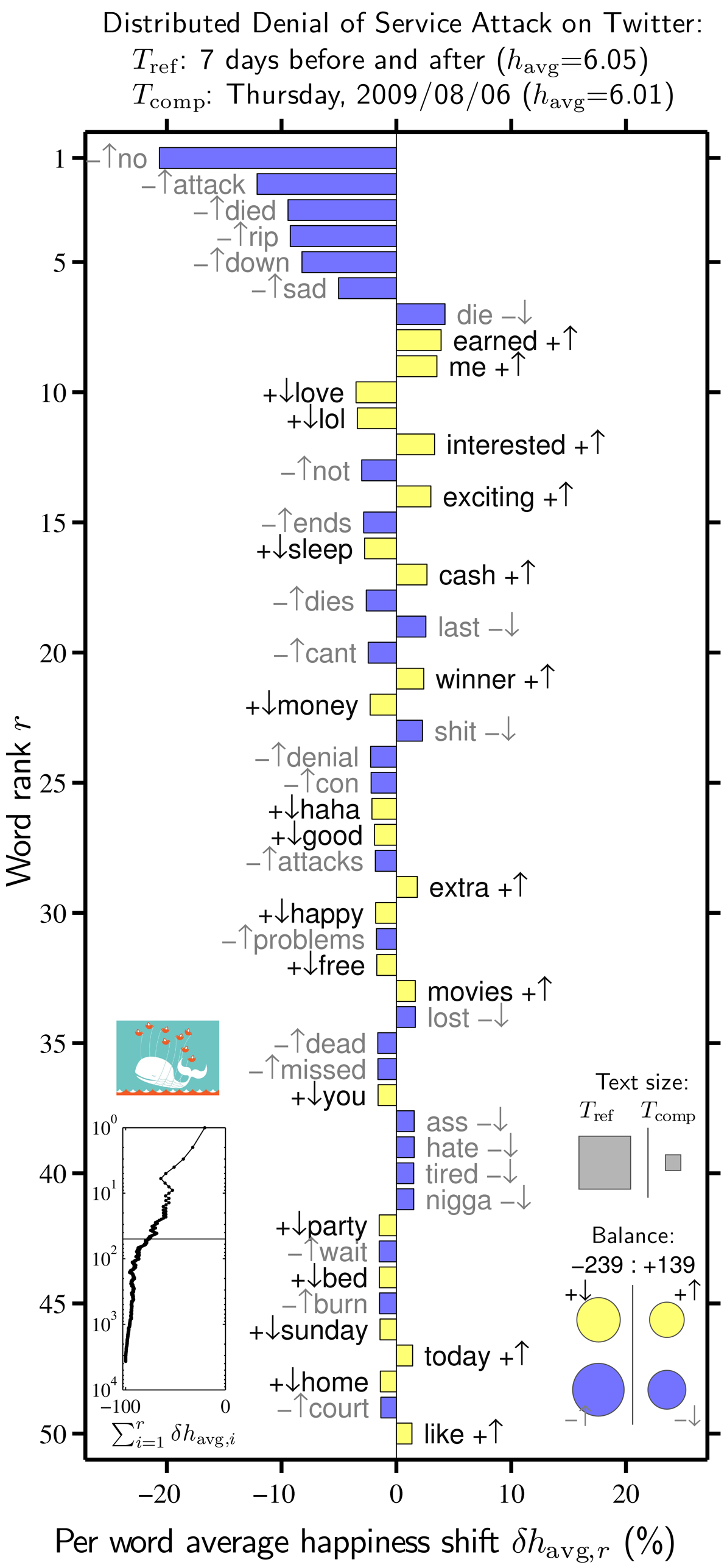

Supplement: Figure S20 — Word shift graph for Distributed Denial of Service Attack on Twitter, 2009/08/06, relative to 7 days before and 7 days after combined. (TIFF) [file pone.0026752.s021.tif]

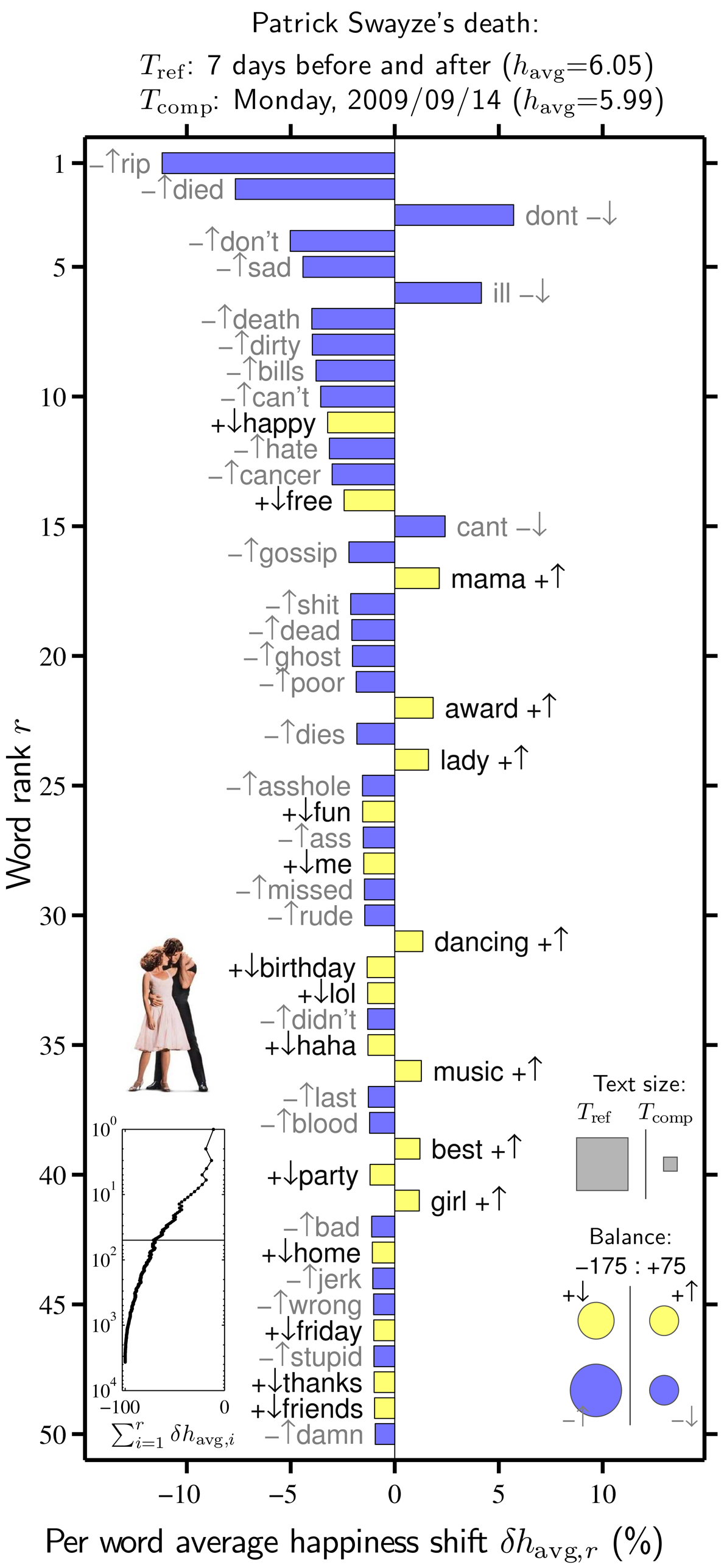

Supplement: Figure S21 — Word shift graph for Patrick Swayze's death, 2009/09/14, relative to 7 days before and 7 days after combined. (TIFF) [file pone.0026752.s022.tif]

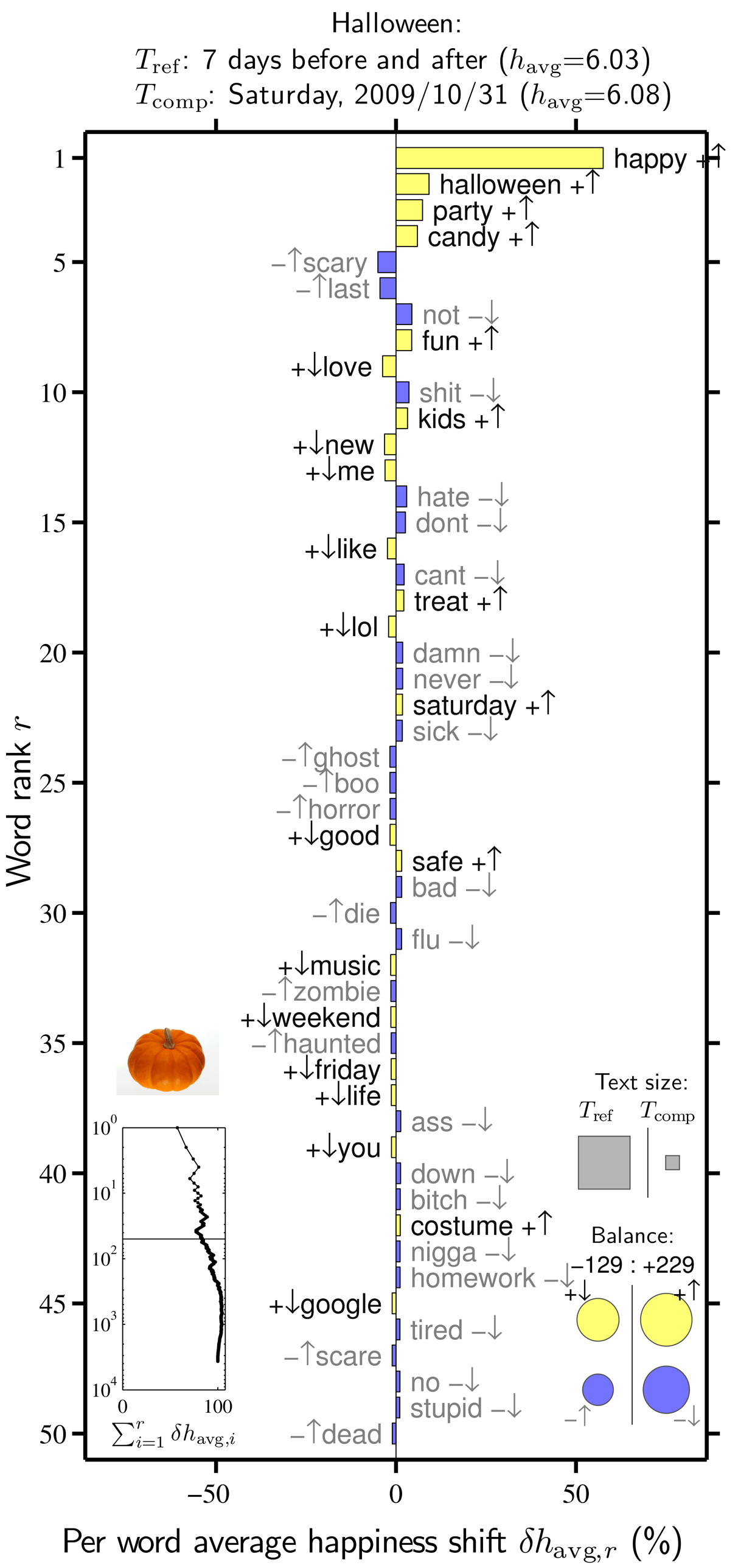

Supplement: Figure S22 — Word shift graph for Halloween, 2009/10/31, relative to 7 days before and 7 days after combined. (TIFF) [file pone.0026752.s023.tif]

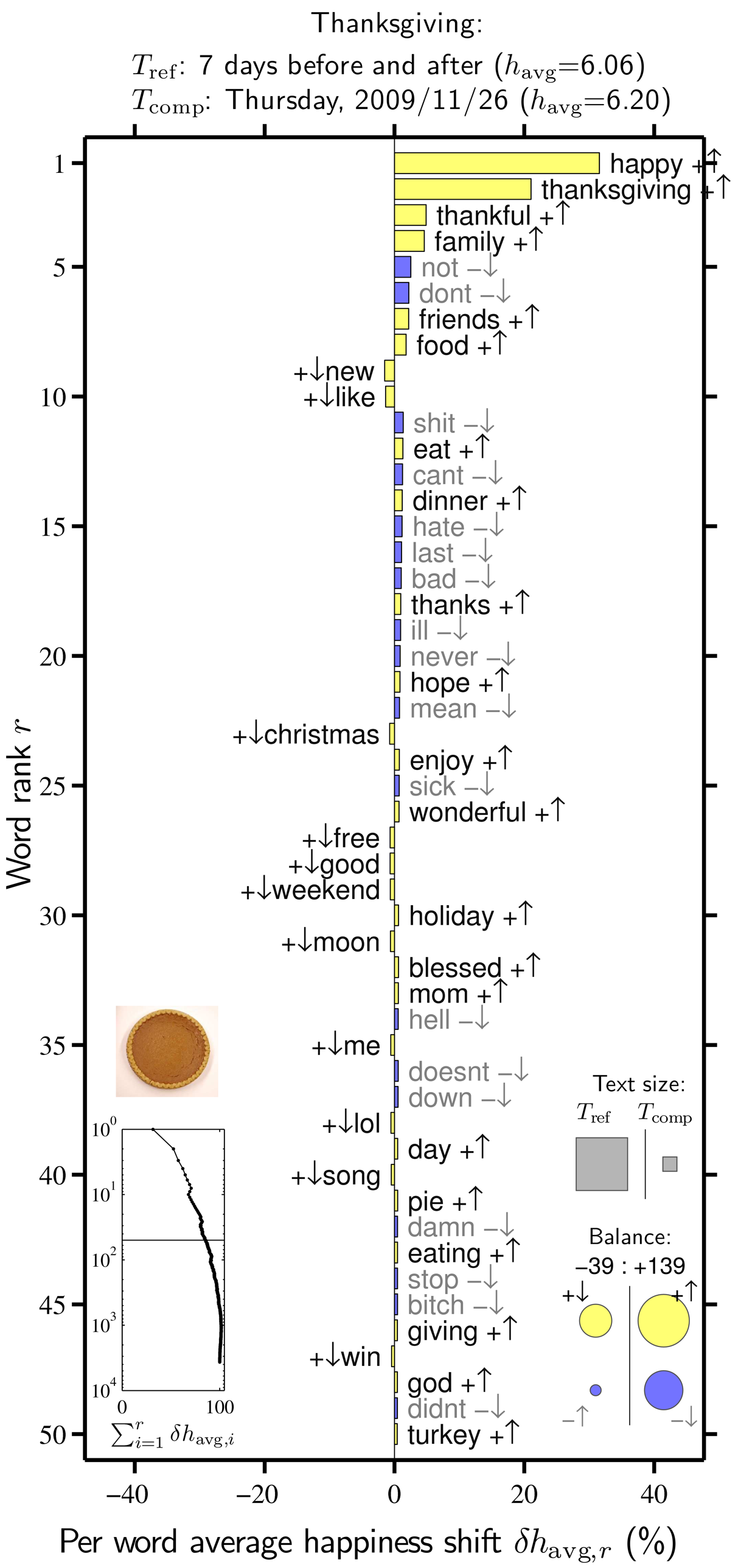

Supplement: Figure S23 — Word shift graph for Thanksgiving, 2009/11/26, relative to 7 days before and 7 days after combined. (TIFF) [file pone.0026752.s024.tif]

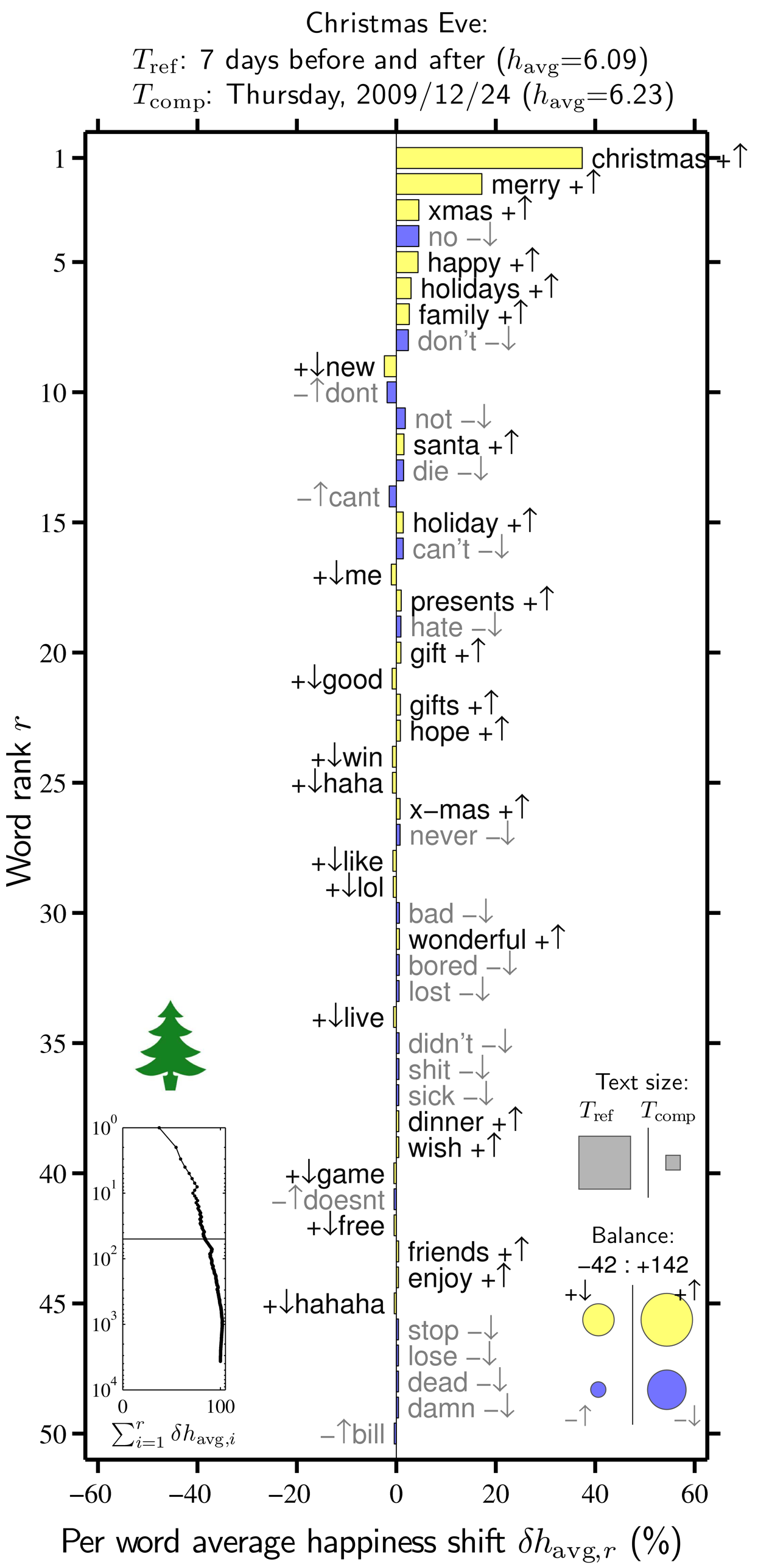

Supplement: Figure S24 — Word shift graph for Christmas Eve, 2009/12/24, relative to 7 days before and 7 days after combined. (TIFF) [file pone.0026752.s025.tif]

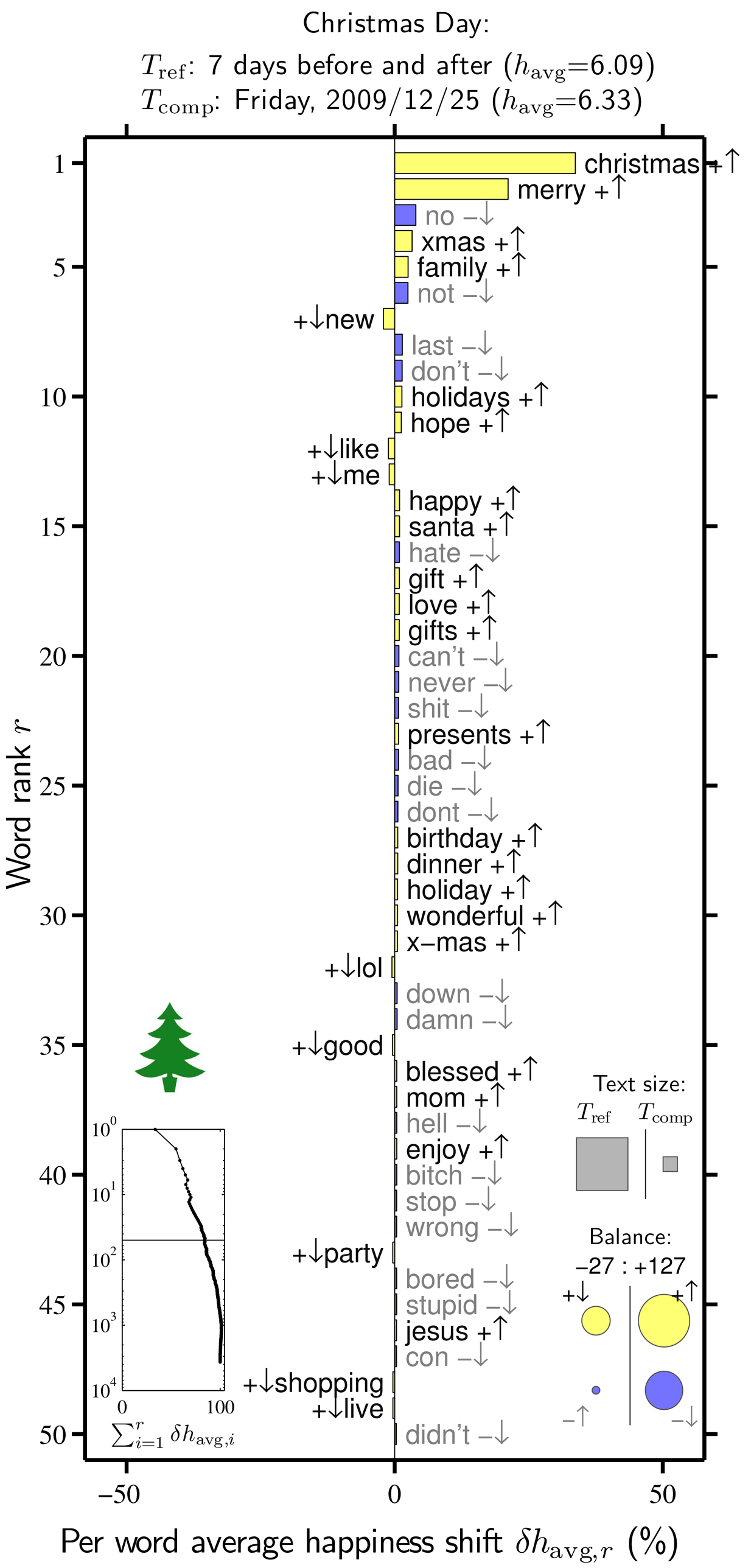

Supplement: Figure S25 — Word shift graph for Christmas Day, 2009/12/25, relative to 7 days before and 7 days after combined. (TIFF) [file pone.0026752.s026.tif]

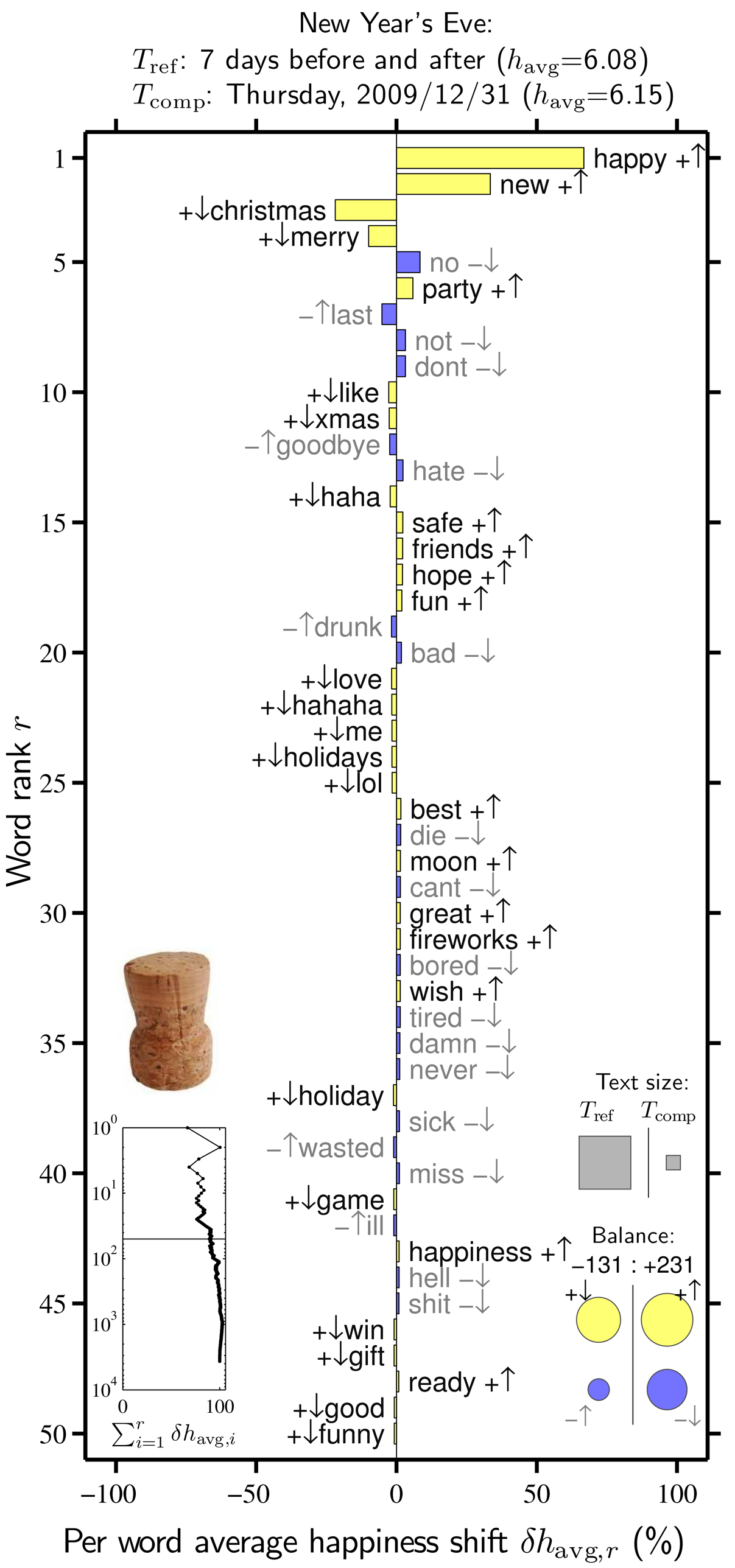

Supplement: Figure S26 — Word shift graph for New Year's Eve, 2009/12/31, relative to 7 days before and 7 days after combined. (TIFF) [file pone.0026752.s027.tif]

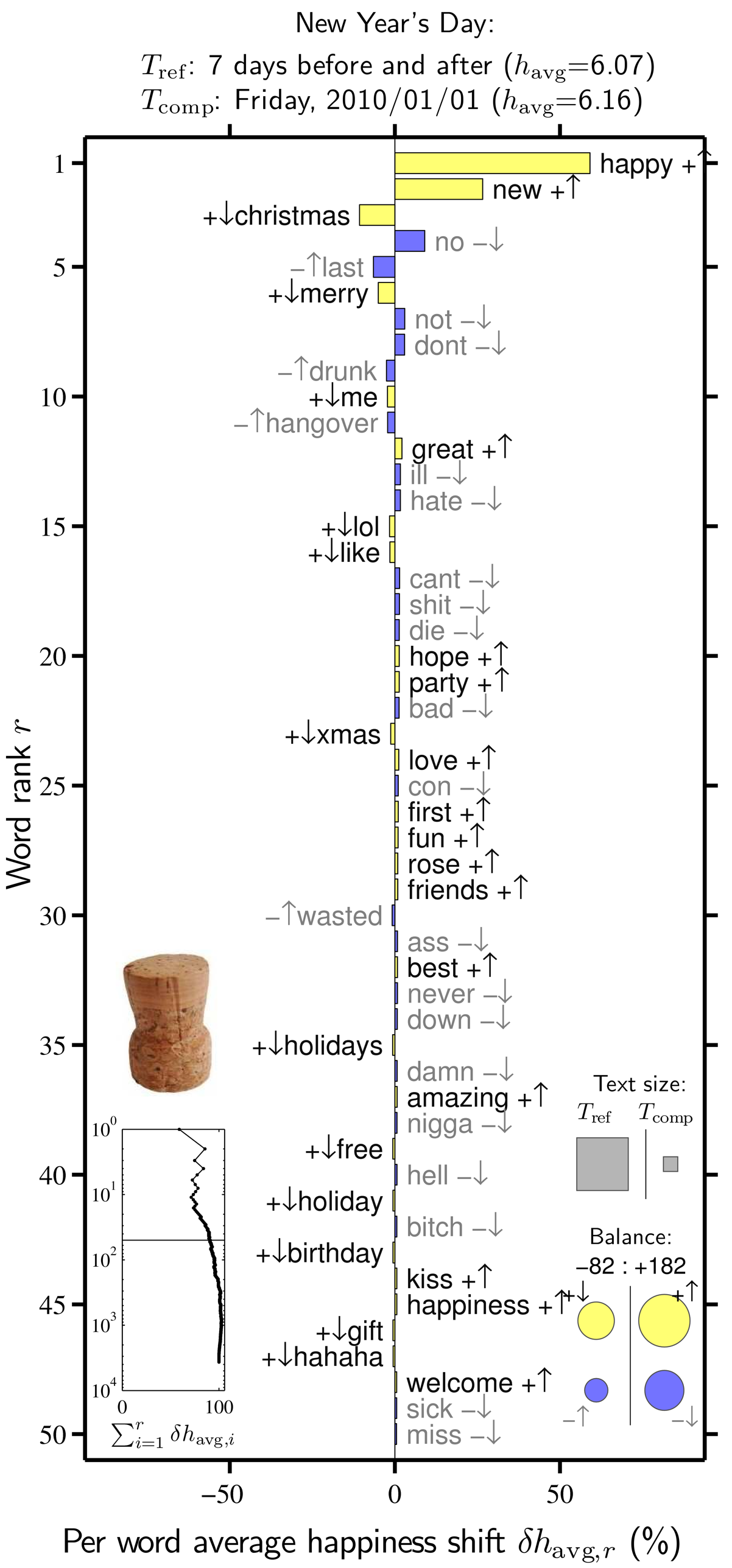

Supplement: Figure S27 — Word shift graph for New Year's Day, 2010/01/01, relative to 7 days before and 7 days after combined. (TIFF) [file pone.0026752.s028.tif]

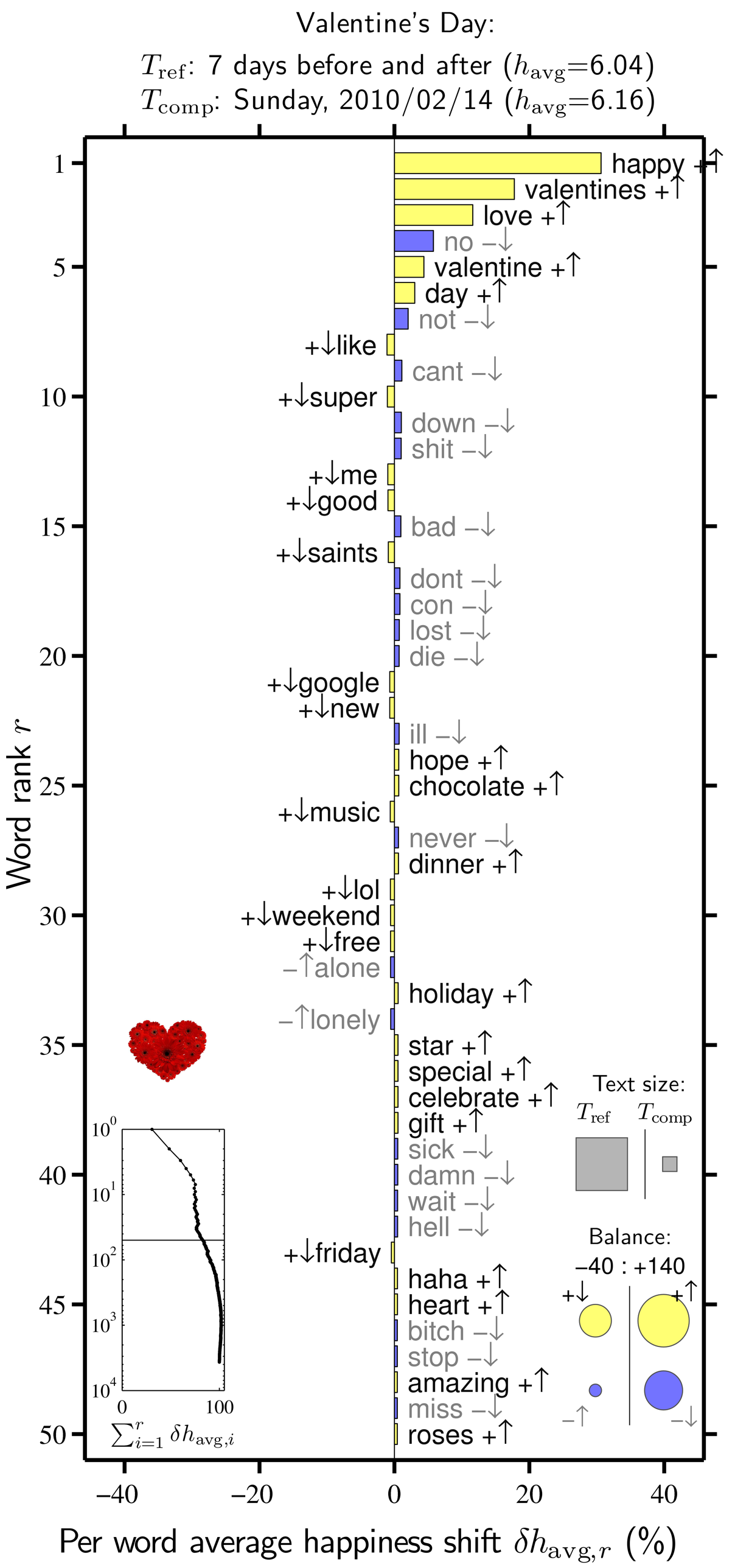

Supplement: Figure S28 — Word shift graph for Valentine's Day, 2010/02/14, relative to 7 days before and 7 days after combined. (TIFF) [file pone.0026752.s029.tif]

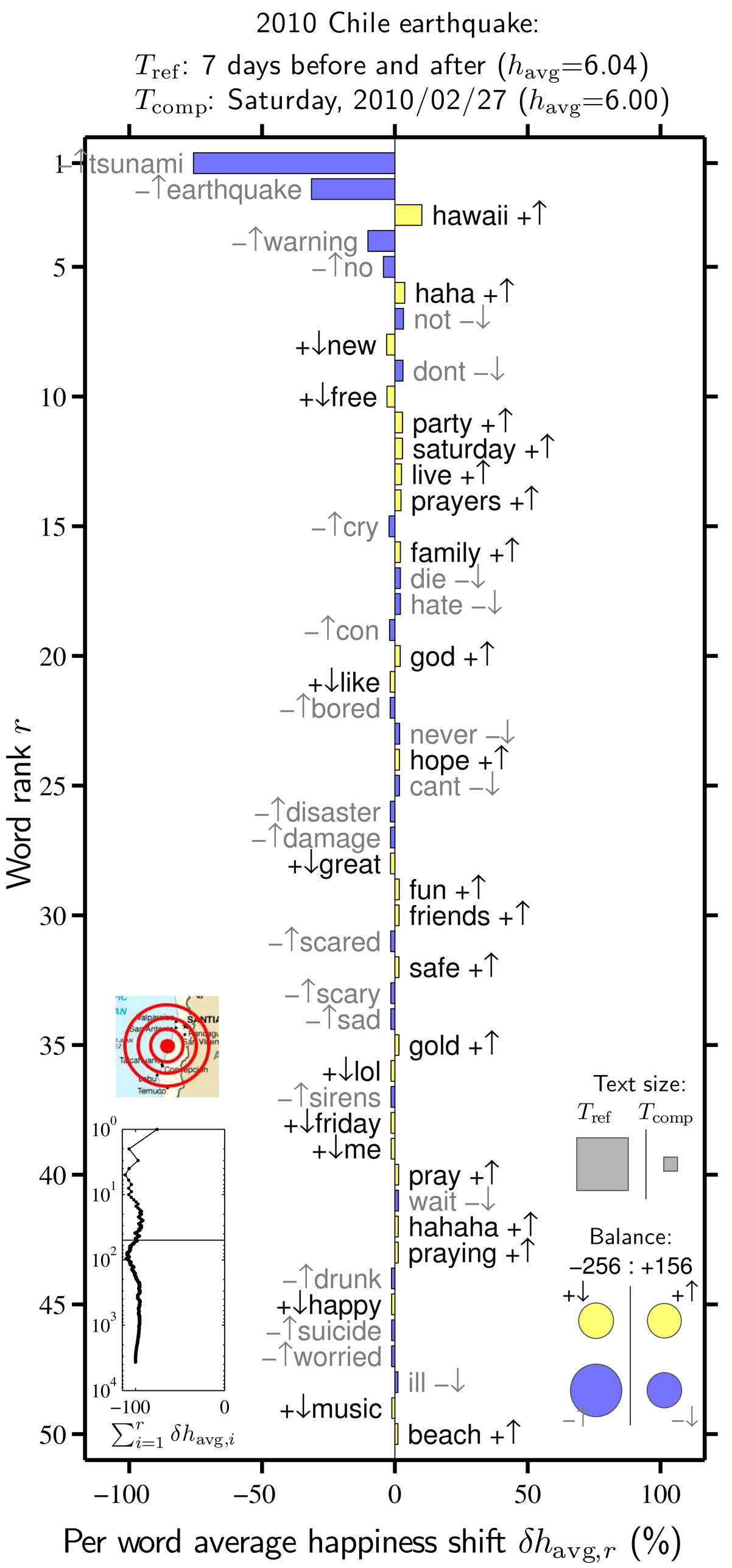

Supplement: Figure S29 — Word shift graph for 2010 Chile earthquake, 2010/02/27, relative to 7 days before and 7 days after combined. (TIFF) [file pone.0026752.s030.tif]

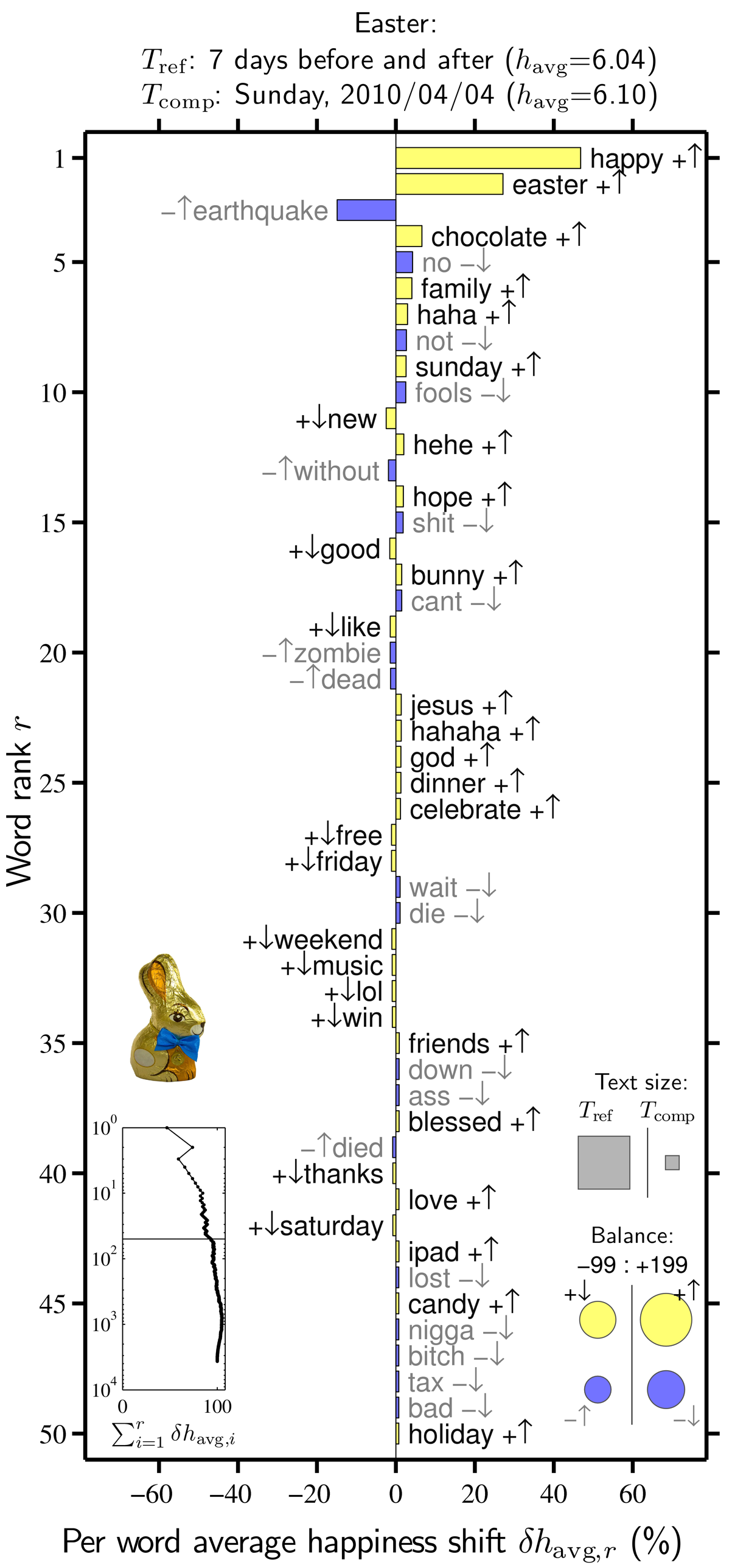

Supplement: Figure S30 — Word shift graph for Easter, 2010/04/04, relative to 7 days before and 7 days after combined. (TIFF) [file pone.0026752.s031.tif]

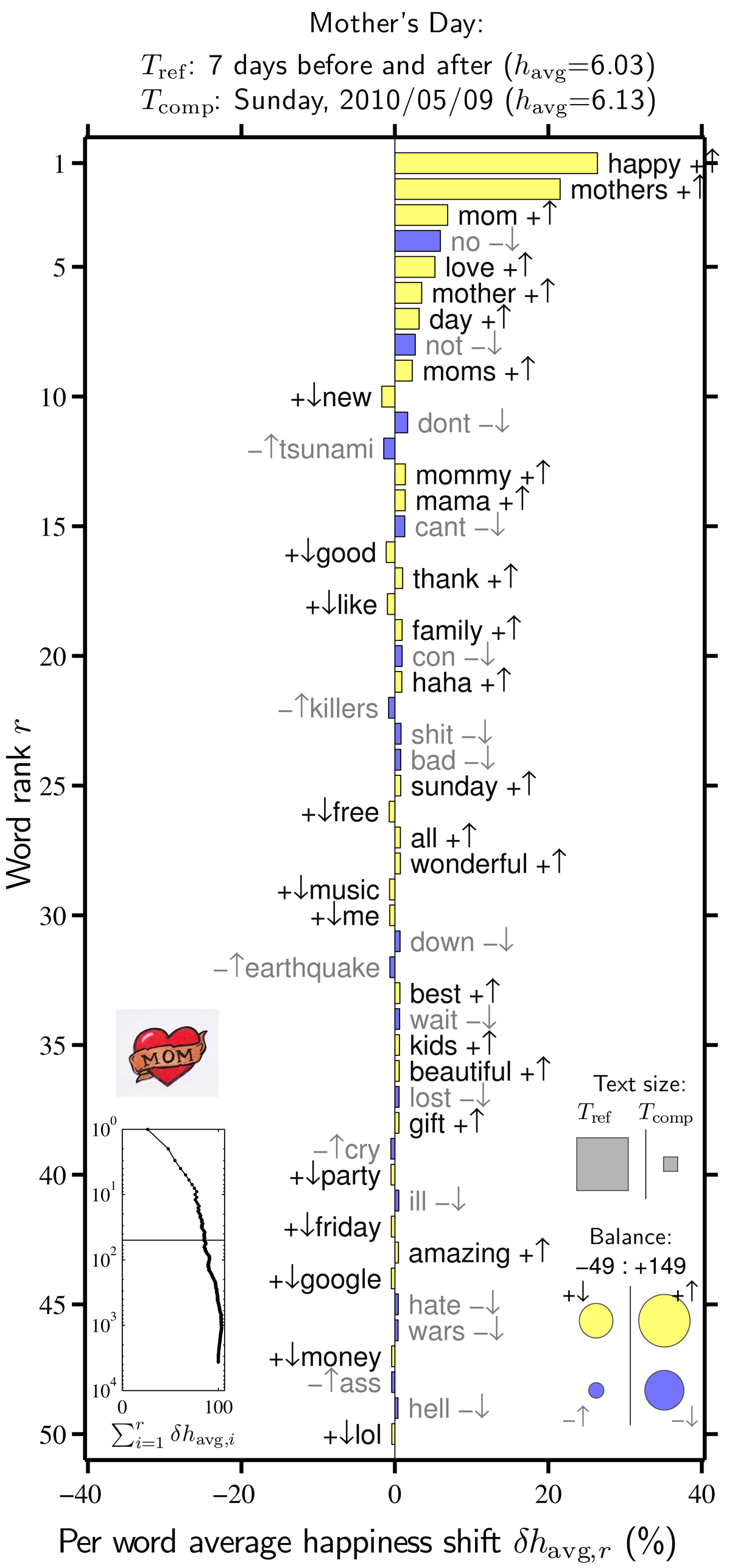

Supplement: Figure S31 — Word shift graph for Mother's Day, 2010/05/09, relative to 7 days before and 7 days after combined. (TIFF) [file pone.0026752.s032.tif]

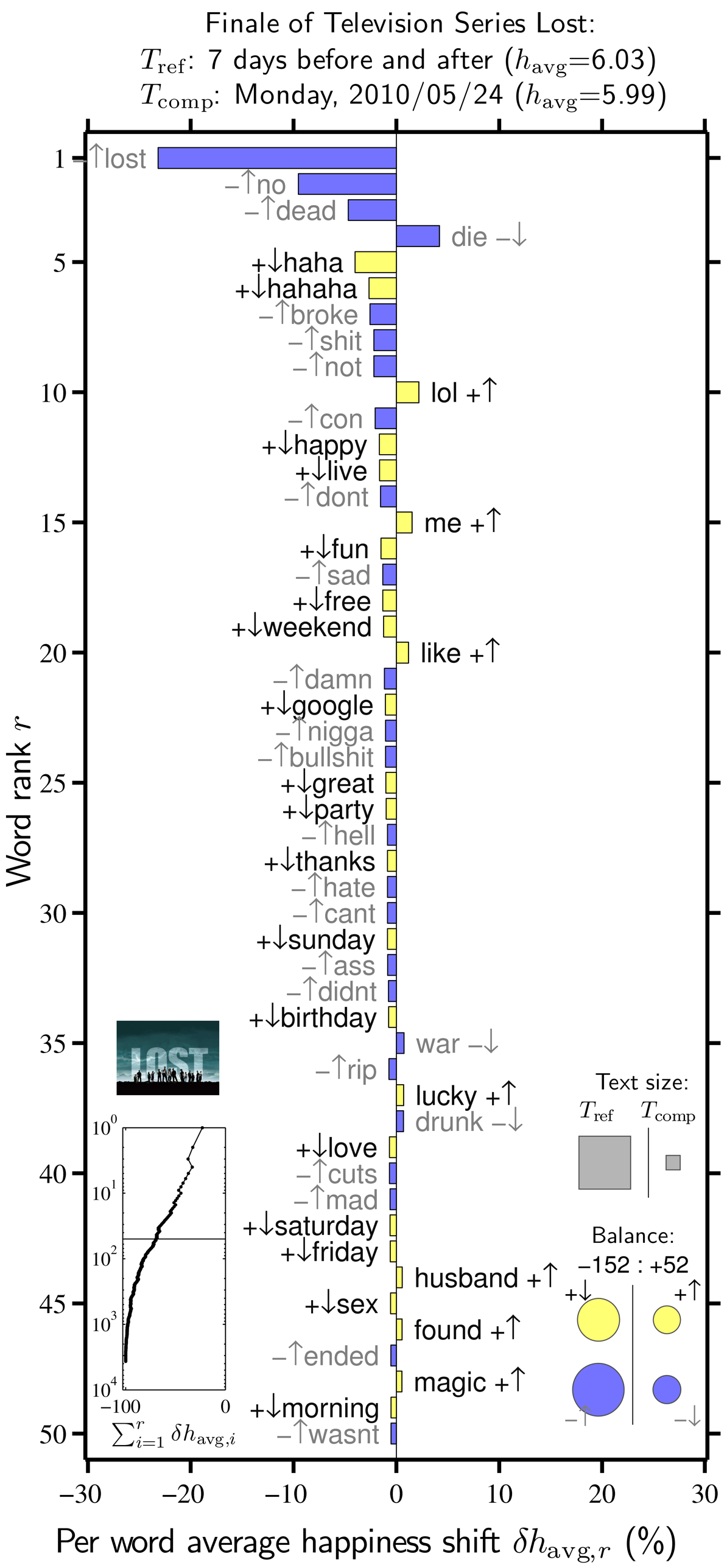

Supplement: Figure S32 — Word shift graph for Finale of Television Series Lost, 2010/05/24, relative to 7 days before and 7 days after combined. (TIFF) [file pone.0026752.s033.tif]

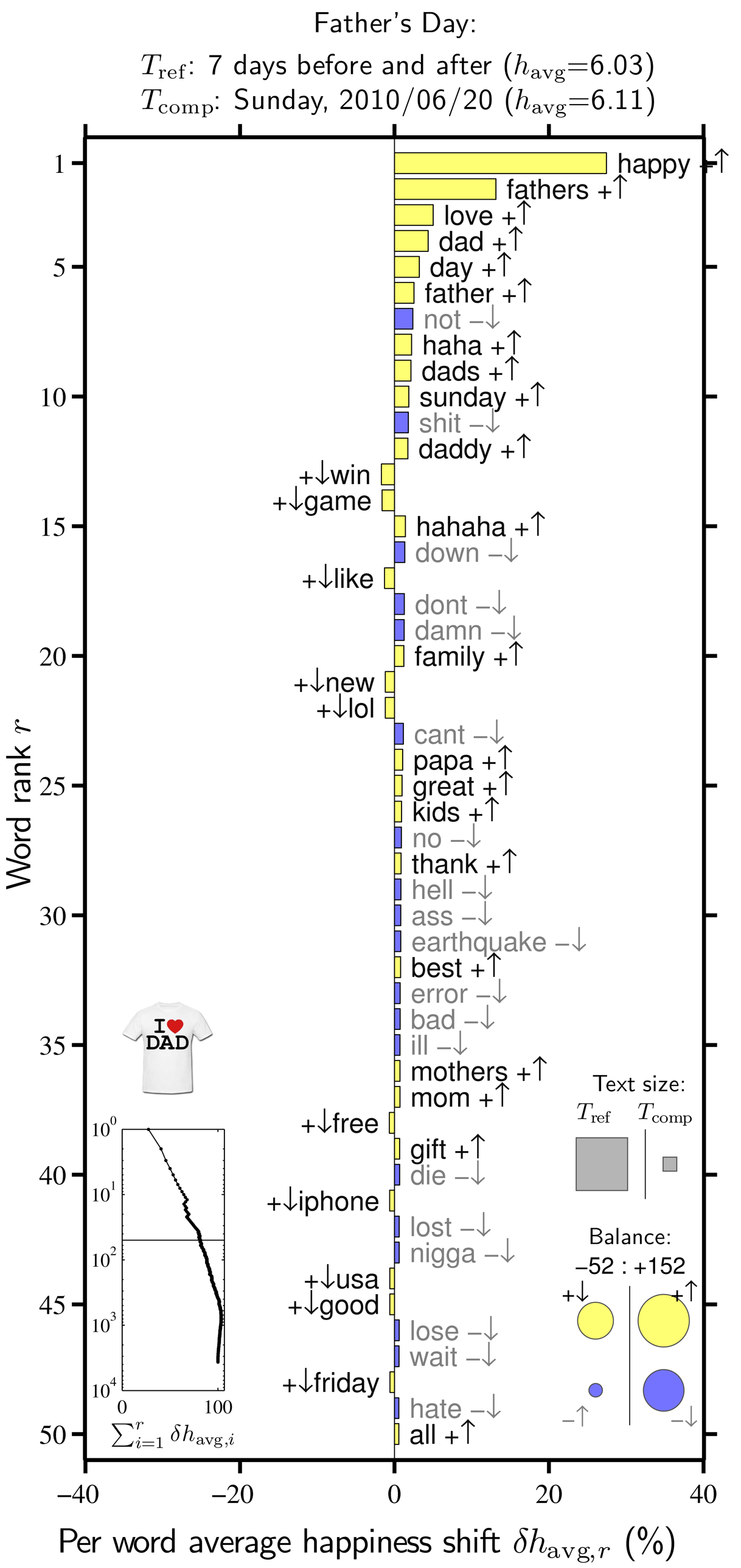

Supplement: Figure S33 — Word shift graph for Father's Day, 2010/06/20, relative to 7 days before and 7 days after combined. (TIFF) [file pone.0026752.s034.tif]

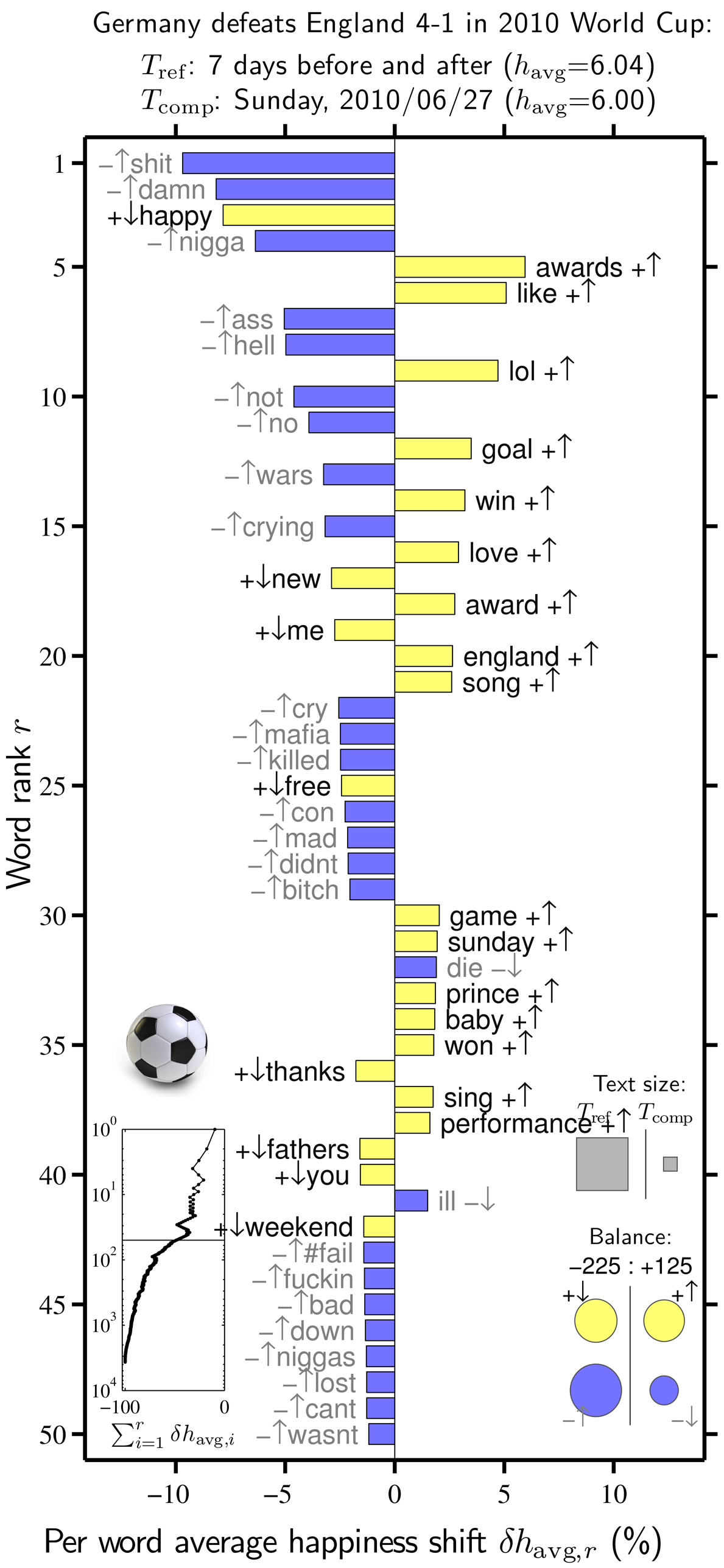

Supplement: Figure S34 — Word shift graph for Germany defeats England 4-1 in 2010 World Cup, 2010/06/27, relative to 7 days before and 7 days after combined. (TIFF) [file pone.0026752.s035.tif]

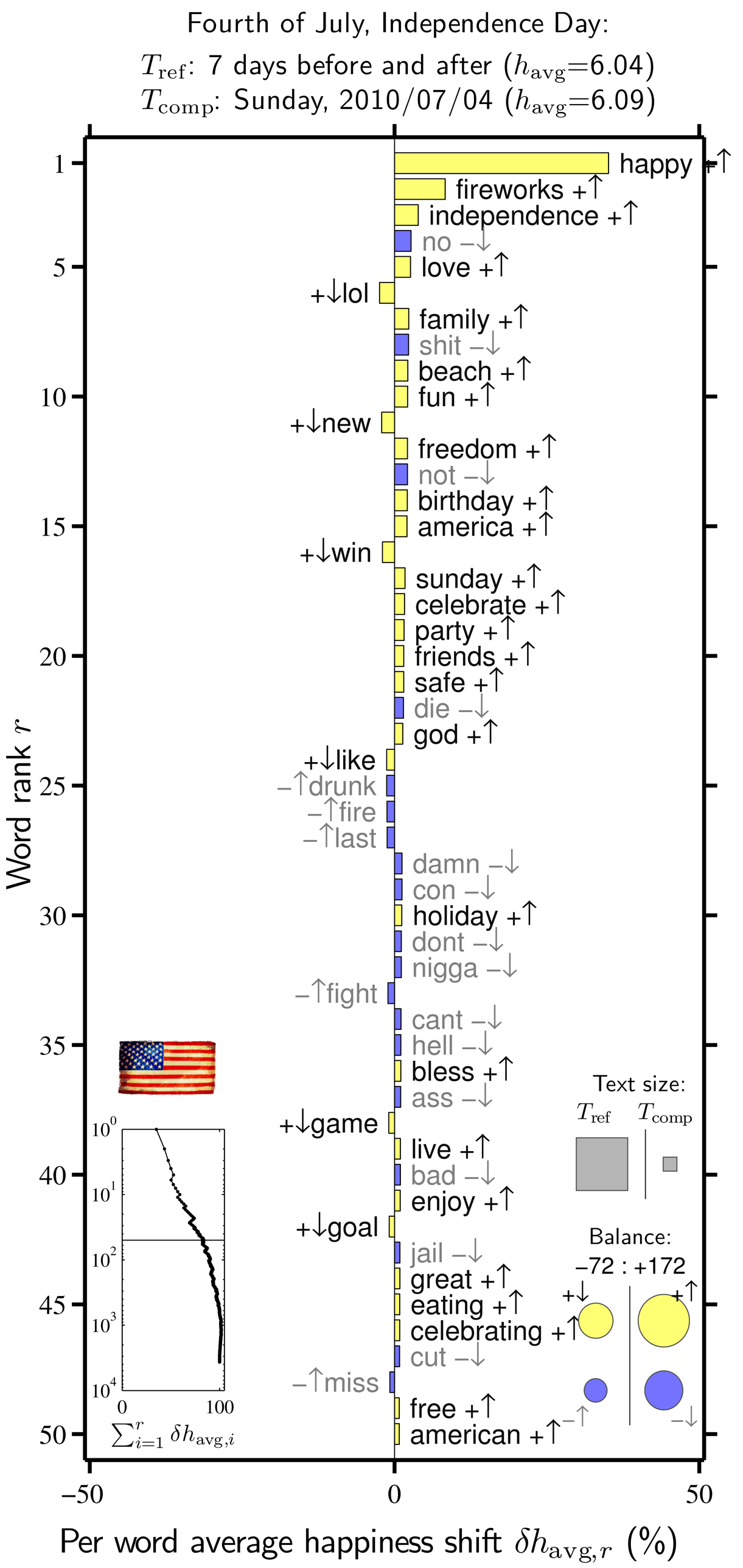

Supplement: Figure S35 — Word shift graph for Fourth of July, Independence Day, 2010/07/04, relative to 7 days before and 7 days after combined. (TIFF) [file pone.0026752.s036.tif]

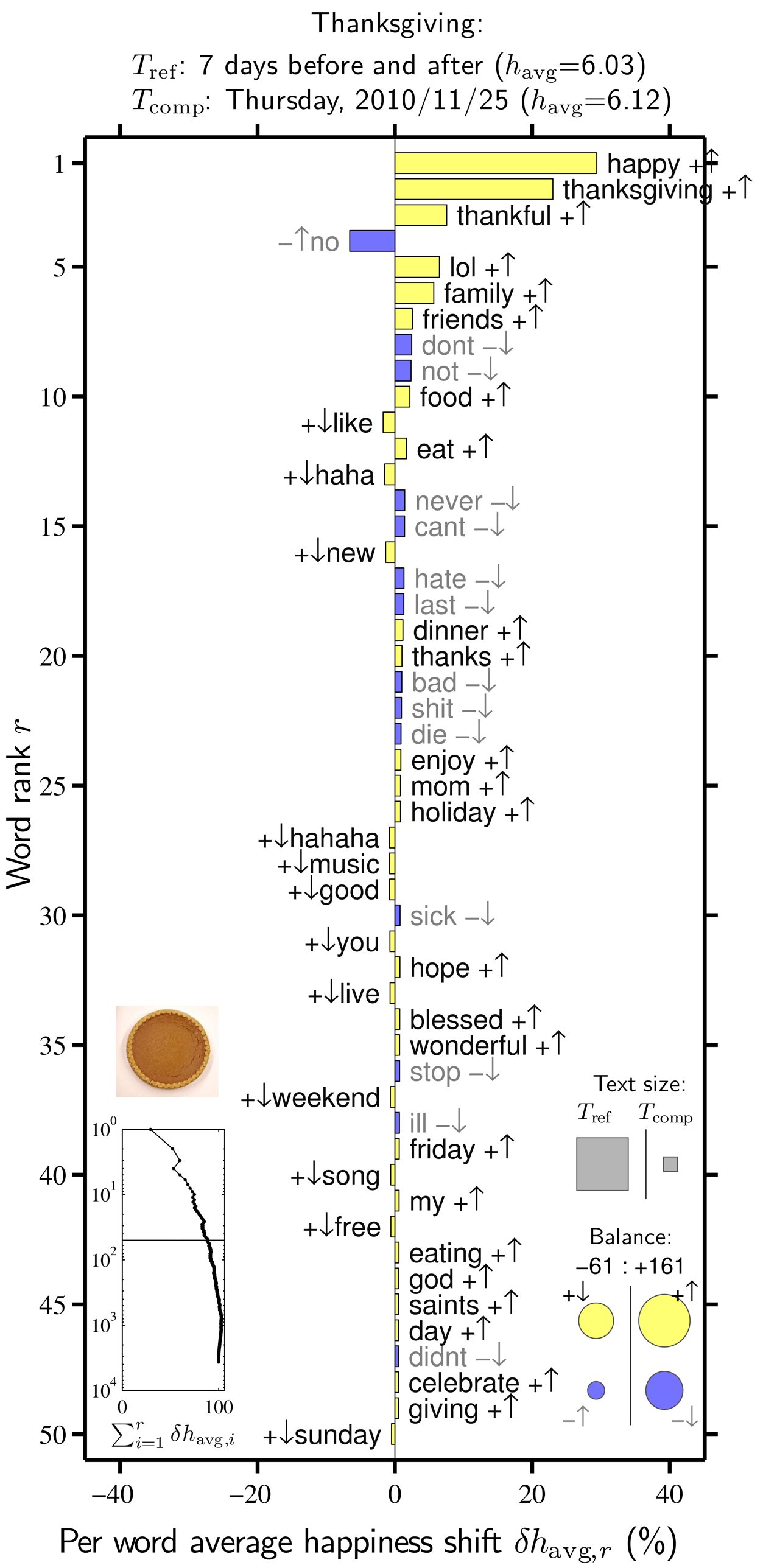

Supplement: Figure S36 — Word shift graph for Thanksgiving, 2010/11/25, relative to 7 days before and 7 days after combined. (TIFF) [file pone.0026752.s037.tif]

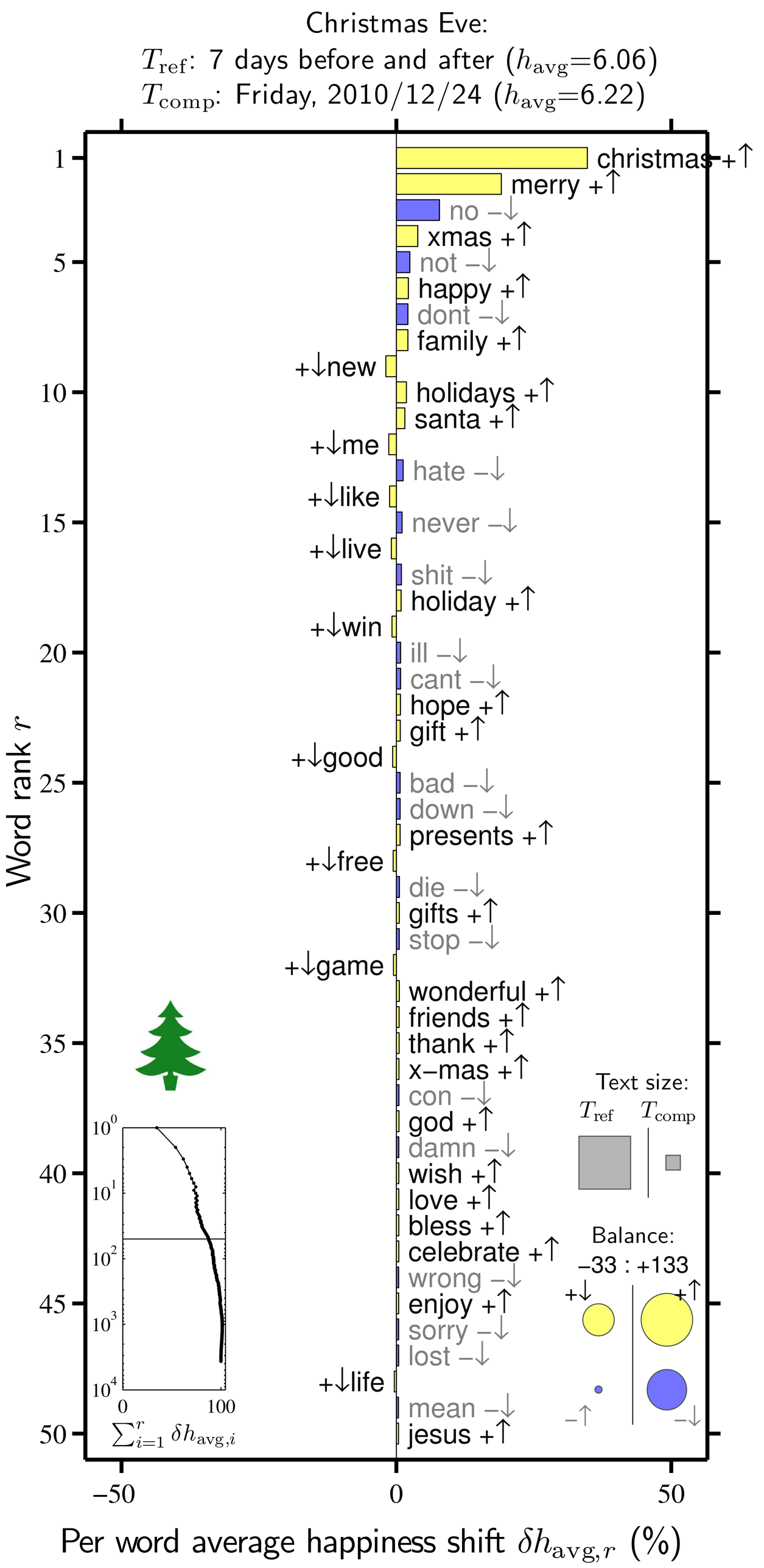

Supplement: Figure S37 — Word shift graph for Christmas Eve, 2010/12/24, relative to 7 days before and 7 days after combined. (TIFF) [file pone.0026752.s038.tif]

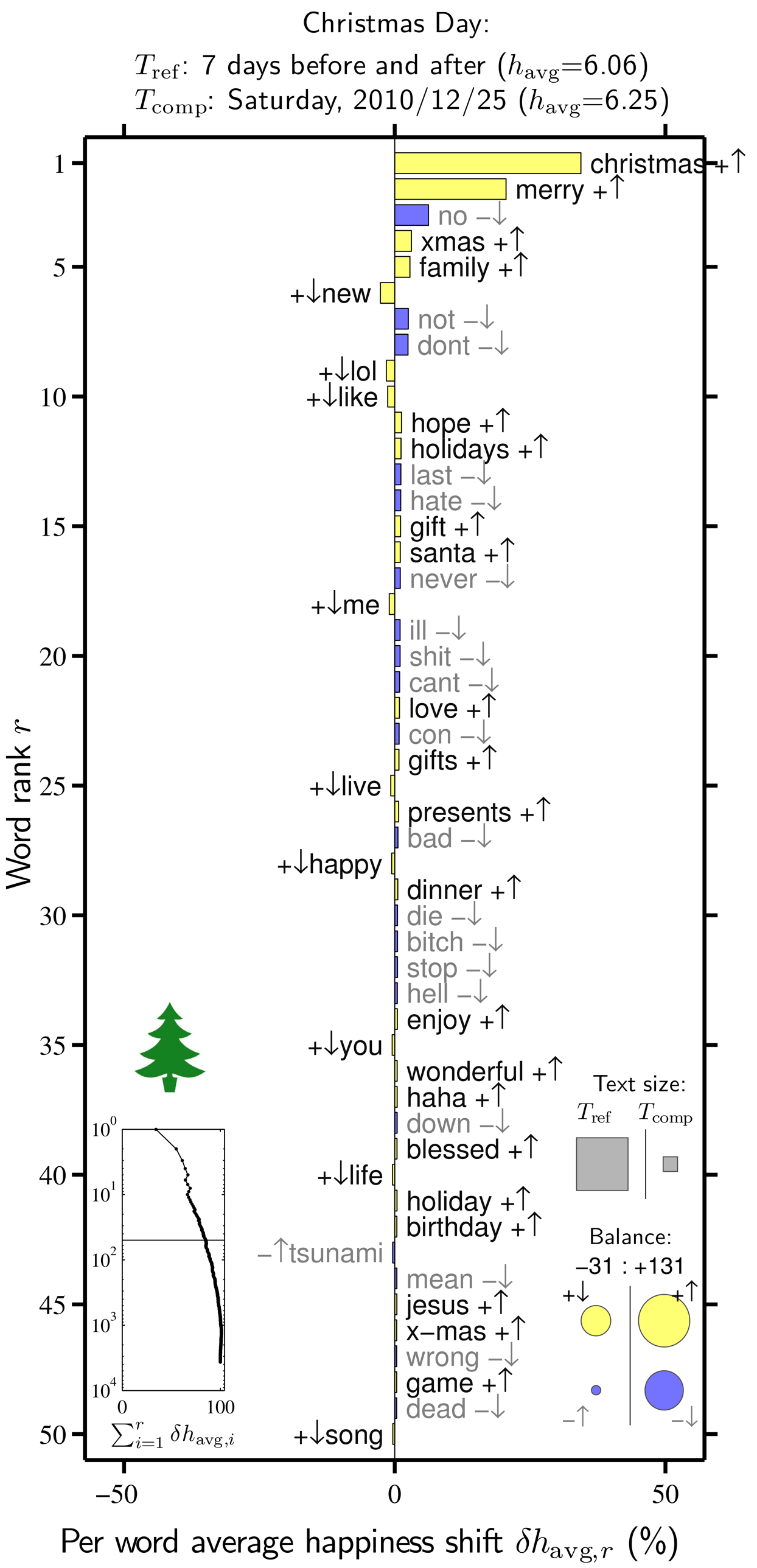

Supplement: Figure S38 — Word shift graph for Christmas Day, 2010/12/25, relative to 7 days before and 7 days after combined. (TIFF) [file pone.0026752.s039.tif]

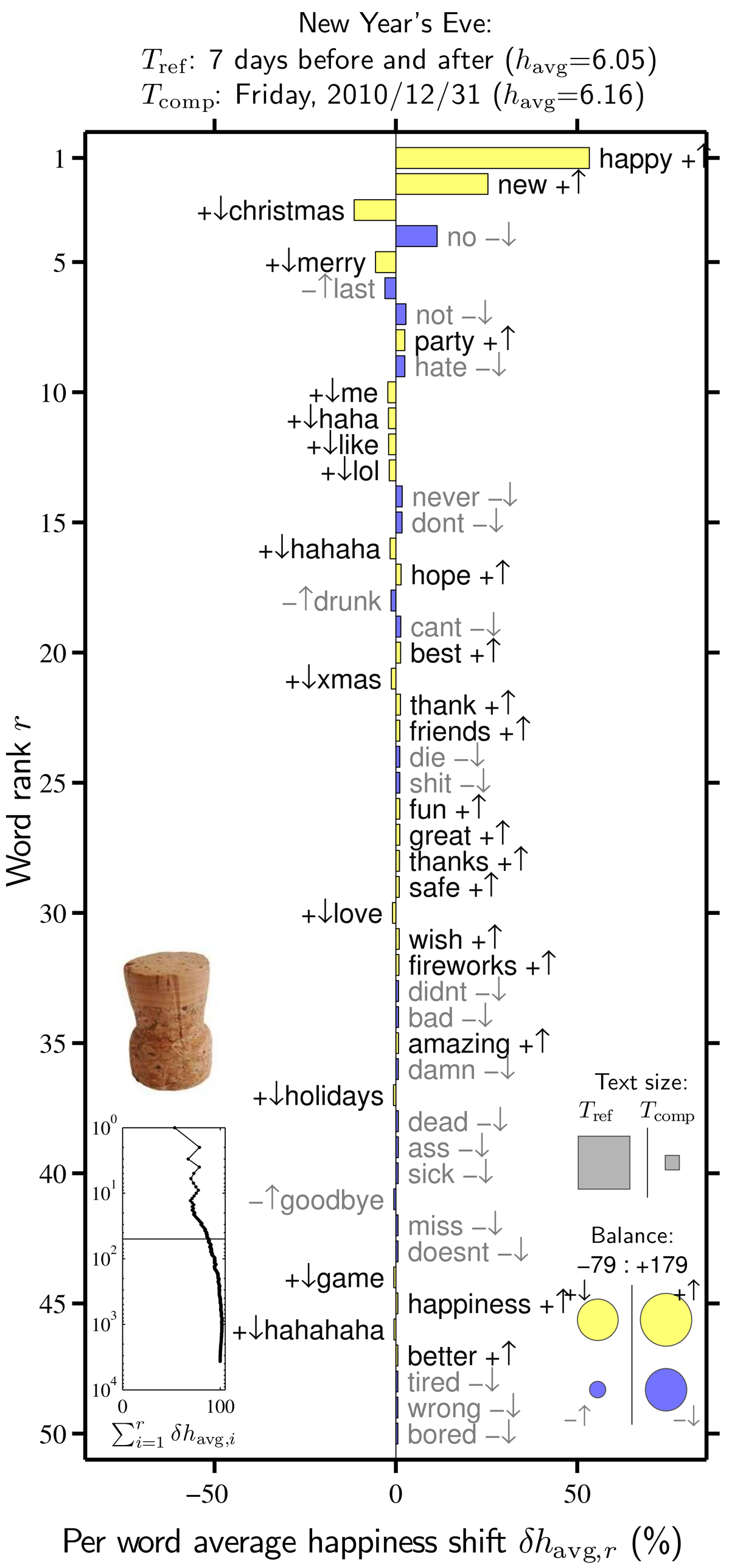

Supplement: Figure S39 — Word shift graph for New Year's Eve, 2010/12/31, relative to 7 days before and 7 days after combined. (TIFF) [file pone.0026752.s040.tif]

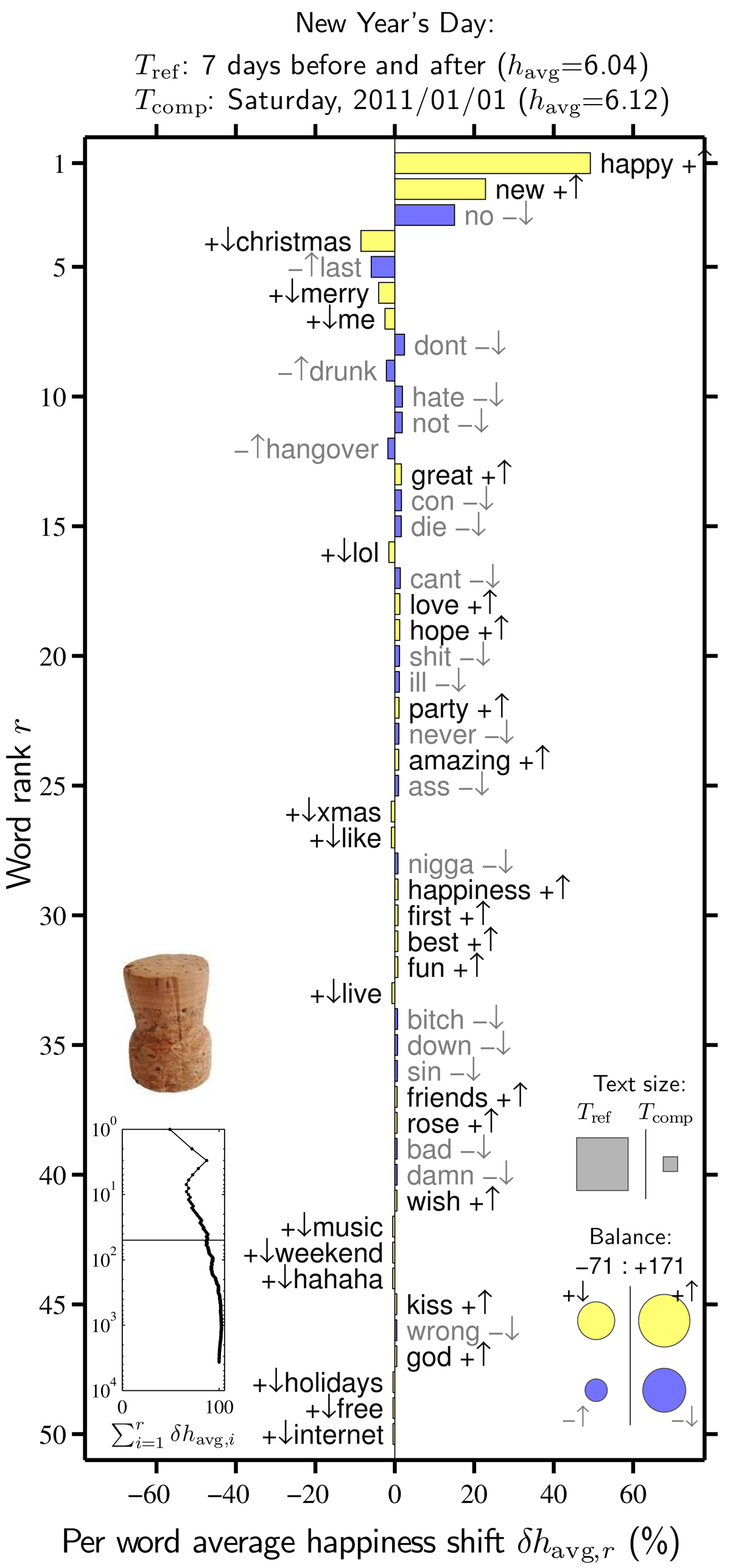

Supplement: Figure S40 — Word shift graph for New Year's Day, 2011/01/01, relative to 7 days before and 7 days after combined. (TIFF) [file pone.0026752.s041.tif]

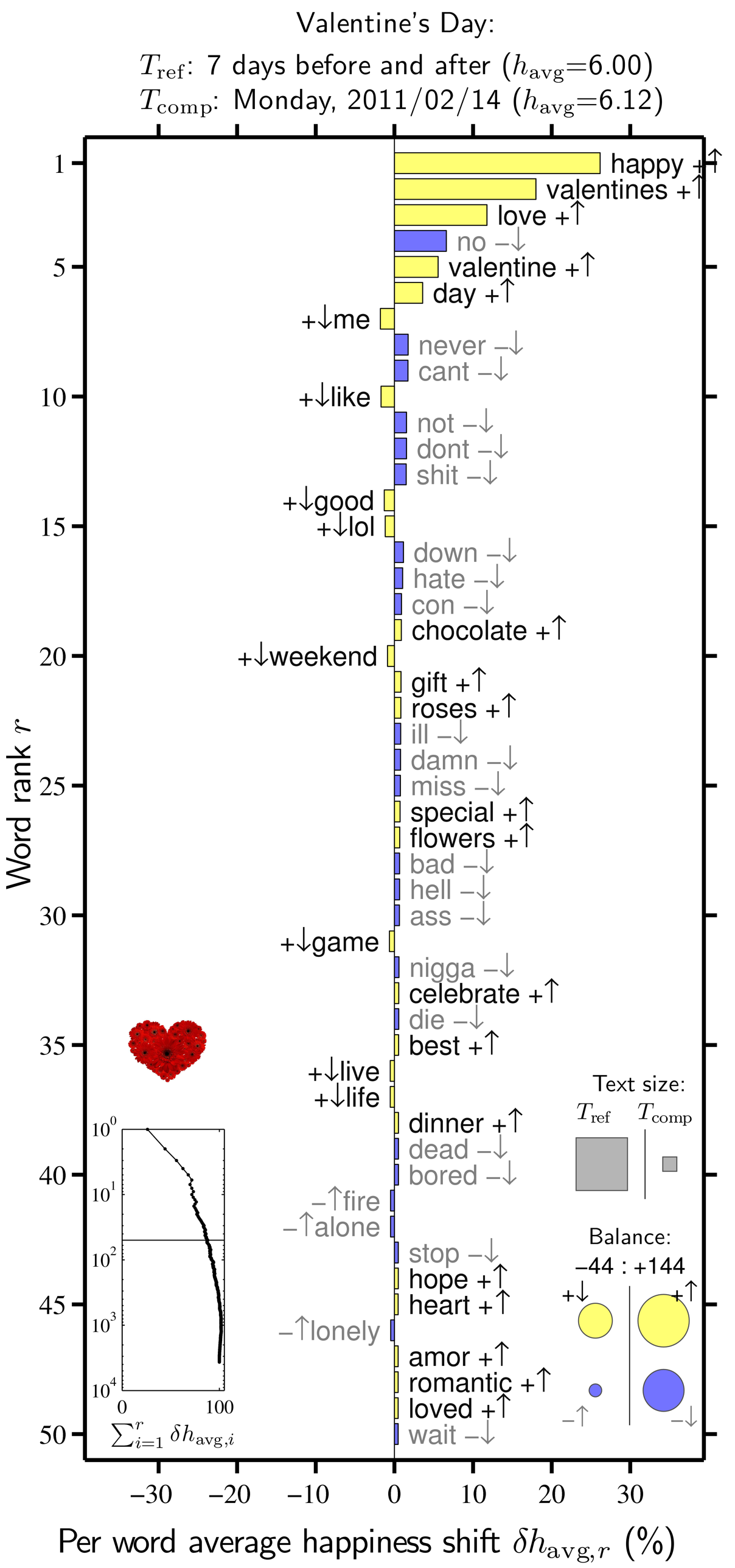

Supplement: Figure S41 — Word shift graph for Valentine's Day, 2011/02/14, relative to 7 days before and 7 days after combined. (TIFF) [file pone.0026752.s042.tif]

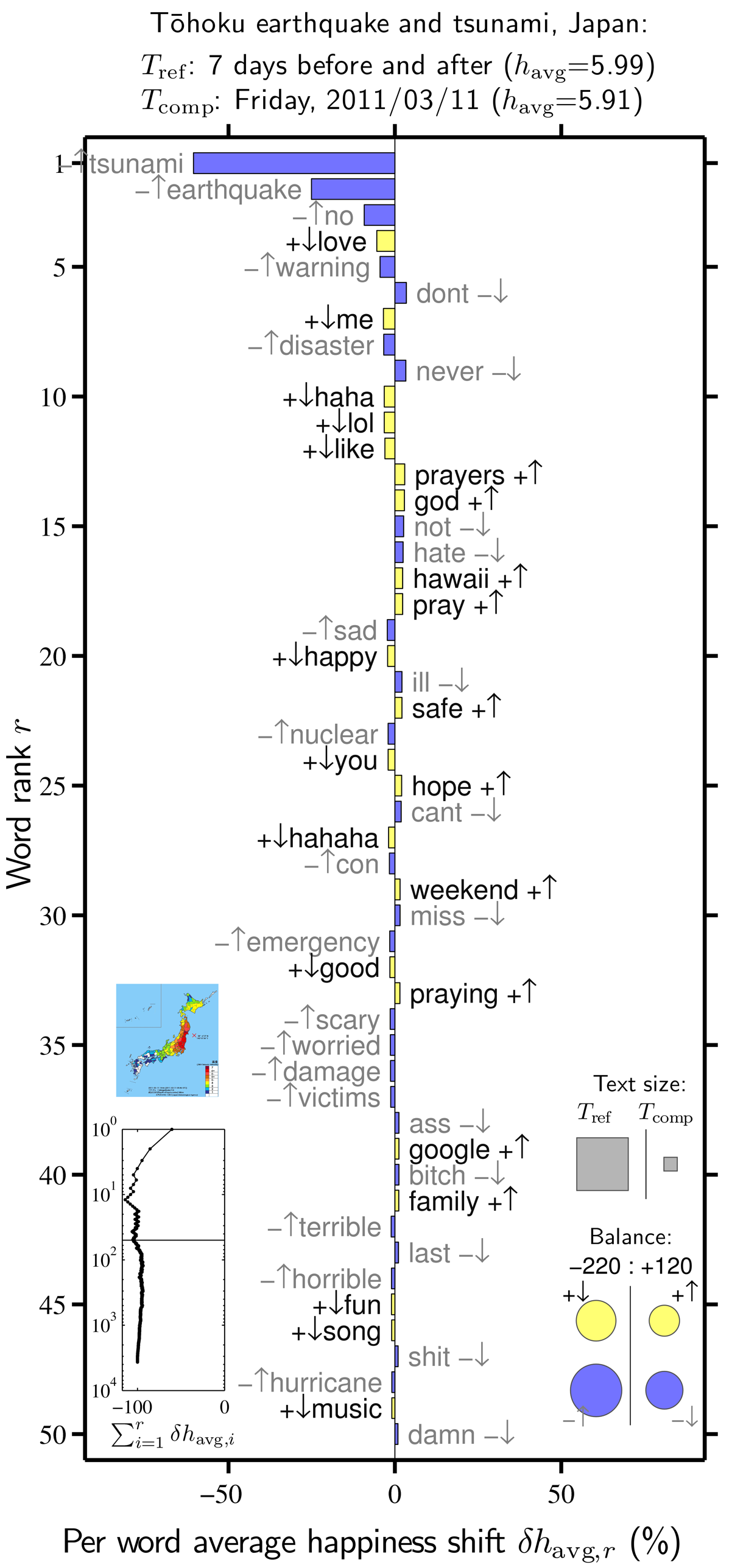

Supplement: Figure S42 — Word shift graph for T hoku earthquake and tsunami, Japan, 2011/03/11, relative to 7 days before and 7 days after combined. (TIFF) [file pone.0026752.s043.tif]

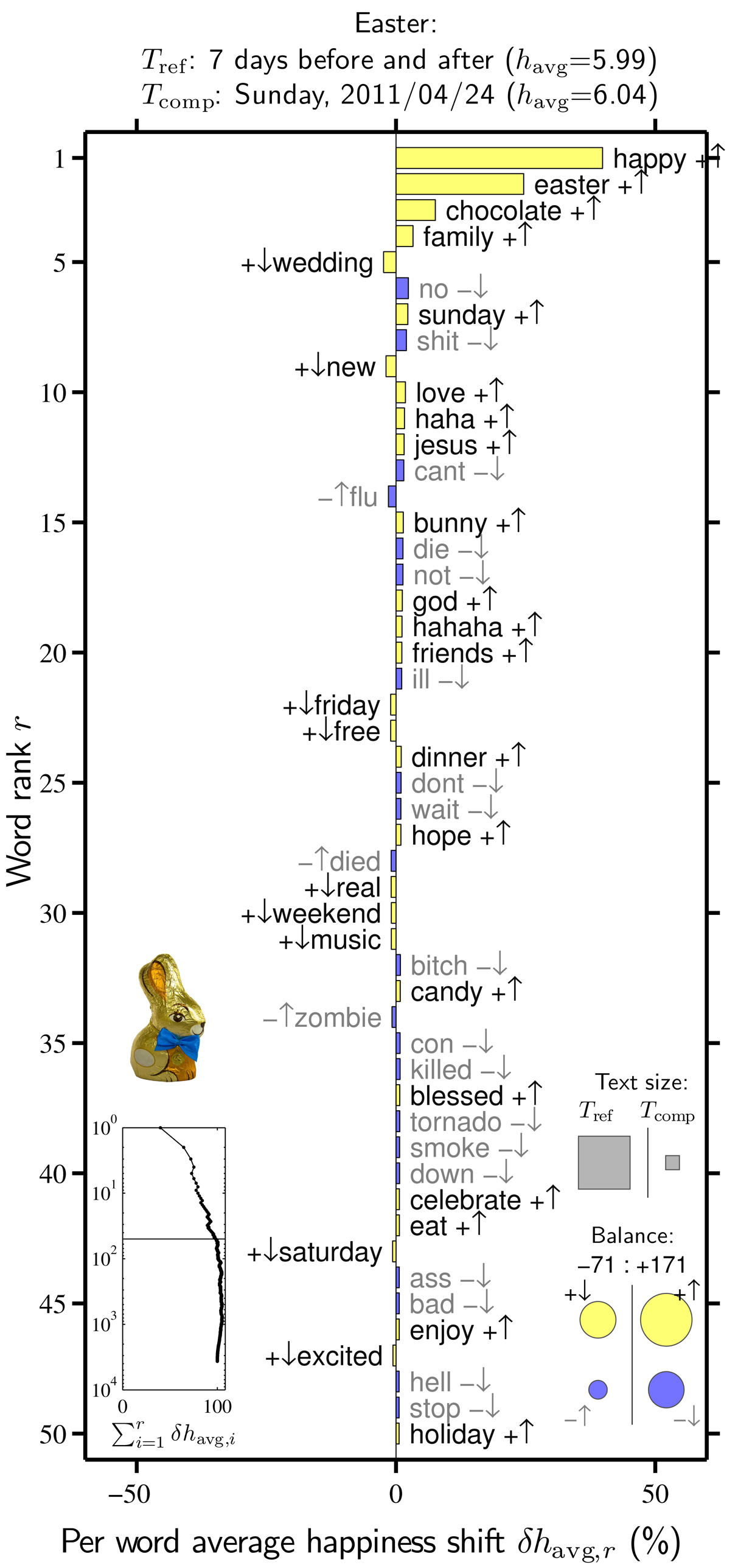

Supplement: Figure S43 — Word shift graph for Easter, 2011/04/24, relative to 7 days before and 7 days after combined. (TIFF) [file pone.0026752.s044.tif]

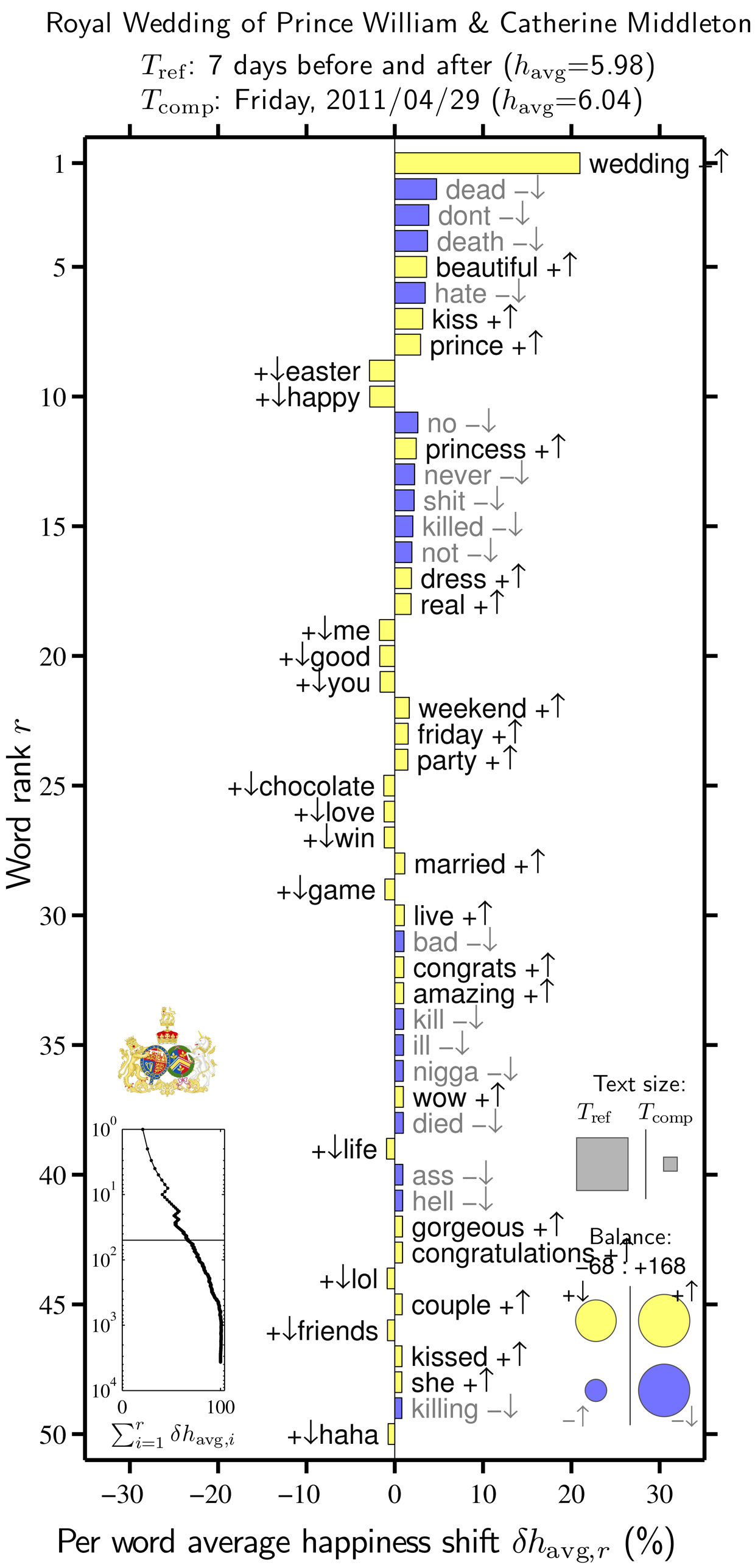

Supplement: Figure S44 — Word shift graph for Royal Wedding of Prince William & Catherine Middleton, 2011/04/29, relative to 7 days before and 7 days after combined. (TIFF) [file pone.0026752.s045.tif]

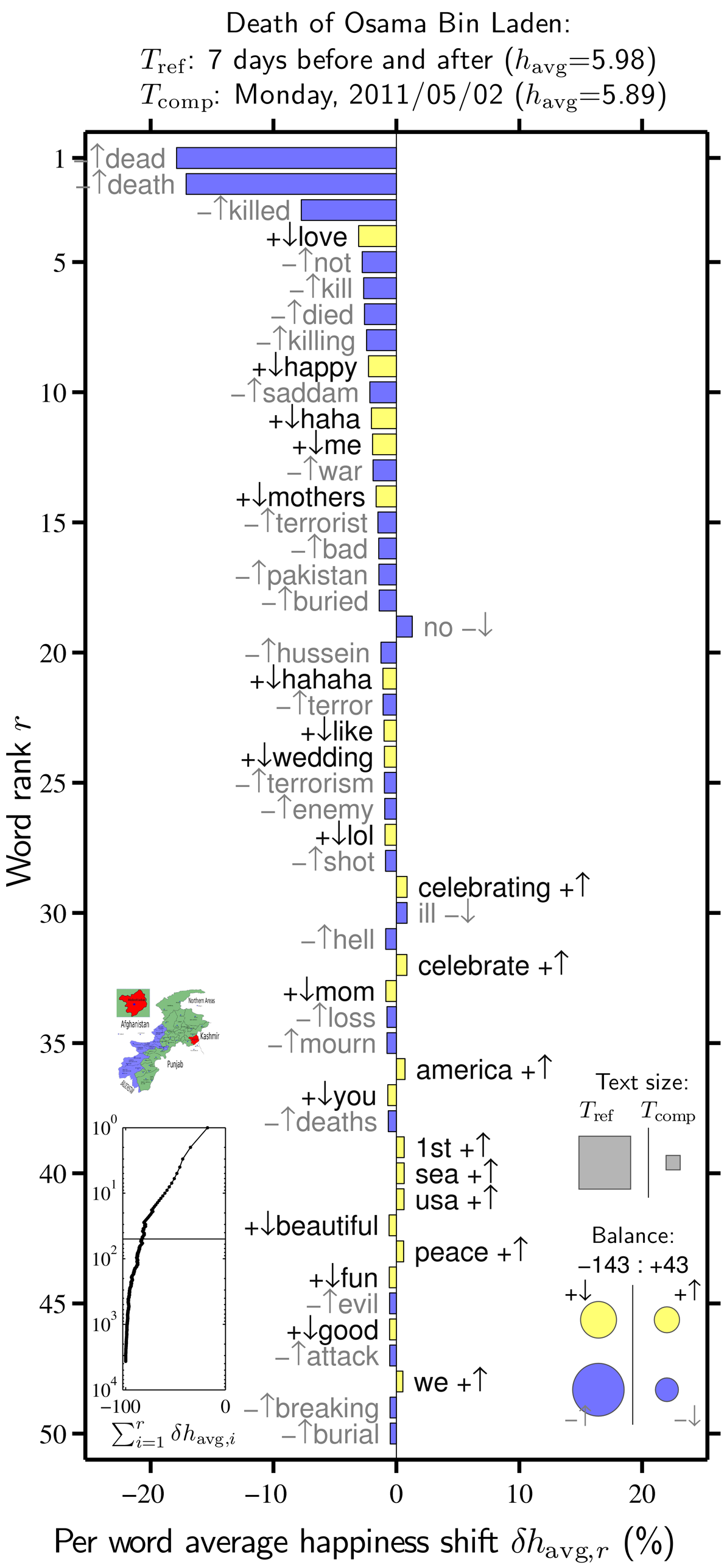

Supplement: Figure S45 — Word shift graph for Death of Osama Bin Laden, 2011/05/02, relative to 7 days before and 7 days after combined. (TIFF) [file pone.0026752.s046.tif]

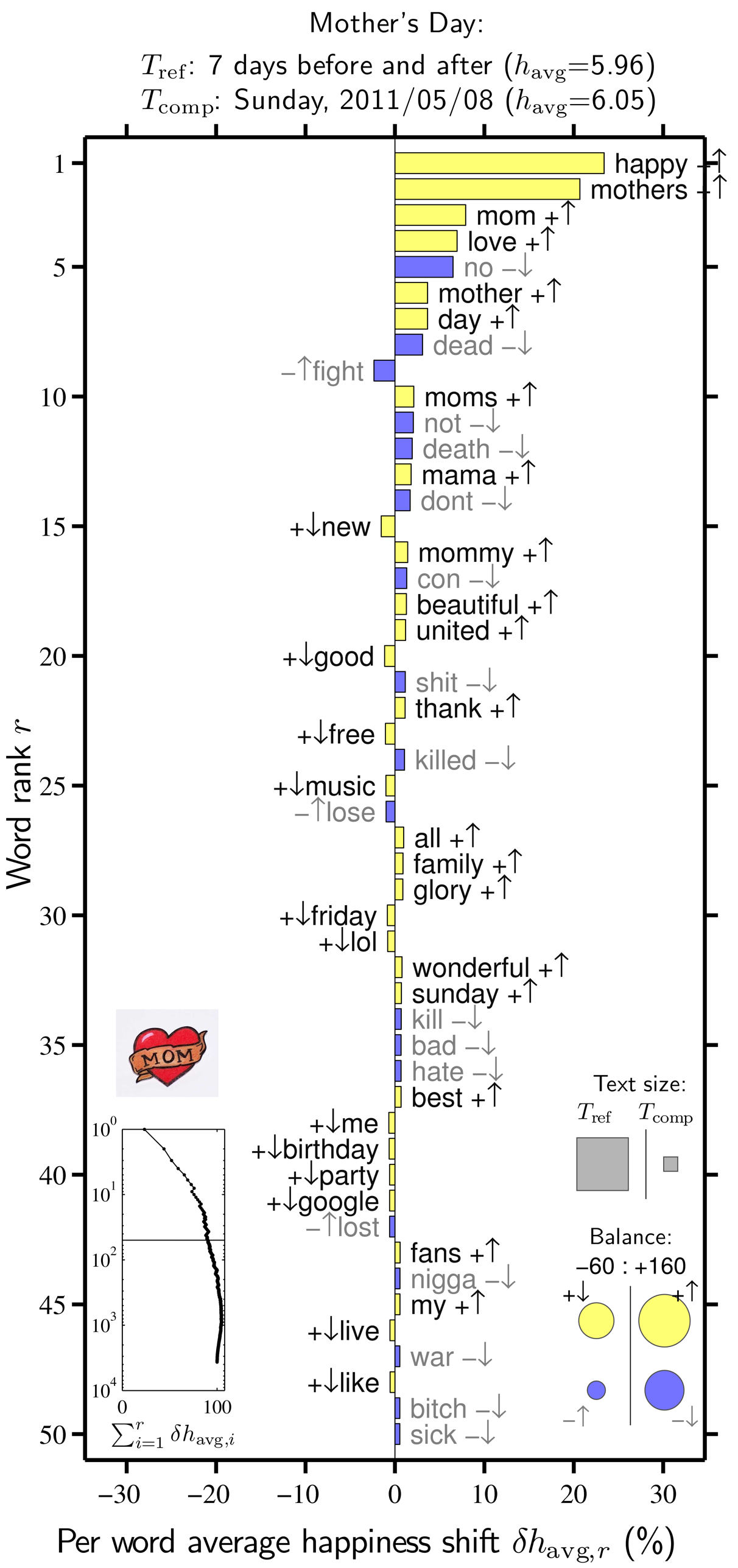

Supplement: Figure S46 — Word shift graph for Mother's Day, 2011/05/08, relative to 7 days before and 7 days after combined. (TIFF) [file pone.0026752.s047.tif]

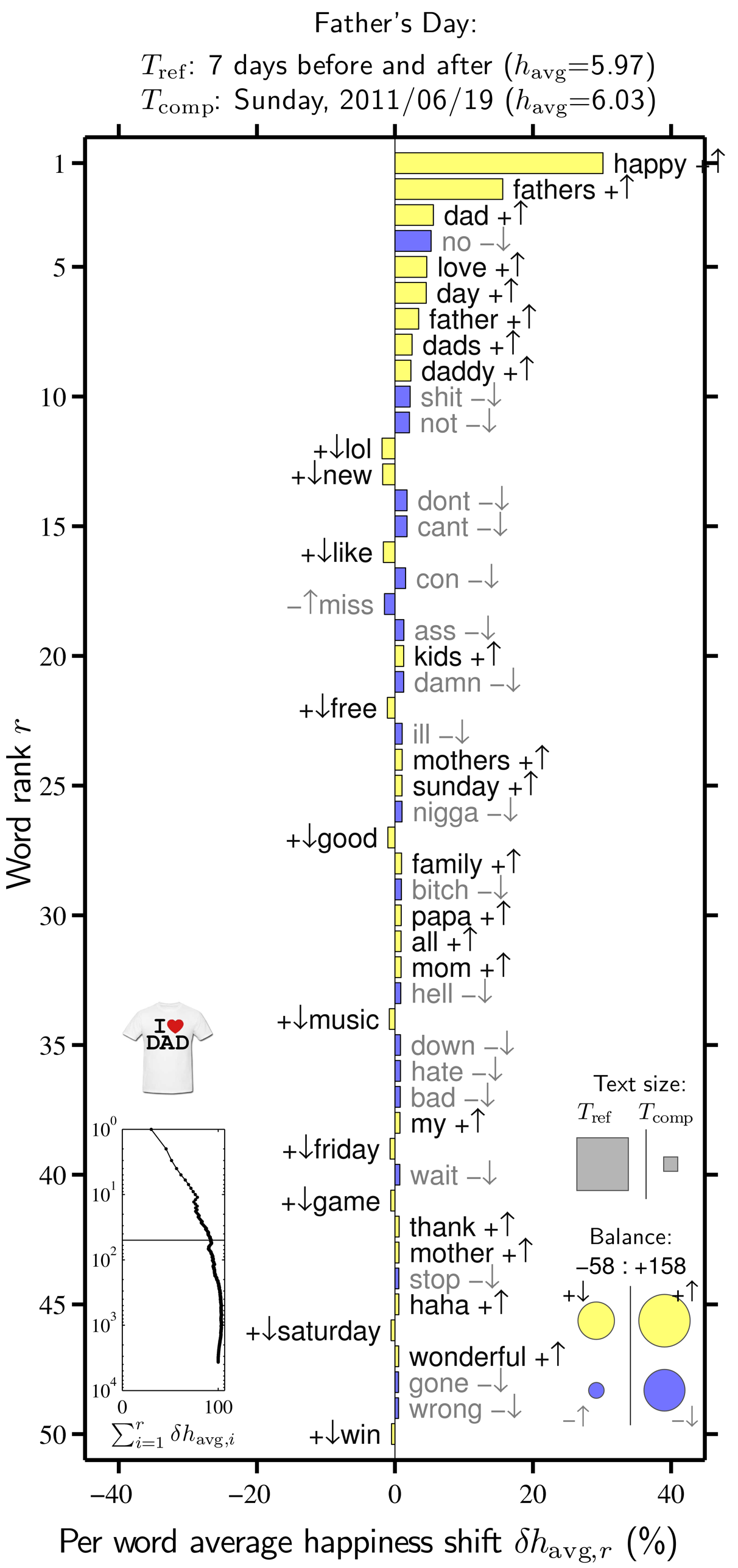

Supplement: Figure S47 — Word shift graph for Father's Day, 2011/06/19, relative to 7 days before and 7 days after combined. (TIFF) [file pone.0026752.s048.tif]

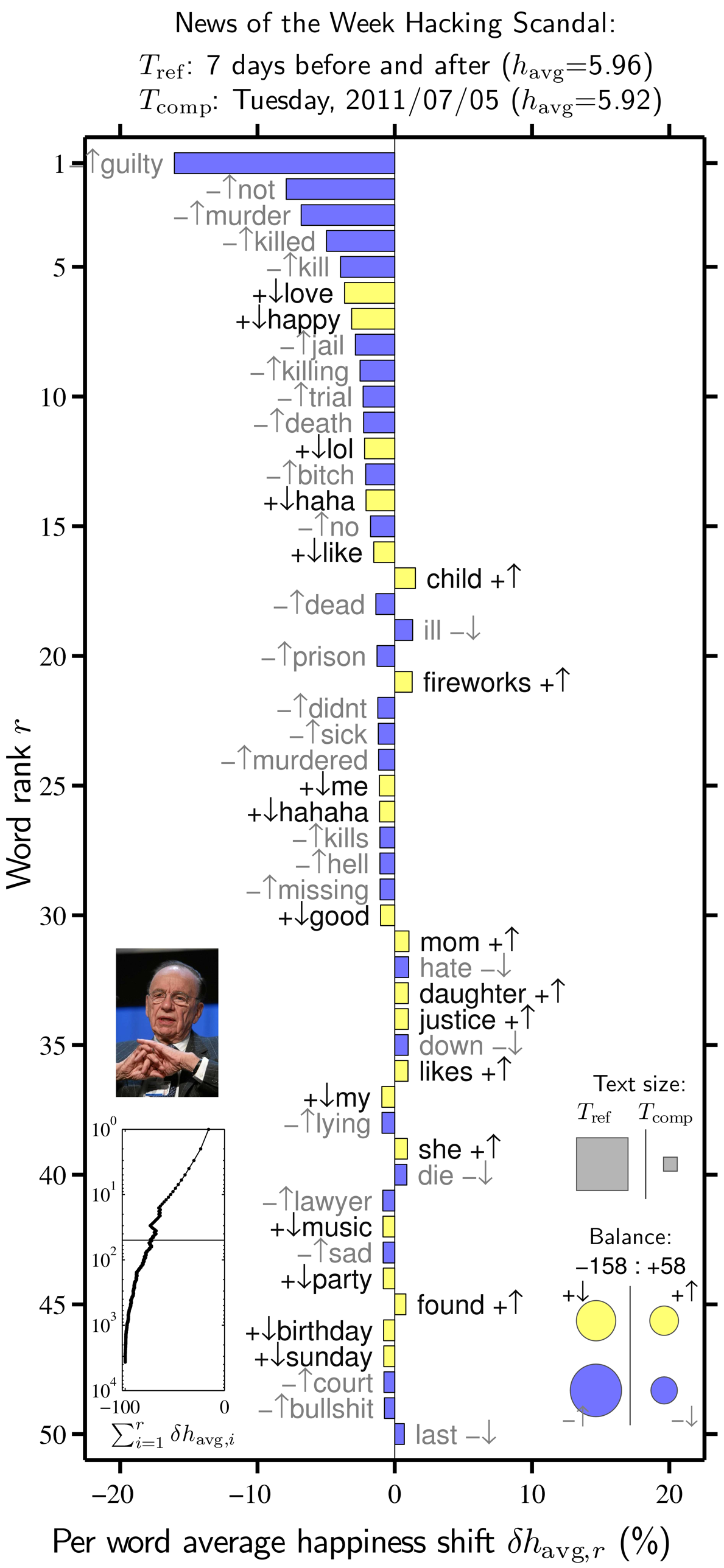

Supplement: Figure S48 — Word shift graph for News of the Week Hacking Scandal, 2011/07/05, relative to 7 days before and 7 days after combined. (TIFF) [file pone.0026752.s049.tif]

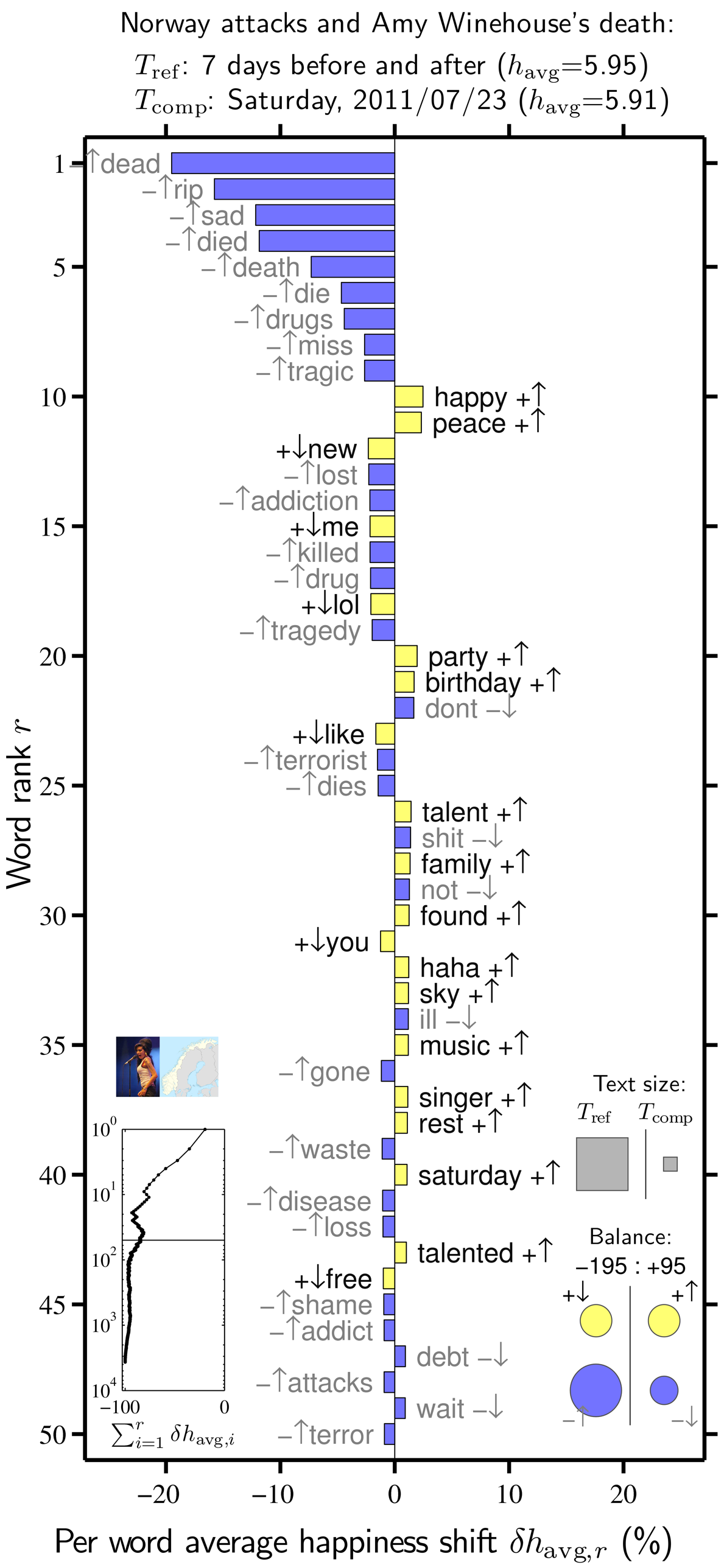

Supplement: Figure S49 — Word shift graph for Norway attacks and Amy Winehouse's death, 2011/07/23, relative to 7 days before and 7 days after combined. (TIFF) [file pone.0026752.s050.tif]

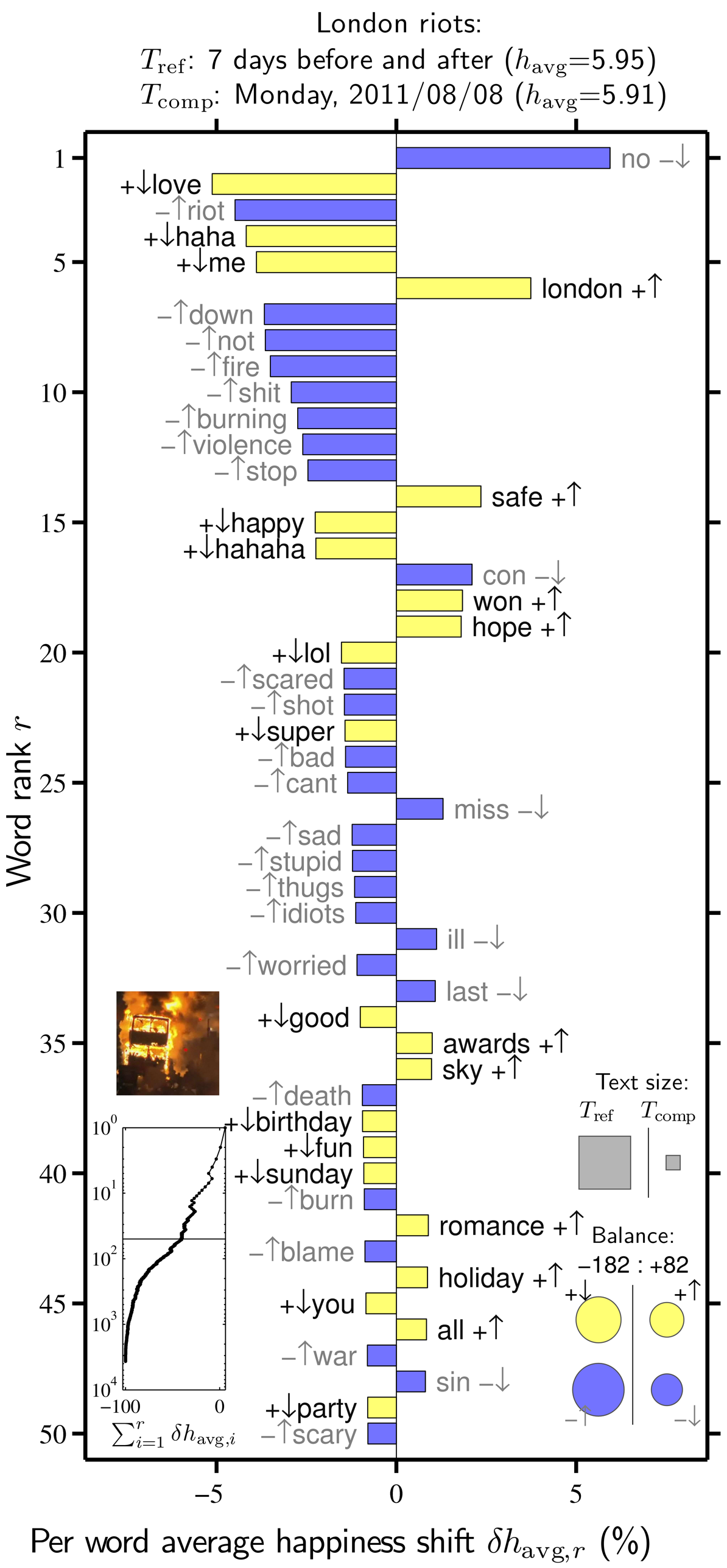

Supplement: Figure S50 — Word shift graph for London riots, 2011/08/08, relative to 7 days before and 7 days after combined. (TIFF) [file pone.0026752.s051.tif]

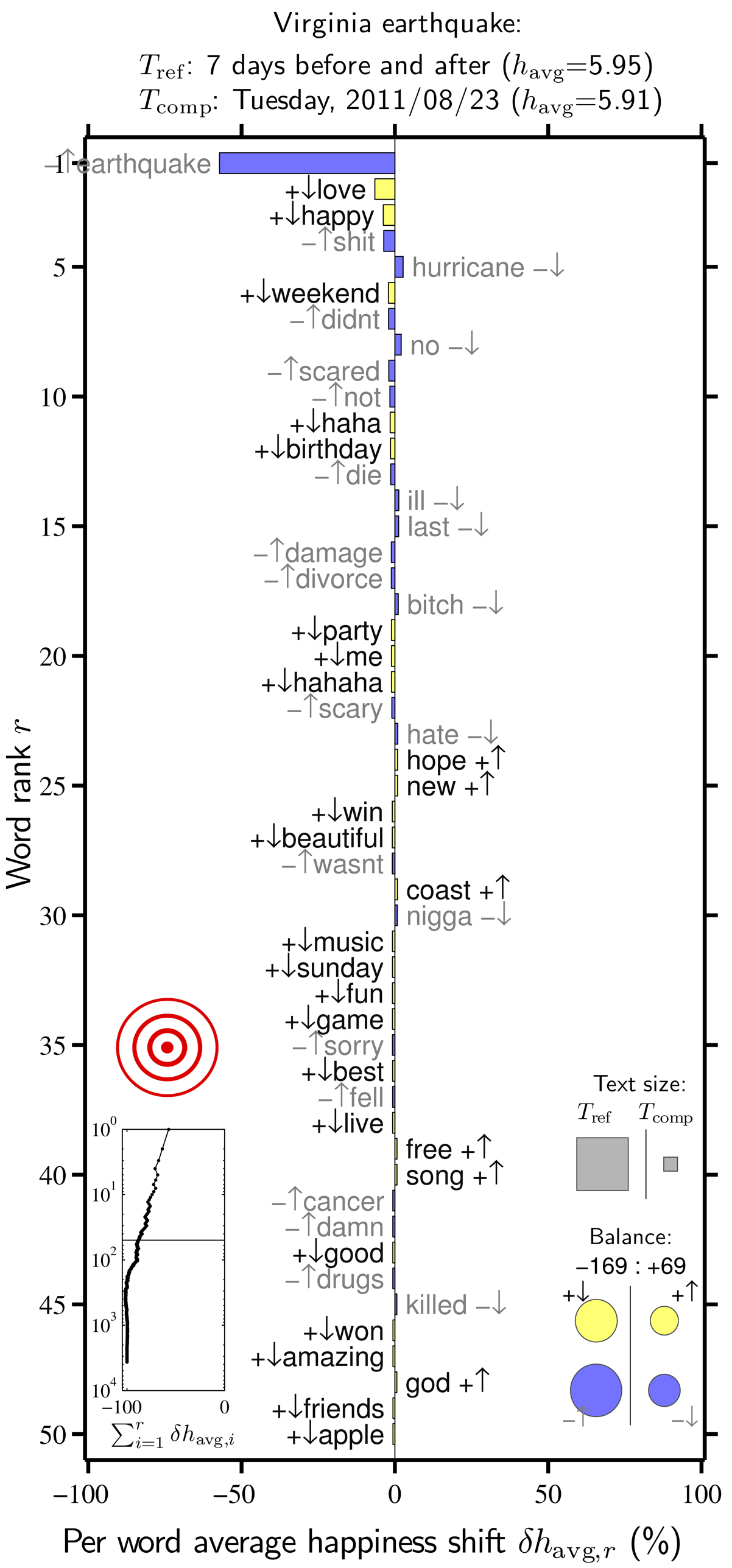

Supplement: Figure S51 — Word shift graph for Virginia earthquake, 2011/08/23, relative to 7 days before and 7 days after combined. (TIFF) [file pone.0026752.s052.tif]

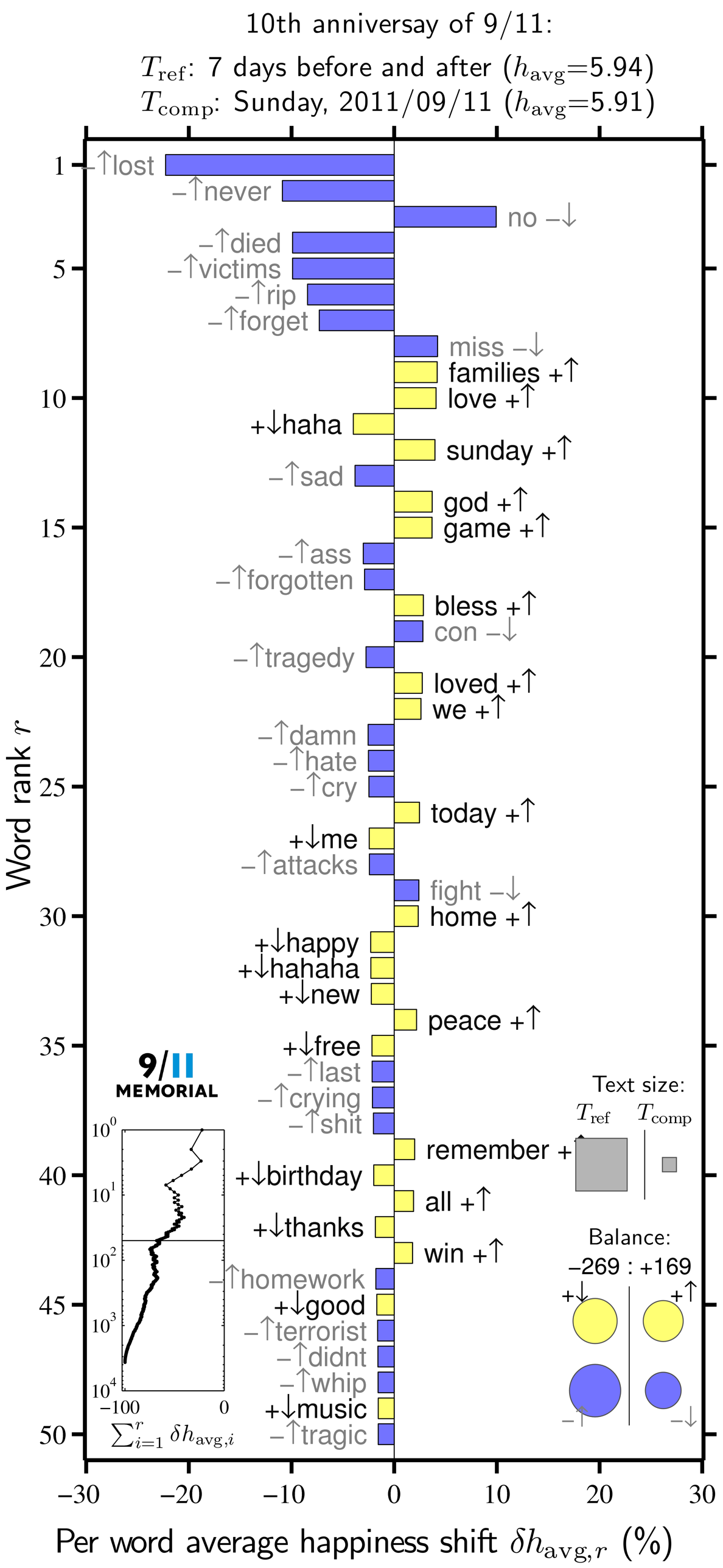

Supplement: Figure S52 — Word shift graph for 10th anniversay of 9/11, 2011/09/11, relative to 7 days before and 7 days after combined. (TIFF) [file pone.0026752.s053.tif]
