# Supplementary material for: Temporal Patterns of Happiness and Information in a Global Social Network: Hedonometrics and Twitter
Source: PLoS One. 2011 Dec 7;6(12):e26752. doi: 10.1371/journal.pone.0026752 (PMC3233600; doi:10.1371/journal.pone.0026752)
Supplement: Table S1 — The same selection of 100 keywords and text elements listed in the main text's Table 2 , reordered by normalized happiness . (PDF) [file pone.0026752.s054.pdf]

Word	$h_{\rm avg}^{\rm (norm)}$	Total Tweets	$h_{\rm avg}^{\rm (amb)}$	Word	$h_{\rm avg}^{\rm (norm)}$	Total Tweets	$h_{\rm avg}^{\rm (amb)}$
1. happy	+1.104	1.65e+07 (13)	+0.430 (1)	51. climate	-0.160	3.64e+05 (80)	-0.135 (64)
2. love	+0.977	4.67e+07 (6)	+0.164 (11)	52. man	-0.163	1.59e+07 (14)	-0.175 (71)
3. Christmas	+0.953	4.89e+06 (35)	+0.404 (2)	53. yesterday	-0.168	3.08e+06 (42)	+0.033 (37)
4. win	+0.924	7.98e+06 (26)	+0.204 (8)	54. hot	-0.172	7.12e+06 (28)	+0.095 (26)
5. vacation	+0.817	9.35e+05 (67)	+0.200 (9)	55. Obama	-0.173	2.98e+06 (44)	-0.205 (76)
6. sun	+0.737	2.39e+06 (52)	+0.144 (16)	56. work	-0.174	1.84e+07 (11)	-0.010 (46)
7. family	+0.716	5.01e+06 (32)	+0.251 (5)	57. commute	-0.206	9.01e+04 (94)	-0.048 (50)
8. friends	+0.685	7.67e+06 (27)	+0.155 (12)	58. they	-0.208	2.74e+07 (8)	-0.159 (67)
9. party	+0.679	6.44e+06 (29)	+0.170 (10)	59. Michael Jackson	-0.213	8.26e+05 (70)	+0.018 (41)
10. heaven	+0.674	7.42e+05 (71)	+0.041 (34)	60. them	-0.280	1.54e+07 (15)	-0.090 (55)
11. kiss	+0.632	1.70e+06 (59)	+0.072 (30)	61. election	-0.306	5.60e+05 (75)	-0.127 (60)
12. :)	+0.630	1.04e+07 (20)	+0.274 (4)	62. Pope	-0.316	1.52e+05 (91)	-0.277 (83)
13. income	+0.621	5.10e+05 (76)	+0.137 (17)	63. left	-0.383	4.89e+06 (34)	-0.118 (58)
14. cash	+0.601	1.28e+06 (63)	+0.146 (15)	64. Democrat	-0.384	9.32e+04 (93)	-0.226 (77)
15. Valentine	+0.593	2.47e+05 (84)	+0.127 (20)	65. oil	-0.411	1.38e+06 (62)	-0.162 (68)
16. :-)	+0.560	1.67e+06 (60)	+0.228 (6)	66. RT	-0.443	3.39e+08 (1)	+0.028 (40)
17. sex	+0.542	3.55e+06 (39)	-0.008 (45)	67. gas	-0.471	1.02e+06 (65)	-0.193 (74)
18. coffee	+0.518	2.80e+06 (46)	+0.147 (14)	68. ?	-0.503	2.32e+06 (53)	+0.030 (39)
19. hope	+0.515	1.18e+07 (18)	+0.149 (13)	69. economy	-0.525	6.09e+05 (73)	-0.203 (75)
20. God	+0.468	8.58e+06 (25)	+0.099 (25)	70. Republican	-0.539	2.30e+05 (86)	-0.181 (72)
21. health	+0.447	2.58e+06 (50)	-0.000 (44)	71. cold	-0.546	3.67e+06 (36)	-0.162 (69)
22. life	+0.422	1.40e+07 (17)	+0.012 (43)	72. gay	-0.552	2.73e+06 (47)	-0.152 (65)
23. ;-)	+0.395	9.39e+05 (66)	+0.041 (35)	73. Muslim	-0.569	2.15e+05 (88)	-0.262 (81)
24. girl	+0.331	1.01e+07 (22)	-0.010 (47)	74. Congress	-0.580	3.92e+05 (79)	-0.231 (78)
25. ;)	+0.326	2.61e+06 (48)	+0.094 (27)	75. Senate	-0.601	4.48e+05 (78)	-0.340 (90)
26. USA	+0.325	2.16e+06 (54)	+0.113 (22)	76. Sarah Palin	-0.681	2.26e+05 (87)	-0.128 (61)
27. yes	+0.321	1.16e+07 (19)	+0.056 (31)	77. mosque	-0.694	6.98e+04 (95)	-0.709 (98)
28. Jesus	+0.247	2.03e+06 (56)	+0.094 (28)	78. Islam	-0.710	1.87e+05 (89)	-0.299 (86)
29. summer	+0.221	3.00e+06 (43)	+0.135 (18)	79. Lehman Brothers	-0.721	8.50e+03 (100)	-0.078 (54)
30. woman	+0.202	2.54e+06 (51)	-0.115 (57)	80. George Bush	-0.747	3.23e+04 (98)	-0.333 (87)
31. !	+0.195	3.44e+06 (40)	+0.106 (23)	81. dark	-0.766	1.58e+06 (61)	+0.031 (38)
32. me	+0.160	1.44e+08 (4)	-0.119 (59)	82. Glenn Beck	-0.776	1.14e+05 (92)	-0.282 (85)
33. our	+0.159	1.41e+07 (16)	+0.207 (7)	83. BP	-0.902	5.82e+05 (74)	-0.355 (91)
34. we	+0.146	3.91e+07 (7)	+0.035 (36)	84. Goldman Sachs	-0.984	5.27e+04 (96)	-0.337 (88)
35. right	+0.126	1.92e+07 (10)	-0.090 (56)	85. :-(	-1.174	3.40e+05 (81)	-0.455 (95)
36. today	+0.126	2.56e+07 (9)	+0.092 (29)	86. lose	-1.181	2.06e+06 (55)	-0.157 (66)
37. you	+0.111	1.73e+08 (3)	+0.052 (33)	87. Iraq	-1.282	2.39e+05 (85)	-0.773 (100)
38. tomorrow	+0.086	1.04e+07 (21)	+0.054 (32)	88. :(	-1.288	2.89e+06 (45)	-0.472 (96)
39. snow	+0.083	2.60e+06 (49)	-0.051 (51)	89. sad	-1.366	3.56e+06 (38)	-0.187 (73)
40. night	+0.074	1.71e+07 (12)	+0.014 (42)	90. no	-1.415	9.51e+07 (5)	-0.132 (62)
41. boy	+0.062	4.93e+06 (33)	-0.026 (48)	91. drugs	-1.452	5.10e+05 (77)	-0.382 (93)
42. school	+0.050	9.26e+06 (24)	-0.056 (53)	92. Afghanistan	-1.458	2.74e+05 (83)	-0.703 (97)
43. winter	+0.050	1.26e+06 (64)	+0.101 (24)	93. gun	-1.476	6.81e+05 (72)	-0.367 (92)
44. rain	+0.050	3.23e+06 (41)	-0.134 (63)	94. hate	-1.520	9.65e+06 (23)	-0.282 (84)
45. Stephen Colbert	+0.001	2.38e+04 (99)	+0.126 (21)	95. depressed	-1.541	2.81e+05 (82)	-0.339 (89)
46. vegan	-0.015	1.84e+05 (90)	+0.315 (3)	96. hell	-1.551	6.27e+06 (30)	-0.250 (79)
47. church	-0.016	1.81e+06 (58)	+0.131 (19)	97. sick	-1.630	3.58e+06 (37)	-0.262 (80)
48. Jon Stewart	-0.024	5.21e+04 (97)	-0.052 (52)	98. headache	-1.881	8.57e+05 (69)	-0.437 (94)
49. I	-0.062	3.08e+08 (2)	-0.048 (49)	99. flu	-1.912	9.01e+05 (68)	-0.735 (99)
50. I feel	-0.129	5.17e+06 (31)	-0.173 (70)	100. war	-2.040	1.96e+06 (57)	-0.270 (82)

TABLE S1: The same selection of 100 keywords and text elements listed in the main text's Tab. 2, reordered by normalized happiness $h_{\rm avg}^{\rm (norm)}$.
